# Comparative Chemical Profiling and Monacolins Quantification in Red Yeast Rice Dietary Supplements by ^1^H-NMR and UHPLC-DAD-MS

**DOI:** 10.3390/molecules25020317

**Published:** 2020-01-13

**Authors:** Rabab Hachem, Gaëtan Assemat, Stéphane Balayssac, Nathalie Martins-Froment, Véronique Gilard, Robert Martino, Myriam Malet-Martino

**Affiliations:** 1 Biomedical NMR Group, Laboratoire SPCMIB, UMR CNRS 5068, Université Paul Sabatier, 118 route de Narbonne, 31062 Toulouse CEDEX 9, France; Rabab.Hachem@evotec.com (R.H.); gaetan-ass@hotmail.fr (G.A.); balayssac@chimie.ups-tlse.fr (S.B.); gilard@chimie.ups-tlse.fr (V.G.); rmartino@chimie.ups-tlse.fr (R.M.); 2 Service commun de spectrométrie de masse, Institut de Chimie de Toulouse, Université Paul Sabatier, 118 route de Narbonne, 31062 Toulouse CEDEX 9, France; martins@chimie.ups-tlse.fr

**Keywords:** red yeast rice, ^1^H-NMR, UHPLC-DAD-MS, mass spectrometry, monacolins

## Abstract

Red yeast rice dietary supplements (RYR DS) are largely sold in Western countries for their cholesterol-lowering/regulating effect due to monacolins, mainly monacolin K (MK), which is, in fact, lovastatin, the first statin drug on the market. ^1^H-NMR was used as an easy, rapid and accurate method to establish the chemical profiles of 31 RYR DS and to quantify their monacolin contents. Among all the ^1^H resonances of the monacolins found in RYR, only those of the ethylenic protons of the hexahydronaphthalenic ring at 5.84 and 5.56 ppm are suitable for quantification because they show no overlap with the matrix signals. The total content in monacolins per capsule or tablet determined in 28 DS (the content in 3 DS being below the limit of quantification of the method, ≈ 0.25 mg per unit dose) was close to that measured by UHPLC, as shown by the good linear correlation between the two sets of values (slope 1.00, y-intercept 0.113, r^2^ 0.986). Thirteen of the 31 RYR DS analyzed (i.e., 42%) did not provide label information on the concentration of monacolins and only nine of the 18 formulations with an indication (i.e., 50%) actually contained the declared amount of monacolins.

## 1. Introduction

The *Monascus*-fermented rice is the fermentation product of non-glutinous white rice with the fungus *Monascus*. It has been used for more than a thousand years in East Asian countries for producing rice wine, for flavoring, coloring and preservation of foods as well as a folk medicine for improving food digestion and blood circulation. Several species other than that isolated in 1895 and named *Monascus purpureus* in recognition of its purple colour, have also been widely used in making red wine and red-coloured foods [1]. These *Monascus* fermentation products are called Red Yeast Rice (RYR) in Western countries although the designation “yeast” is incorrect as *Monascus* is a filamentous fungus and not a yeast [2]. Other more accurate denominations like red fermented rice or red mold rice are also used but much more rarely. Today, the usage of *Monascus* rice products as colorant or flavour in foods, and for brewing red rice wine is permitted in many Asian countries but not in Europe. However, RYR extracts are largely sold in Western countries for their cholesterol-lowering effects.

A multitude of fungal secondary metabolites, phytosterols, isoflavonoids, fatty acids, pigments, monacolins and others, are produced during the fermentation process. Monacolins, in particular monacolin K (MK), which is, in fact, lovastatin, the first marketed statin drug, inhibit the activity of 3-hydroxy-3-methylglutaryl coenzyme A (HMG-CoA) reductase. As a result, the endogenous synthesis of cholesterol is reduced and hence the elevated cholesterol level decreases. Many clinical studies demonstrated the efficacy of RYR in the treatment of hypercholesterolemia and its relative safety [3,4]. For similar cholesterol level reduction, the MK amount in clinically tested RYR is markedly lower than that used with prescription statin drugs [4]. This RYR potency is likely explained by the presence of monacolins other than MK and also by the improved dissolution rate and bioavailability of lovastatin when given as RYR [4,5]. However, adverse effects following RYR consumption have been reported; the nature of the symptoms and the targeted organs and systems are similar to those reported for statin drugs [6,7,8,9]. Considering the case reports gathered in the WHO Vigibase (82) [8] and those collected by four national health institutions (FDA (164) [8], French Nutrivigilance system (30) [6], Italian Surveillance system (52) [7] and Netherlands Pharmacovigilance Centre Lareb (74) [9]), the more frequent adverse effects are: (i) myalgias and related neuroskeletal complaints from 30% of FDA cases to 43% of Lareb cases, including cases of rhabdomyolysis in the five registries, (ii) gastrointestinal disorders which concern up to 23% of Italian cases, (iii) hepatobiliary disorders that affect from 9% of the WHO cases to 32% of the French cases, including severe adverse reactions as pancreatitis and acute hepatic failure, and (iv) skin and subcutaneous disorders which concern from 8% of French cases to 17% of Italian cases.

Nowadays, RYR is widely used as a cholesterol-lowering agent by patients with a proven or perceived intolerance to statins or by consumers, even without dyslipidemia or increased cardiovascular risk, interested in complementary and alternative medications to influence their lipid levels, as it is a common belief that “natural” products do not have side effects [4]. The RYR products are registered as dietary supplements (DS) and, despite their ever growing popularity, there is no uniform regulation regarding their content in monacolins, especially in MK, nor strict quality control. So, their efficacy and safety are unpredictable. Therefore, the development of analytical methods for the simultaneous determination of MK and other monacolins in RYR products is of great importance.

High performance or Ultra-high performance liquid chromatography (HPLC or UHPLC) with diode array detection (DAD) and/or mass spectrometry (MS) detection are regarded as the gold standard methods for the accurate identification and quantification of a wide range of components in RYR products, including monacolins and pigments [10,11,12,13,14]. Although sensitive and selective, they are usually time-consuming, require standard reference materials for quantitative analysis and may suffer from the occurrence of co-eluting interferences (matrix effects) which is a major drawback for MS quantification [15]. Proton Nuclear Magnetic Resonance (^1^H-NMR) is recognized as a method of choice for the analysis of complex mixtures (pharmaceuticals, biological media for instance) [16,17]. Indeed, it is highly reproducible, robust, and nonselective, thus allowing an unbiased overview of the sample composition as all the low molecular weight compounds in the solution (provided they bear ^1^H nuclei and are present at sufficient concentration) are detected simultaneously in a single run. It is also inherently quantitative because the area of each NMR resonance is directly proportional to the number of corresponding nuclei if spectra are recorded in fully relaxed conditions. Thus, at variance with other techniques, the response factor is not dependent on the molecular structure and there is no need for identical reference materials. Moreover, the sample preparation for NMR analysis is very simple as it requires dissolution (for solid products) or dilution (for liquid products) in an adequate deuterated solvent [16]. Lachenmeyer et al. [18] have already used ^1^H-NMR for the assay of the total quantity of monacolins in five RYR commercial products. They showed that the inhibitory effect of RYR on HMG-CoA reductase was all the more important as their monacolin content was high, but the monacolin contents they determined were not compared to those measured by an orthogonal analytical method.

The purpose of this study was to validate (or not) the ^1^H-NMR method for an accurate determination of the monacolin content in RYR DS by comparison with the well-established HPLC method and thus to control their quality. Therefore, 31 RYR DS were analyzed using ^1^H-NMR to establish their spectral signatures and to determine their monacolin contents based on the quantification of selected protons characteristic of the different monacolin chemical structures usually present. An UHPLC analysis with UV-Visible (UV-Vis) and MS detection was performed in parallel on the same 31 RYR DS in order to determine their chemical profiles and to quantify all the monacolins identified.

## 2. Results and Discussion

### 2.1. ^1^H-NMR Analysis

Thirty-one RYR DS were analyzed by ^1^H-NMR. All samples with their name, origin, form, batch number, expiration date and RYR extract content are listed in Table 1.

#### 2.1.1. Qualitative ^1^H-NMR Analysis

**Figure 1:**
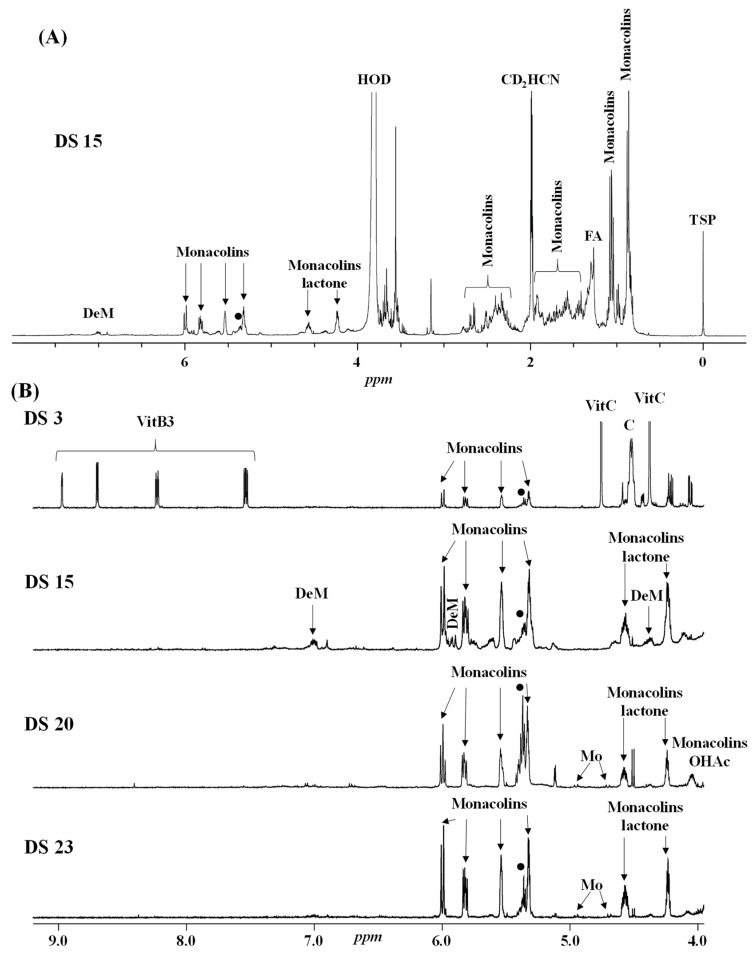
^1^H-NMR spectra of selected RYR dietary supplements (DS) recorded in CD_3_CN:D_2_O (80:20). Entire spectrum of DS **15** (**A**) and enlarged downfield region (4–9 ppm) (**B**) of the DS **3**, **15**, **20** and **23**. DeM: dehydromonacolins, Monacolins lactone: monacolins in lactone form, Monacolins OHAc: monacolins in hydroxyl acid form, Mo: monascin and other pigments with the same skeleton, FA: fatty acids (saturated and unsaturated), ●: non-conjugated unsaturated fatty acids, C: carnitine, Vit: vitamin, TSP: sodium 2,2,3,3-tetradeutero-3-(trimethylsilyl) propanoate, set at 0 ppm.

**Figure 2:**
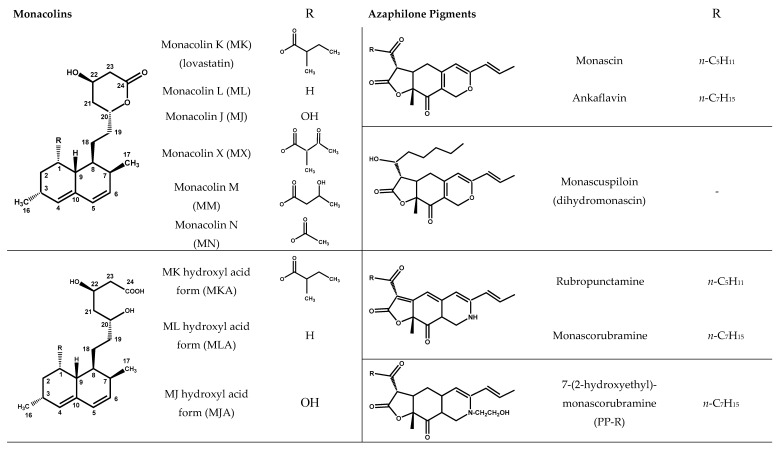

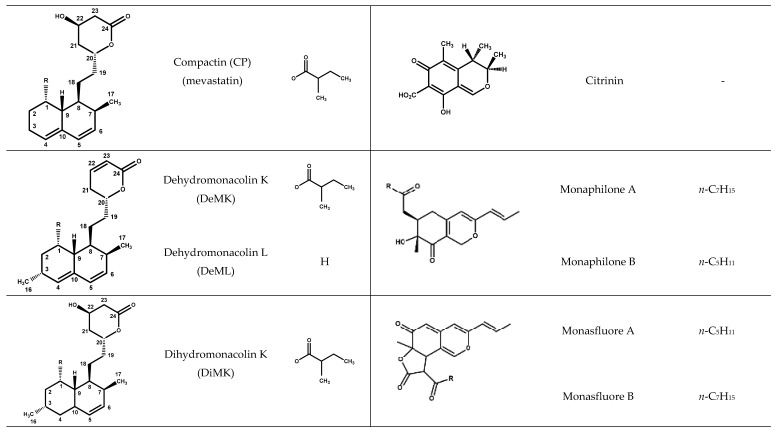
Chemical structures of monacolins and pigments discussed in this study.

Four characteristic spectra are illustrated in Figure 1 (the ^1^H-NMR spectra of all RYR DS analyzed are shown in Figure S1). The monacolin resonances were identified by comparing the ^1^H-NMR spectra of RYR DS with those of standard monacolins whose chemical structures are respectively characteristic of monacolins in lactone form (MK and compactin (CP)), monacolins in hydroxyl acid form (MKA), dehydromonacolins (DeMK) and dihydromonacolins (DiMK), the main monacolin derivatives found in RYR (see Figure 2 for chemical structures) [10,12,13]. Their ^1^H-NMR assignments are given in Table 2. A complete one-dimensional (1D) and two-dimensional (2D) description of the ^1^H and ^13^C-NMR signals of standard MK, MKA, CP and DiMK is presented in Table S1.

**Figure 3:**
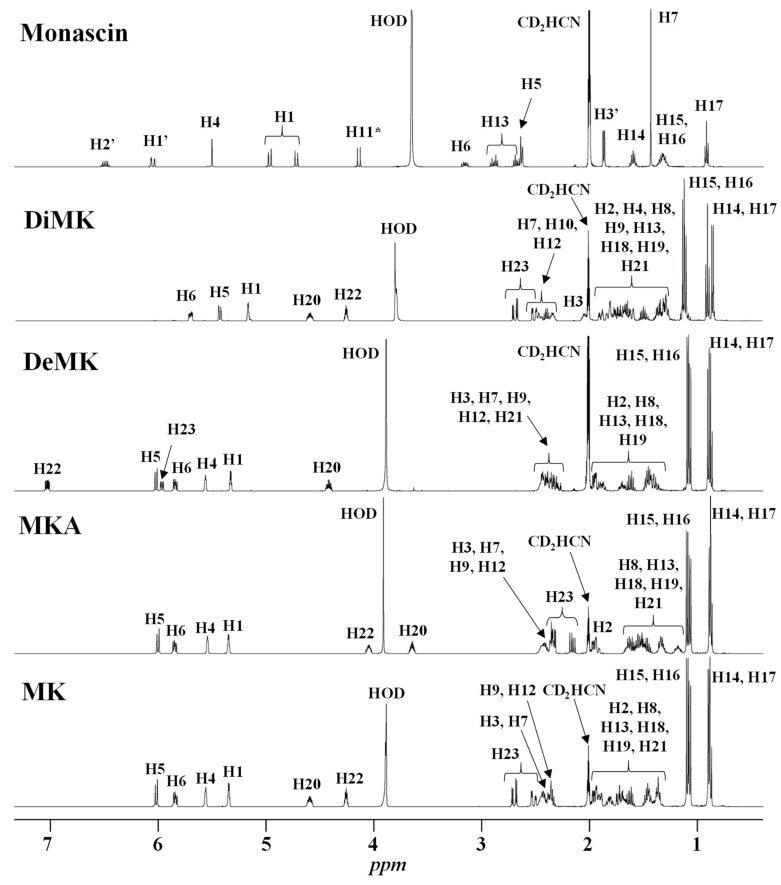
^1^H-NMR spectra of standards of monacolin K in lactone form (MK) and in hydroxyl acid form (MKA), dehydromonacolin K (DeMK), dihydromonacolin K (DiMK) and monascin recorded in CD_3_CN:D_2_O (80:20). The chemical structures of all the compounds and their protons numbering are given in Table 2. (*) The signal of H11 of monascin disappears with time due to exchange with D_2_O.

The resonances of the hexahydronaphthalene ring ethylenic protons (H5, H6, H4 at ≈ 6.01, 5.84 and 5.56 ppm, respectively) are representative of all the monacolins usually found in RYR, except dihydromonacolins as the δ of the ethylenic protons H5 and H6 of DiMK resonated at 5.42 and 5.69 ppm, respectively (Figure 1 and Figure 3, Table 2). The H1 signal (q) at ≈ 5.33 ppm is characteristic of monacolins with a hexahydronaphthalene ring and an ester group (O-CO-R) in position 1. Indeed, for the monacolins with an OH (monacolin J (MJ) and derivatives) or H substituent (monacolin L (ML) and derivatives) instead of ester (Figure 2), the H1 proton(s) resonated respectively at ≈ 4.24 ppm or 1.17 and 1.77 ppm [1,19]. The H20 multiplet at ≈ 4.60 ppm is characteristic of all the monacolins in lactone form including dihydromonacolins but not of monacolins in hydroxyl acid form and dehydromonacolins (Table 2, Figure 3). The H22 signal at ≈ 4.25 ppm is also characteristic of all the monacolins in lactone form (Table 2, Figure 3).

All these monacolin resonances mainly arise from MK, the main monacolin present in RYR DS [1] but other characteristic signals of monacolins were also identified: dehydromonacolins (H22 at 7.03 ppm), dihydromonacolins (H6 at 5.69 ppm) and monacolins in hydroxyl acid form (H22 at 4.05 ppm and H20 at 3.63 ppm) (Figure 1B for some signals).

The singlet of the H1 of citrinin at 5.94 ppm and 8.45 ppm (see footnote 4 of Table 2 for explanation) was never observed, which is not surprising due to the expected very low amount of this compound even if it could have been present [12,13].

Beside the assignments of the different monacolin families, ^1^H characteristic signals of many other compounds mentioned or not on the label of the RYR DS were detected (Table 2). Fatty acids, both saturated (SFA) and non-conjugated unsaturated (UFA), were found in all the formulations (Figure 1 and Table 3) as it has been reported that they represent ≈ 3% of RYR extracts, each group in approximately identical proportion (≈1.4%) [1]. The presence of glycerol and glucose was observed in 25 and 24 samples, respectively. The singlet at ≈5.50 ppm characteristic of the H4 of monascin and/or other pigments with the same skeleton (ankaflavin, monascuspiloin, monaphilones A or B) (Figure 2) was observed in 20 formulations. Some other compounds, generally mentioned on the label of the RYR DS, were detected in few samples: sorbitol and piperine in two samples as well as carnitine, vitamin B3, vitamin C, chlorogenic acid and isopropyl alcohol in one sample (Table 2 and Table 3).

**Table 2:** Structures and ^1^H-NMR characteristics (solvent: CD_3_CN:D_2_O 80:20) of standard monacolins and other compounds identified in this study.

Compound	Structure	^1^H-NMR ^1^
		δ (ppm) (Multiplicity ^2^, *J* (Hz), Number of Protons, Attribution)
Monacolin K lactone form (MK)	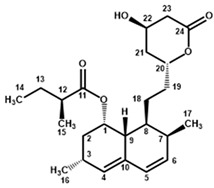	6.01 (d, *J* = 9.6, 1H, H-5), 5.84 (dd, *J* = 6.1, 9.6, 1H, H-6), 5.56 (app t, *J* = 2.8, 1H, H-4), 5.35 (q, *J* = 3.2, 1H, H-1), 4.59 (m, 1H, H-20), 4.25 (app quint, *J* = 3.9, 1H, H-22), 2.69 (Ad, *J* = 4.9, 17.6, 1H, H-23), 2.51 (Bdd, *J* = 1.7, 3.8, 17.6, 1H, H-23), 2.45 (m, 1H, H-3), 2.42 (m, 1H, H-7), 2.37 (m, 1H, H-9), 2.35 (m, 1H, H-12), 1.96 (m, 2H, H-2), 1.90 and 1.71 (two m, 2H, H-21), 1.81 and 1.37 (two m, 2H, H-19), 1.69 (m, 1H, H-8), 1.62 (app qd, *J* = 7.4, 13.6, 1H, H-13), 1.46 (m, 2H, H-13 and H-18), 1.36 (m, 1H, H-18), 1.08 (d, *J* = 6.9, 3H, H-15), 1.06 (d, *J* = 7.4, 3H, H-16), 0.89 (d, *J* = 6.9, 3H, H-17), 0.88 (t, *J* = 7.5, 3H, H-14)
Monacolin K hydroxyl acid form (MKA)	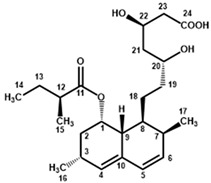	5.99 (d, *J* = 9.6, 1H, H-5), 5.83 (dd, *J* = 6.1, 9.6, 1H, H-6), 5.53 (app t, *J* = 2.8, 1H, H-4), 5.33 (q, *J* = 3.2, 1H, H-1), 4.05 (m, 1H, H-22), 3.63 (app hept, *J* = 4.1, 1H, H-20), 2.42 (m, 1H, H-3), 2.39 (m, 1H, H-7), 2.35 (m, 1H, H-9), 2.33 (m, 2H, H-12 and H-23), 2.16 (dd, *J* = 8.7, 15.2, 1H, H-23), 1.93 (m, 2H, H-2), 1.63 (m, 1H, H-8), 1.59 and 1.45 (two m, 2H, H-13), 1.57 and 1.51 (two m, 2H, H-21), 1.53 and 1.15 (two m, 2H, H-19), 1.32 (m, 2H, H-18), 1.08 (d, *J* = 6.9, 3H, H-15), 1.05 (d, *J* = 7.4, 3H, H-16), 0.87 (d, *J* = 6.9, 3H, H-17), 0.86 (t, *J* = 7.4, 3H, H-14)
Compactin (CP) = Mevastatin	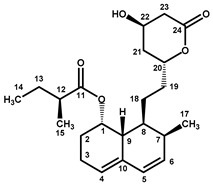	6.00 (d, *J* = 9.7, 1H, H-5), 5.79 (dd, *J* = 6.0, 9.7, 1H, H-6), 5.57 (m, 1H, H-4), 5.30 (m, 1H, H-1), 4.60 (m, 1H, H-20), 4.25 (app quint, *J* = 3.9, 1H, H-22), 2.69 (Ad, *J* = 4.8, 17.6, 1H, H-23), 2.51 (Bdd, *J* = 1.7, 3.6, 17.6, 1H, H-23), 2.42 (m, 2H, H-7 and H-9), 2.38 (m, 1H, H-12), 2.15 (m, 2H, H-3), 2.08 (m, 1H, H-2), 1.90 (m, 1H, H-21), 1.81 and 1.37 (two m, 2H, H-19), 1.72 (m, 2H, H-2 and H-21), 1.68 (m, 1H, H-8), 1.62 (app qd, *J* = 7.6, 13.6, 1H, H-13), 1.48 and 1.39 (two m, 2H, H-18), 1.46 (m, 1H, H-13), 1.11 (d, *J* = 7.0, 3H, H-15), 0.90 (d, *J* = 7.0, 3H, H-17), 0.89 (t, *J* = 7.5, 3H, H-14)
Dehydromonacolin K (DeMK)	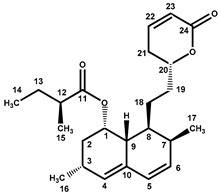	7.03 (ddd, *J* = 2.5, 6.0, 9.8, 1H, H-22), 6.02 (d, *J* = 9.6, 1H, H-5), 5.97 (ddd, *J* = 1.0, 2.7, 9.8, 1H, H-23), 5.84 (dd, *J* = 6.1, 9.6, 1H, H-6), 5.56 (app t, *J* = 2.8, 1H, H-4), 5.33 (q, *J* = 3.2, 1H, H-1), 4.42 (m, 1H, H-20), 2.44 (m, 1H, H-3), 2.43 (m, 1H, H-7), 2.42 and 2.30 (two m, 2H, H-21), 2.36 (m, 1H, H-9), 2.35 (m, 1H, H-12), 1.96 (m, 2H, H-2), 1.90 and 1.41 (two m, 2H, H-19), 1.70 (m, 1H, H-8), 1.62 (app qd, *J* = 7.5, 13.8, 1H, H-13), 1.46 (m, 1H, H-13), 1.45 and 1.39 (two m, 2H, H-18), 1.09 (d, *J* = 6.9, 3H, H-15), 1.07 (d, *J* = 7.4, 3H, H-16), 0.89 (d, *J* = 6.9, 3H, H-17), 0.88 (t, *J* = 7.5, 3H, H-14)
Dihydromonacolin K (DiMK)	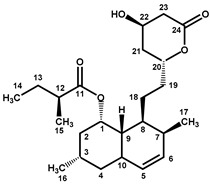	5.69 (ddd, *J* = 2.7, 5.0, 9.8, 1H, H-6), 5.42 (d, *J* = 9.8, 1H, H-5), 5.15 (q, *J* = 2.7, 1H, H-1), 4.58 (m, 1H, H-20), 4.25 (app quint, *J* = 3.8, 1H, H-22), 2.68 (Ad, *J* = 4.9, 17.7, 1H, H-23), 2.50 (Bdd, *J* = 1.7, 3.7, 17.7, 1H, H-23), 2.46 (m, 1H, H-10), 2.39 (m, 1H, H-12), 2.34 (m, 1H, H-7), 2.05 (m, 1H, H-3), 1.88 and 1.72 (two m, 2H, H-21), 1.81 and 1.37 (two m, 2H, H-19), 1.80 (m, 2H, H-2), 1.66 (m, 1H, H-8), 1.64 (m, 1H, H-13), 1.61 and 1.35 (two m, 2H, H-4), 1.49 (app qd, *J* = 7.4, 13.6, 1H, H-13), 1.35 and 1.30 (two m, 2H, H-18), 1.12 (d, *J* = 7.0, 3H, H-15), 1.11 (d, *J* = 7.6, 3H, H-16), 0.90 (t, *J* = 7.5, 3H, H-14), 0.86 (d, *J* = 7.0, 3H, H-17)
Monascin ^3^	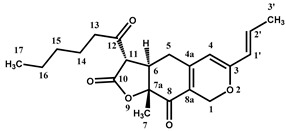	6.50 (qd, *J* = 7.0, 15.5, 1H, H-2′), 6.05 (qd, *J* = 1.7, 15.5, 1H, H-1′), 5.51 (s, 1H, H-4), 4.97 (At, *J* = 0.9, 12.7, 1H, H-1), 4.72 (Bt, *J* = 1.4, 12.7, 1H, H-1), 4.14 (d, *J* = 13.3, 1H, H-11), 3.15 (m, 1H, H-6), 2.88 (At, *J* = 7.3, 18.2, 1H, H-13), 2.67 (Bt, *J* = 7.3, 18.2, 1H, H-13), 2.63 (app br d, *J* = 7.7, 2H, H-5), 1.87 (dd, *J* = 1.7, 7.0, 3H, H-3′), 1.59 (quint, *J* = 7.3, 2H, H-14), 1.43 (s, 3H, H-7), 1.32 (m, 4H, H-15, H-16), 0.91 (t, *J* = 7.2, 3H, H-17)
Citrinin ^4^	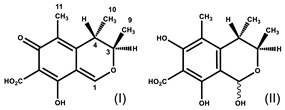	8.45 (br s, H-1 (I)), 5.94 (s, H-1 (II major), 5.90 (br s, H-1 (II minor)), 4.92 (br signal, H-3 (I)), 4.09 (quint, *J* = 6.6, H-3 (II major and minor)), 3.15 (br signal, H-4 (I)), 2.76 (br signal, H-4 (II minor)), 2.67 (quint, *J* = 6.8, H-4 (II major)), 2.03 (s, H-11 *), 2.01 (s, H-11 *), 2.00 (s, H-11 *), 1.30 (d, *J* = 6.7, H-9 *), 1.27 (d, *J* = 6.4, H-9 *), 1.20 (d, *J* = 6.9, H-10 *), 1.17 (very br signal, H-10 *)
Piperine	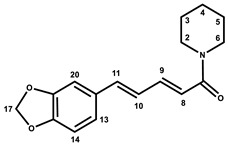	7.28 (ddd, *J* = 2.7, 7.3, 14.7, 1H, H-9), 7.11 (d, *J* = 1.6, 1H, H-20), 6.99 (dd, *J* = 1.7, 8.1, 1H, H-13), 6.87 (d, *J* = 8.0, 1H, H-14), 6.83 (m, 2H, H-10, H-11), 6.60 (d, *J* = 14.7, 1H, H-8), 6.00 (s, 2H, H-17), 3.55 (m, 4H, H-2, H-6), 1.65 (m, 2H, H-4), 1.56 (m, 4H, H-3, H-5)
Carnitine	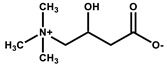	4.56 (br q, *J* = 7.1, 1H, CH-OH), 3.39 (m, 2H, CH_2_-N^+^), 3.17 (s, 9H, (CH_3_)_3_-N^+^), 2.58 (m, 2H, CH_2_-COO^−^)
Chlorogenic acid	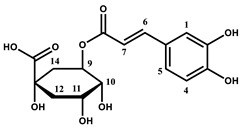	7.61 (d, *J* = 15.9, 1H, H-6), 7.16 (d, *J* = 2.0, 1H, H-1), 7.06 (dd, *J* = 2.0, 8.2, 1H, H-5), 6.88 (d, *J* = 8.2, 1H, H-4), 6.34 (d, *J* = 15.9, 1H, H-7), 5.28 (ddd, *J* = 4.6, 9.4, 10.5, 1H, H-9), 4.18 (app q, *J* = 3.5, 1H, H-10), 3.75 (dd, *J* = 3.2, 9.4, 1H, H-11), 2.26–1.97 (m, 4H, H-12, H-14)
Saturated fatty acids	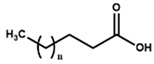	2.20 (t, *J* = 7.5, 2H, CH_2_-COOH), 1.59 (m, 2H, CH_2_-CH_2_-COOH), 1.30 (m, 2nH_,_ (CH_2_)n), 0.90 (t, *J* = 6.3, 3H, CH_3_)
α/β Glucose	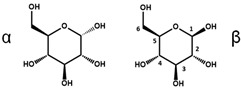	5.14 (d, *J* = 3.7, 1H, H-1α), 4.52 (d, *J* = 7.9, 1H, H-1β), 3.85-3.60 (m, 7H, H-3α, H-4α/β, H-6α/β), 3.45–3.28 (m, 4H, H-2α, H-3β, H-5α/β), 3.15 (t, *J* = 8.5, 1H, H-2β)
Glycerol	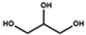	3.66 (tt, *J* = 4.6, 6.3, 1H, CH), 3.55 (Ad, *J* = 4.6, 11.5, 2H, CH_2_), 3.48 (Bd, *J* = 6.2, 11.5, CH_2_)
Isopropyl alcohol	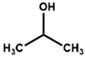	3.93 (hept, *J* = 6.1, 1H, CH), 1.14 (d, *J* = 6.1, 6H, CH_3_)
Linoleic acid ^5^	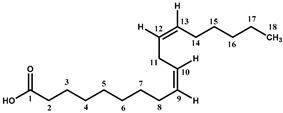	5.36 (m, 4H, H-9, H-10, H-12, H-13), 2.78 (t, *J* = 6.7, 2H, H-11), 2.27 (t, *J* = 7.5, 2H, H-2), 2.06 (m, 4H, H-8, H-14), 1.56 (m, 2H, H-3), 1.40-1.27 (m, 14H, H-4, H-5, H-6, H-7, H-15, H-16, H-17), 0.89 (t, *J* = 6.8, 3H, H-18)
Sorbitol	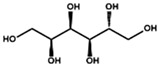	3.82–3.54 (m, 8H)
Vitamin B3 (niacinamide form)	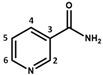	8.99 (d, *J* = 2.3, 1H, H-2), 8.72 (dd, *J* = 4.9, 1.6, 1H, H-6), 8.25 (td, *J* = 8.0, 1.9, 1H, H-4), 7.56 (dd, *J* = 8.0, 4.9, 1H, H-5)
Vitamin C	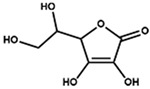	4.76 (d, X part of an ABMX system, J_XM_ = 2.0, 1H, CH-O-), 3.94 (td, M part of an ABMX system, *J*_MX_ = 1.9, *J*_MA_ = *J*_MB_ = 6.6, 1H, CH-OH), 3.66 (AB part of an ABMX system, *J*_AB_ = 11.3, *J*_AM_ = *J*_BM_ = 6.6, 2H, CH_2_-OH)

^1^ The assignments of ^1^H-NMR signals of monacolins, citrinin, and monascin were in agreement with the literature data even if the solvents were different: CDCl_3_ for MK, MKA, CP, DeMK, DiMK and citrinin [1,20,21,22,23,24], DMSO-d6 for monascin [25] and D_2_O for citrinin [22]. ^2^ d: doublet, dd: doublet of doublet, ddd: doublet of doublet of doublet, t: triplet, td: doublet of triplet, tt: triplet of triplet, q: quadruplet, qd: doublet of quadruplet, quint: quintuplet, hept: heptuplet, m: multiplet, A: part A of an AB system, B: part B of an AB system, app: apparent, br: broad. ^3^ H11 is exchanged with D and its resonance disappears rapidly with time as well as its coupling with H6 whose multiplet becomes a triplet (*J* = 7.9 Hz). ^4^ The attributions were done according to the literature [22]. Citrinin exists as the quinone methide (I) in organic NMR solvents but as a diastereoisomeric mixture of hydrates (II) in aqueous solution at physiological pH, one diastereoisomer being major and the other minor. As the solvent used in the present study is a CD_3_CN:D_2_O (80:20) mixture, the two forms (I) and (II) are observed. * From the literature data, it was not possible to assign the resonances of H9, H10 and H11 to a specific form. ^5^ As a model of non-conjugated unsaturated fatty acids.

#### 2.1.2. Quantitative ^1^H-NMR Analysis

With our experimental conditions, the limit of detection (LOD; for a signal-to-noise ratio (SNR) = 3) of monacolin derivatives in real samples was estimated between 4 × 10^−5^ and 10^−4^ M depending on the multiplicity of the targeted proton signal, corresponding respectively to 0.016 and 0.04 mg of monacolins (considered with the molecular weight of MK (404 g mol^−1^)) in the volume of solvent used for the extraction of 20 to 100 mg of RYR formulation. The limit of quantification (LOQ; SNR = 10) for the same signals was evaluated between 10^−4^ and 3 × 10^−4^ M, corresponding respectively to 0.04 and 0.12 mg of monacolin derivatives in the amount of powdered RYR extracted. Considering the ratio between the mass of sample extracted and that of the formulation (tablet or powder in the capsule), the LOQ was estimated to be ≈0.25 mg of monacolin per capsule or tablet.

**Figure 4:**
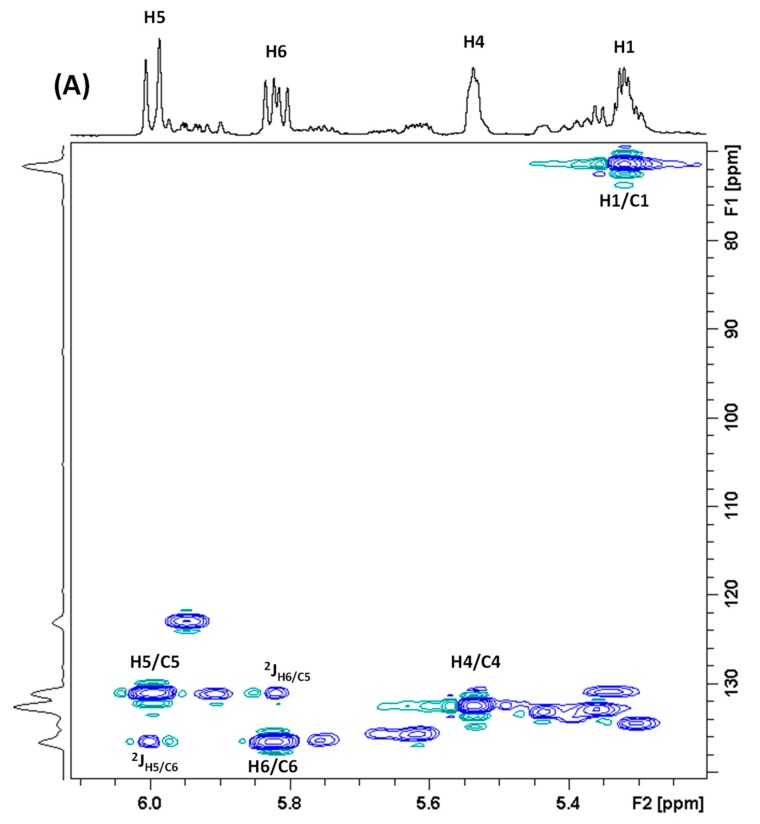

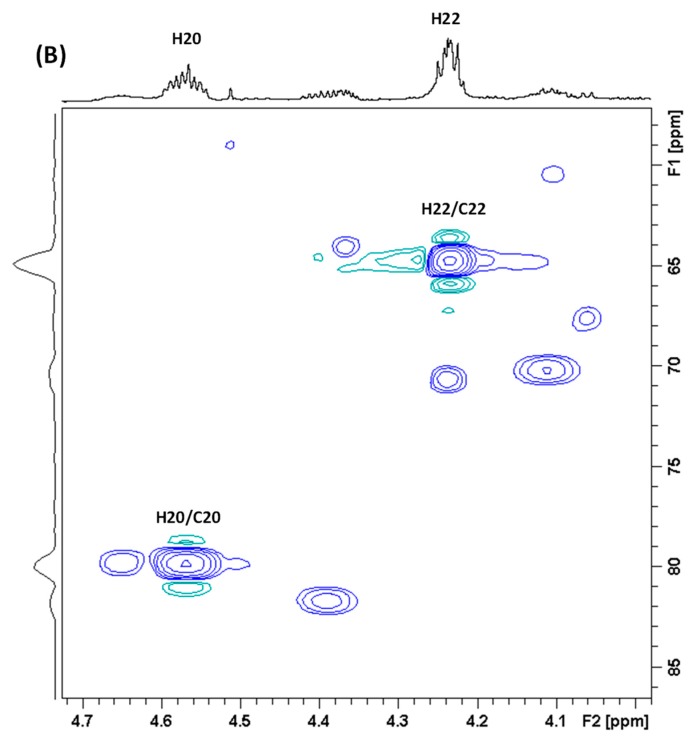
2D ^1^H-^13^C HSQC-NMR spectrum of the dietary supplement **15** recorded in CD_3_CN:D_2_O (80:20). (**A**) ^1^H enlarged region 5.2–6.1 ppm. (**B**) ^1^H enlarged region 3.98–4.72 ppm. The proton numbering of monacolins is given in Table 2.

The H5 resonance (6.01 ppm) was not used for the quantification of monacolins as it may also contain part of the signal (ddd) of the H23 of DeMK at 5.97 ppm and can be slightly overlapped with the H1′ signal (qd) of monascin at 6.05 ppm. The H6 (5.84 ppm) and H4 (5.56 ppm) resonances are not hindered by signals of other molecules and they have been quantified respectively in 27 and 28 RYR DS as no monacolin signal was detected in formulations **11**, **12** and **29** whereas both resonances were observed in DS **16** where only the H4 signal could be quantified with an intensity at the LOQ level. The amount of monacolins in lactone form was determined from either H20 or H22 or both signals in only 22 RYR DS because of significant overlap with matrix signals in some formulations. It was possible to quantify the H1 signal (5.33 ppm) in only 17 formulations due to its overlap with the large resonance of the ethylenic protons of non-conjugated UFA at ≈5.36 ppm and maybe also of other compounds. The 2D ^1^H-^13^C HSQC-NMR experiment recorded for the DS **15** and illustrated in Figure 4 clearly shows these findings.

The intensity of the DeMK ddd resonance (H22 at 7.03 ppm) was low or very low and overlapped with matrix signals in most sample spectra and could not thus be quantified precisely. The dd resonance of DiMK at 5.69 ppm was not clearly detected in any formulation and could not thus be quantified. The signal intensities of monacolins in hydroxyl acid form (H22 at 4.05 ppm and H20 at 3.63 ppm) would have allowed their quantification in most of the formulations but their substantial overlap with the resonances of matrix compounds did not permit to obtain accurate values without curve deconvolution treatment.

The monacolin contents determined by ^1^H-NMR are reported in Table 3. For more clarity, the coefficient of variation (CV) and the relative standard deviations (RSD) are given in Table S2. The RSD of the ^1^H-NMR assays ranged between 1.5% and 13%. The relevance of the ^1^H-NMR data will be discussed later when compared to those obtained by UHPLC.

### 2.2. Chromatographic Analysis

#### 2.2.1. Identification of Monacolins and Pigments

RYR formulations were traced by UHPLC-DAD-MS. Among the great number of compounds observed, 23 (12 monacolins and 11 azaphilones; see Figure 2 for their chemical structures) were identified by comparing with the literature their elution sequence, their UV-Vis and MS profiles as well as the accurate mass measurements of their parent and fragment ions in High Resolution Electrospray Ionization (HR-ESI) MS and MS/MS [13,26]. Moreover, the identification of MK, CP, MKA, DeMK, DiMK, citrinin and monascin was confirmed by comparing their UV-Vis and MS and MS/MS chromatographic profiles with those of the corresponding standards. The retention time (tR), UV-Vis λ_max_, accurate masses of parent ion and of its MS/MS major fragment ions for each compound identified are gathered in Table 4. We will not describe the process that allowed the identification of the 23 compounds but we will show the respective contributions of UV-Vis and HR-MS and MS/MS to determine their chemical structure through some selected examples.

The UV-Vis absorption spectra gave some indication on the chemical structure of the compounds detected. For example, all the monacolins with a conjugated hexahydronaphthalene ring displayed the same characteristic mountain-like UV spectrum with three maximum absorptions at ≈ 230, 238 and 246 nm (range 229–230, 238–239 and 244–247 nm) (Table 4) in accordance with literature data [1,10,27,28]). Due to the absence of conjugated double bonds, the dihydromonacolins with an octahydronaphthalene ring and so an UV maximum absorption band at ≈210 nm [1,20] were not detected at the wavelength used in this study (238 nm). In the same way, the yellow azaphilone pigments with the classical monascin-type chromophore showed specific profiles with high intensity peaks at 227–233 and 386–392 nm and a very low intensity band at 285–291 nm, while the red ones with the rubropunctamine-type chromophore presented specific absorbances at 251–252, 302–307, 412–423 and 525–530 nm (Table 4), all these values being in agreement with literature reports [29,30,31,32,33,34].

Nevertheless, the unambiguous determination of compound identity required the additional use of HR-MS and HR-MS/MS. For instance, the UV absorption spectrum of the compound eluting at tR 3.89 min with λ_max_ at 230, 239 and 247 nm was characteristic of monacolins. Its protonated molecular ion [M + H]^+^ at *m*/*z* 363.2159 and sodium adduct ion [M + Na]^+^ at *m*/*z* 385.1988 were in agreement with respectively the calculated masses 363.2171 of C_21_H_31_O_5_ (relative mass error (RME) −3.3 ppm) and 385.1991 of C_21_H_30_O_5_Na (RME −0.8 ppm) suggesting a C_21_H_30_O_5_ molecular formula (Table 4). Moreover, the MS/MS product ions obtained were characteristic of the fragmentation pathway of monacolins (Table 4) [13,14,26]. So, the compound detected was a monacolin derivative that might be monacolin N (MN) or dehydromonacolin N hydroxyl acid (DeMNA) recently described by Li et al. [26]. We propose that it was MN based on the fact that its tR at −0.16 min with respect to monacolin L in hydroxyl acid form (MLA) was in agreement with the value given by Li et al. for MN (−0.22 min) and not for DeMNA (+1.72 min). Moreover, DeMNA was detected only at trace level using nanoflow HPLC-Chip-MS whereas MN was also detected by conventional HPLC-MS which is much less sensitive [26].

The identification of the compound eluting at 9.78 min is another example of the interest of coupling tR, UV-Vis profile and HR-MS spectra. This compound was not detected at 238 nm but only by its UHPLC-MS profile exhibiting a molecular ion [M + H]^+^ at *m*/*z* 407. Among the known monacolins, two have a nominal mass of 406: DiMK (C_24_H_38_O_5_) with an exact mass for [M + H]^+^ at *m*/*z* 407.2798 and monacolin M (MM; C_23_H_34_O_6_) with an exact mass for [M + H]^+^ at *m*/*z* 407.2434. The measured mass of the [M + H]^+^ ion at *m*/*z* 407.2796 indicated that the compound was DiMK. Furthermore, its MS/MS fragmentation pattern gave the same ions than MK but with 2 more *m*/*z* units (305, 287, 269… compared to 303, 285, 267…) confirming that a double bond of the fused ring was hydrogenated (Table 4), whereas MS/MS fragmentation behavior of MM is identical to that of MK [26]. Also, MM eluted between MJ and MLA with a relative tR versus MJ of 1.2–1.3 [13,26] and hence, in our UHPLC conditions, MM would be expected at 2.4–2.6 min and not at 9.78 min. The incorrect assignment of this compound to MM by Li et al. in 2004 [10] was based on the mountain-like UV spectrum centred at 238 nm characteristic of classical monacolins and on the MS [M + H]^+^ ion at *m*/*z* 407, but the UV absorption and the [M + H]^+^ peak did not correspond to the same compound. Indeed, the compound with an UV absorbance at 238 nm was very probably dehydromonacolin L (DeML), a monacolin analogue described for the first time in 2011 [37] whose tR is close to that of DiMK (Table 4) [26].

**Figure 5:**
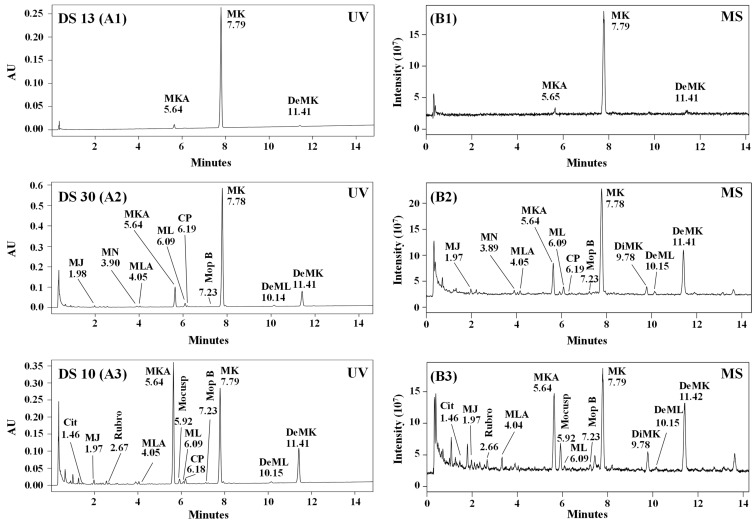

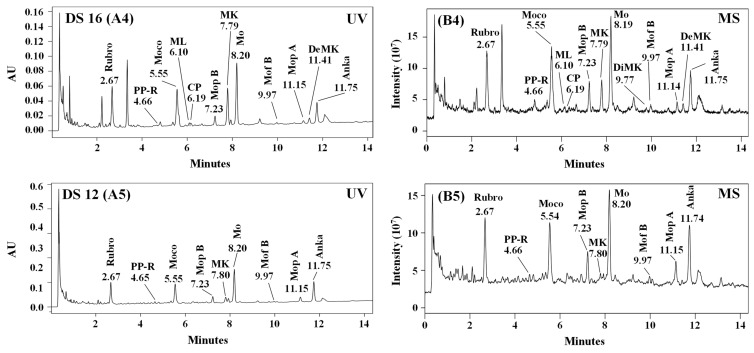
UHPLC chromatograms with UV detection at 238 nm (**A1**–**A5**) and full scan MS profile in positive ESI mode (**B1**–**B5**) of five characteristic RYR DS. MJ, MN, ML, MK: monacolins J, N, L, K; MLA, MKA: monacolins L, K in hydroxyl acid forms; CP: compactin; DiMK: dihydromonacolin K; DeML, DeMK: dehydromonacolins L,K; Anka: ankaflavin; Cit: citrinin; Mo: monascin; Moco: monascorubramine; Mocusp: monascuspiloin; Mof B: monasfluore B; Mop A and Mop B: monaphilone A and monaphilone B; PP-R: 7-(2-hydroxyethyl)-monascorubramine; Rubro: rubropunctamine.

Monacolins and pigments identified in each RYR formulation are listed in Table 5. UHPLC UV-Vis and MS profiles of five characteristic RYR DS, one with only monacolins (formulation **13**), two with mainly monacolins (formulations **10** and **30**) and two with more pigments than monacolins (formulations **12** and **16**) are illustrated in Figure 5 (the UHPLC UV-Vis and MS profiles of nine other RYR DS analyzed are shown in Figure S2). MK was detected in all the samples analyzed and MKA and DeMK in respectively 28 and 29 DS. MLA, ML, CP, DiMK and DeML were found in 18–22 DS, MJ in 11 and MN in 8. The other monacolins were observed only in few samples: one for monacolin J in hydroxyl acid form (MJA) and two for monacolin X (MX). MM cannot be found in any RYR DS. The mycotoxin citrinin was only detected at trace level in two samples. Among the yellow azaphilone pigments, monascin, monascuspiloin and monaphilone B were identified respectively in 18, 15 and 21 of the RYR DS analyzed, whereas ankaflavin, monaphilone A, monasfluore A and B were found in few samples (6, 4, 5 and 3 respectively). The red azaphilone pigments, rubropunctamine, monascorubramine and PP-R, were identified in 11, 4 and 2 samples, respectively.

#### 2.2.2. Quantitative Analysis of Monacolins

The LOD and LOQ of standard lovastatin, established for SNR 3 and 10, were respectively 0.4 × 10^−3^ mg mL^−1^ and 1.5 × 10^−3^ mg mL^−1^ corresponding to 0.4 μg and 1.5 μg in the 10 to 100 mg of powdered.

RYR formulation extracted. Considering the ratio between the mass of sample extracted and that of the formulation (tablet or powder in the capsule), the LOQ was estimated to be ≈0.9 μg of lovastatin or other monacolins per capsule or tablet. The precision of the method was acceptable with a RSD for the replicates less than 2% (range 0.1%–1.9%). The overall average value of RSD was 5% ranging between 2% and 11%.

The amounts of the 12 monacolins identified in the 31 RYR DS are summarized in Table 5. There is a marked variability in total monacolins (TotalM), MK and MK + MKA contents per capsule or tablet with values ranging respectively between 0.07 and 23.84 mg, 0.07 and 23.18 mg and 0.07 and 23.80 mg. MK is by far the main monacolin in the 31 RYR analyzed representing 68% of TotalM (range 37–100%) and the sum MK + MKA representing 82% (range 52%–100%). As MKA is the active form of MK, the European Food Safety Authority (EFSA) considers that the effective MK content in RYR DS corresponds to the sum of both lactone and hydroxyl acid forms [38], which allows to get rid of the great variability of the ratio MK/MKA. Indeed, this ratio was comprised between 0.9 and 37 in 28 of the RYR DS analyzed in the present study and could not be determined for the three others due to the absence of MKA. These values are in agreement with literature data which report ranges from 0.4 to 85 [10,12,13,39] while the ratio varies between 1.5 and 2 in properly prepared RYR [1]. Minor monacolins represent on average 18% of TotalM, ranging from 0 to 47%. All these results fully confirm previous studies that showed that monacolin contents vary considerably in RYR end-use products, as they depend on the yeast strain and the fermentation process. For example, the HPLC analysis of 15 commercial tablets or capsules showed that TotalM ranges between 0.31 and 11.15 mg per 600 mg of RYR [39,40,41,42]. The literature review of the monacolins quantification established that MK represents 57% of TotalM (*n* = 26; range 0%–99%), MK + MKA 83% (*n* = 41; range 32%–100%), and the minor monacolins 17% (*n* = 41; range 0%–68%) [1,10,39,40,41,42,43].

Our own results and all the literature data show that to improve the effectiveness and safety of RYR products in lowering/regulating cholesterol levels, a precise quantification of their monacolin content should be mandatory. Indeed, the MK content indicated for some formulations (18 over the 31 tested) is not sufficient to know the effectiveness of RYR DS as the amount of MKA, which is largely variable, is not specified. However, it is unclear if the term MK used by the manufacturers refers to the sole MK or to the sum MK + MKA. The chemical structures of MK and MKA being different, this should be mentioned. Moreover, the content of minor monacolins should be taken into account as they represent at least ≈ 14% of TotalM in 65% of the RYR tested in our study (Table 5) and in 71% of the 41 RYR products reported in the literature [1,10,39,40,41,42,43]. Indeed, if MK after its transformation by liver metabolism into MKA has the highest HMG-CoA reductase inhibitory activity, the other secondary monacolins such as CP, MJ, ML or DiMK are also active although at a lower or much lower extent (except for DiMK). Relative to MK or DiMK activity stated at 1, those of the other monacolins are 0.5 for CP and dihydroCP, 0.15 for ML, 0.2 for dihydroML, MX and dihydroMX, and 0.04 for MJ [44]. The dehydromonacolins, often considered as inactive, also present a low activity (0.2 for DeMK [45]).

### 2.3. Comparison of ^1^H-NMR and UHPLC Results

^1^H-NMR makes it possible to highlight the overall profile of the compounds present in the RYR DS in a single analysis of the crude extract. Indeed, not only all the monacolins, but also pigments (monascin and other pigments having the same skeleton), fatty acids (SFA and UFA), polyols (glycerol, sorbitol), glucose as well as various other products most often added to the formulation (piperine, carnitine, vitamins) are detected. The main drawback of the method is that the resonances identified are in most cases characteristic of a family of compounds and not of a specific compound as for example, monacolins with a hexahydronaphthalene ring and not only MK, or monascin and other pigments with the same skeleton and not the sole monascin. In contrast, the UHPLC-DAD-MS analysis allows a precise identification of the compounds, but like any separation technique, it requires much more time for implementation and especially the operating conditions used target certain structures of compounds, for example, here, monacolins and pigments.

As the ^1^H-NMR resonances quantified are specific of monacolin families and not of each monacolin, the monacolin amounts determined by ^1^H-NMR must be compared to those obtained by UHPLC-DAD-MS for the same group of monacolins. The relationships between the amounts measured from the quantification of the resonances at 5.84 ppm and 5.56 ppm in 27 and 28 formulations respectively, both characteristic of all the monacolin structures except dihydromonacolins (TotalM-DiMK (because only this dihydromonacolin was detected by UHPLC)) (Table 3), and those obtained by UHPLC-DAD-MS for the same set of monacolins (Table 5) were very good, as demonstrated by slopes near 1, y-intercepts close to zero and correlation coefficients (r^2^) greater than 0.99 for the two linear regression equations as well as p-values of 0.94 and 0.66 (Table S3). A similar very good correlation between NMR and UHPLC values was obtained when considering the mean concentrations measured from the two NMR resonances (Table S3).

The comparison of the contents of monacolins in lactone form determined from the quantification of their characteristic resonances at 4.60 ppm and/or 4.25 ppm in 22 RYR DS to those obtained by UHPLC-DAD-MS for the same panel of monacolins also showed results in good agreement. Indeed, the linear regression equation of the ^1^H-NMR and UHPLC values displayed a slope of 0.983, a y-intercept of 0.013 and a correlation coefficient of 0.996, and the *p*-value was 0.50 (Table S3). The resonance at 5.33 ppm is characteristic of all the monacolins except MJ, MJA, ML, MLA and DiMK. Its quantification, which could only be performed on 17 RYR DS, led to values in agreement within ±11% with those obtained by UHPLC for the same set of monacolins for only six (35%) of them (Table 3 and Table 5). The very weak relationship between the concentrations measured by the two methods was demonstrated by the low correlation coefficient (r^2^ = 0.723) and the y-intercept far from zero of the linear regression equation as well as a *p*-value of 0.02 representative of a significant difference between ^1^H-NMR and UHPLC data (Table S3). Therefore, for the RYR DS analyzed in this study, this resonance is unsuitable for quantification, in contrast to what was observed in a previous ^1^H-NMR assay of five formulations marketed for German consumers [18].

In the same way, the resonances at 4.05 and 3.63 ppm, characteristic of the monacolins in hydroxyl acid form, cannot be accurately quantified due to their strong overlap with matrix signals. Indeed, all the assays tentatively performed led to values considerably higher than those obtained by UHPLC for the same type of monacolins (data not shown).

From all the ^1^H resonances characteristic of the various monacolin chemical structures, only those involving the hexahydronaphthalene ring (at 5.84 and 5.56 ppm) and the lactone ring (at 4.60 and 4.25 ppm) are appropriate for quantification without requiring curve deconvolution algorithms. It should be noted that the quantification of the resonances at 5.84 and 5.56 ppm was hindered by the LOQ of the technique for three formulations (28/31 formulations were quantified), and that of resonances at 4.60 or/and 4.25 ppm was additionally hampered by their overlap with matrix signals in some cases (only 22/31 formulations were quantified). To confirm that the ^1^H-NMR signals at 5.84 and 5.56 ppm can be used to quantify the monacolin content in the RYR formulations, we compared the data obtained to TotalM determined by UHPLC (Table S3). The good relationship between the two sets of values (linear regression equation with a slope of 1.00, a y-intercept at 0.113 and a correlation coefficient of 0.986 as well as a t-test *p*-value of 0.25) was not unexpected as DiMK (the sole dihydromonacolin detected in this study by UHPLC) represented ≈2.8% of the TotalM (range 0–9.9%) (Table 5). The presence of DiMK was not surprising as it is produced in small quantities during the fermentation process of rice with *Monascus* [1,23]. In conclusion, ^1^H-NMR can be considered as a convenient method to determine the TotalM content in RYR DS.

### 2.4. Quality Control Issues

#### 2.4.1. About Monacolin Labelling of RYR DS

Only 18 of the 31 RYR DS (58%) tested specified a monacolin(s) content on their label: 14 indicated a level of MK, 1 the sum MK + MKA, and the label was imprecise for 3 DS, 1 indicating “*Monascus purpureus*” and 2 “monacolin” (Table 6). The amount of MK measured was in the range 90%–110% of declared amount for only 3 of the 14 DS mentioning a quantity of MK (DS **13**, **23** and **27**). If we hypothesize that the label “MK” includes MK + MKA, three additional DS (**15**, **22** and **24**) were in the range 90%–110%. If we consider that the claimed dose corresponds to TotalM for the three DS indicating “*Monascus purpureus*” (DS **4**) or “monacolin” (DS **25** and **30**), the measured amount was between 90% and 110% (^1^H-NMR and UHPLC mean amount for DS **25**) (Table 6). So, 50% of the formulations analyzed contained less than the declared amount of monacolin(s). It is also interesting to note that three DS (**10**, **11** and **12**) contain a very small amount of MK (or MK + MKA) compared to the advertised dose (Table 6). It can thus be concluded that the monacolin label information is not reliable for many RYR products. This deviation between labelled and measured contents has already been reported by Mornar et al. [12] in five DS.

#### 2.4.2. About the Variability of Monacolins Consumed Per Day in RYR DS

There is great variability in the daily consumption of monacolin amounts in terms of MK, MK + MKA, and TotalM (or TotalM-DiMK) calculated from the UHPLC and ^1^H-NMR data taking into account the recommended serving(s) per day indicated on the labels of the RYR products (Table 6). The amounts of MK, MK + MKA and TotalM consumed daily ranged respectively between 0.08 and 46.2 mg, 0.08 and 47.6 mg, 0.08 and 47.7 mg (UHPLC values) and 0.6 (the very low monacolin content in 3 samples (DS **11**, **12** and **29**) being undetected) and 47.8 mg (NMR values). Although the range of monacolins consumed is the same considering MK, MK + MKA or TotalM, this is not true for all formulations. Indeed, MK represented less than 60% of TotalM in 45% (14/31) of the formulations analyzed and MK + MKA accounted for less than 80% of TotalM in ≈40%, emphasizing the helpfulness of the NMR method which allows the content in all monacolins to be easily determined.

About half of the RYR DS analyzed (48%, 15/31) contained more than 7 mg of MK + MKA or 8 mg of TotalM per recommended daily serving, whereas for ≈45% (14/31), the daily intake of MK + MKA or of TotalM was less than ≈ 3 or 4 mg respectively. Although only single batch formulations have been analyzed and the monacolin content may vary from batch to batch, the question of how effective these levels of monacolin can be for lowering/regulating cholesterol levels can be raised. In 2011, the EFSA concluded that a cause and effect relationship has been established between the consumption of 10 mg per day of MK (sum of lactone and hydroxyl acid forms) from RYR DS and the maintenance of normal low-density lipoproteins-cholesterol (LDL-C) levels [38]. However, several clinical trials using RYR products at daily MK doses well below 10 mg (around 5 mg and even 3 mg) without any other cholesterol-lowering agents such as berberine, policosanol or garlic, showed a statistically significant reduction in LDL-C compared to placebo but these low values either corresponded to MK in the sole lactone form [40,42] or it was not indicated whether they included the hydroxyl acid form of MK [3,4,46]. Nevertheless, when the RYR composition in monacolins was available, the amounts of MK + MKA and TotalM were always at least around 7 mg and 10 mg respectively [40,42]. From these data, it can be concluded that a daily dietary intake of ≈7 mg of MK + MKA or ≈8 mg of TotalM is sufficient to induce a cholesterol-lowering/regulating effect. On the other hand, the very low daily intake of monacolins found in ≈ 45% of the RYR DS analyzed might not lead per se to a significant reduction/regulation of cholesterol levels.

## 3. Materials and Methods

### 3.1. Red Yeast Rice Dietary Supplements

Thirty-one RYR DS bought mainly on internet web sites (24) but also from local health food stores (7) between September 2013 and June 2014 were analyzed before their expiration date with UHPLC and ^1^H-NMR techniques. RYR products were formulated as capsules (20) or tablets (11). All samples with their name, origin, form, batch number, expiration date and RYR extract content are listed in Table 1.

### 3.2. Chemicals and Reagents

Authentic standards of lovastatin (MK), citrinin and monascin were purchased from Sigma Aldrich (St. Louis, MO, USA) and those of DeMK, CP and DiMK from TRC (North York, ON, Canada). All other chemicals and reagents used as well the NMR reference for internal chemical shift and quantification (sodium 2,2,3,3-tetradeutero-3-(trimethylsilyl) propanoate (TSP)) were supplied from Sigma Aldrich (St. Louis, MO, USA). Deuterated solvents were obtained from Euriso-Top (91194 Saint Aubin, France). MKA was prepared by hydrolyzing a solution of standard lovastatin in acetonitrile:water (CH_3_CN:H_2_O) or deuterated acetonitrile:deuterated water (CD_3_CN:D_2_O) 80:20 *v*/*v* (3.7 mg mL^−1^) with a 1M NaOH or NaOD solution under the optimized conditions described in literature [12]. The complete conversion of the lactone form (MK) to its acidic form (MKA) was confirmed by HPLC-MS as the [M + H]^+^ peak of MK at *m*/*z* 405 disappeared and the peak of MKA at *m*/*z* 423 was sole detected. Demineralized water was obtained with a Milli-Q system Purelab flex Veolia Waters.

### 3.3. Choice of Extraction Solvent and Preparation of Samples for Analyses

The extraction of monacolins from RYR bulk powders with various solvents such as CH_3_OH, ethanol: water mixtures, CH_3_CN or ethyl acetate has been extensively described, the best results being obtained with CH_3_OH or ethanol:water 75:25 [11,12,13,47,48]. Two extraction solvents were tested in this study, CH_3_OH and CH_3_CN:H_2_O 80:20 (because the MK solubility in CH_3_CN is higher than in ethanol, 28 and 16 mg mL^−1^ respectively) and led to the almost same extraction recovery for all compounds, as measured by UHPLC-DAD. In this study, all sample extractions were performed with the mixture CH_3_CN:H_2_O (or CD_3_CN:D_2_O).

For the qualitative ^1^H-NMR analysis, around 100 mg of the powdered RYR samples were mixed with 1 mL of CD_3_CN:D_2_O (80:20 *v*/*v*) under vortex agitation for 1 min and then sonicated for 10 min. The suspension was then centrifuged (5 min, 3000 rpm) and 700 μL of the supernatant analyzed. TSP as internal chemical shift (δ) reference was added before NMR recording.

For the quantitative ^1^H-NMR analysis, between 20 and 100 mg of powdered sample was exactly weighed and mixed with 1 mL of CD_3_CN:D_2_O (80:20 *v*/*v*) under magnetic stirring during 20 min, then sonicated for 10 min. After centrifugation (5 min, 3000 rpm), 30 μL of a 10.0 mM solution of TSP were added to 800 μL of supernatant and the resulting solution was analyzed. The final concentration of TSP was 0.36 mM.

The efficacy of this single-step extraction procedure was demonstrated in samples with low and high contents of monacolins. Around 30, 85 and 90 mg accurately weighed of three different powdered samples (respectively DS **13**, **22** and **5**) were extracted as described above, except that after centrifugation, the whole supernatant was carefully collected and analyzed by ^1^H-NMR. The exactly weighed residual wet pellet was re-extracted with the same protocol than above and the supernatant analyzed using ^1^H-NMR. In the second extraction, the amounts of monacolins found were respectively ≈ 4.7%, 8.0% and 14.0% of that measured in the first extract. However, the solvent present in the wet pellet represented respectively at least (as the total weight of the powdered sample used was subtracted from the pellet weight) ≈ 5.4%, 8.3% and 14.0% of the initial solvent weight (1 mL = 905.48 mg). The amount of monacolins extracted was thus directly proportional to the amount of solvent remaining in the pellet. So, all the monacolins were dissolved in the solvent (1 mL) used in the single-step extraction procedure and were thus quantified.

For the quantitative analysis by UHPLC, between 10 and 100 mg of each powdered sample was exactly weighed and mixed with 1 mL of CH_3_CN:H_2_O (80:20 *v*/*v*) under magnetic stirring during 20 min, then sonicated for 10 min. The suspension was then centrifuged (5 min, 3000 rpm). The supernatant was filtered through a 0.45 µm pore size filter before the injection.

### 3.4. ^1^H-NMR Analysis

#### 3.4.1. Recording Conditions

For the qualitative analysis, the 1D ^1^H-NMR experiments were performed on a Bruker Avance 500 spectrometer (Bruker Biospin AG, Fallanden, Switzerland) equipped with a 5 mm ^1^H-optimized triple resonance NMR inverse helium-cooled probe (TCI CryoProbe) at 298K (standard ^1^H sensitivity for 0.1% ethylbenzene in CDCl_3_: SNR 4200). Typical acquisition parameters were as follows: spectral width 8000 Hz, 32K data points, pulse width 10.0 μs (flip angle 90°), acquisition time 2.04 s, relaxation delay 1 s, number of scans 16, corresponding to a recording time of 0.8 min.

For the quantitative 1D ^1^H-NMR experiments, analyses were acquired on a Bruker Avance 400 spectrometer equipped with a TBO (Triple resonance Broadband Observe) probe at 298K (standard ^1^H sensitivity for 0.1% ethylbenzene in CDCl_3_: SNR 266). The spectra were recorded with the following parameters: spectral width 8000 Hz, 64K data points, pulse width 3.7 μs (flip angle ≈ 30°), acquisition time 5.12 s, and relaxation delay 4.88 s. The number of scans was 64 or 512 for recording times of ≈11 or 85 min respectively. Three different samples of the same DS were analyzed.

T_1_ relaxation times of the methyl protons of TSP and those of the characteristic monacolin protons used for quantification (ethylenic protons of the hexahydronaphthalene ring (H6 and H4) and of the unsaturated lactone of dehydromonacolin derivatives (H22), and CH-OH proton of the lactone ring (H22)) (see Table 2 for proton numbering) were measured in RYR DS by the inversion-recovery pulse sequence method. Twenty delays from 0.001 to 30 s were used with an acquisition time of 2.56 sec and a relaxation delay of 30 s. The T_1_s found were between 1.6 and 2.2 s for monacolin derivatives and 4.5 s for TSP. All the ^1^H resonances were thus considered as fully relaxed since more than 98% of the signal intensity of the TSP protons was recovered in the recording conditions used.

The concentrations were calculated by comparing the signal areas of convenient protons of targeted compounds with those of TSP, the area of each NMR peak being directly proportional to the number of corresponding nuclei in fully relaxed recording conditions.

Data were processed with the Bruker TopSpin software 2.1 or 3.1 with one level of zero-filling and Fourier transformation after multiplying FIDs by an exponential line-broadening function of 0.3 Hz. Assignment of signals of MK, MKA, CP and DiMK was achieved with 1D ^13^C-NMR and 2D experiments (gCOSY, gHSQC and gHMBC).

#### 3.4.2. Quantification

The quantification was performed on the characteristic ^1^H-NMR signals (CH=, CH-OH and CH-OR) of the different monacolin families (monacolins in lactone and hydroxyl acid forms, dehydromonacolins and dihydromonacolins). The amount of compound (mg) per dosage unit is calculated from the following equation:

(1) $$Q=\frac{A_{i}}{A_{TSP}}\times \frac{Nb_{TSP}}{Nb_{i}}\times \;C_{TSP}\;\times \;\frac{V_{2}}{V_{1}}\;\times \;V_{t}\;\times \;M\;\times \;\frac{m_{t}}{m}$$

with *A_i_* and *A_TSP_* being the integrated areas of the characteristic NMR signal(s) of the monacolins to be quantified (*i*) and of the TSP signal respectively, *Nb_i_* and *Nb_TSP_* the number of protons contributing to the signal of the analyte and of TSP (9 protons), *C_TSP_* the concentration of TSP in the solution analyzed, *V*_1_ the volume of supernatant analyzed, *V*_2_ the volume of solution analyzed (*V*_1_ + *V_TSP_* (30 μL)), *V_t_* the volume used to dissolve the sample (1 mL), *M* the molecular weight of the analyte, *m_t_* and *m* the weights of the dosage unit (tablet or powder from capsule) and of the sample analyzed. As NMR did not allow to characterize individually all the monacolins present in RYR, the molecular weight considered was that of MK, the main one. This led to an approximation less than ±5% for MKA, DeMK, MX, DiMK and CP but higher for other monacolins, +10% for MN, +16% for MJA, ≈ +20% for MJ and MLA, +25% for ML and +29% for DeML (see Figure 2 for chemical structures). Nevertheless, all the monacolins whose molecular weight differs of more than 5% relative to MK, are very minor. From their amounts determined by UHPLC, we found that the approximation on their molecular weights led to an increase of the total content of monacolins determined by NMR of less than 3% (<1% for 22 DS, between 1 and 2.2% for 8 others, and 2.6% for one formulation).

### 3.5. UHPLC-DAD-MS Analysis

#### 3.5.1. Instrumentation

The liquid chromatographic system was a Waters Acquity UPLC-DAD-SQD (Ultra Performance Liquid Chromatography with Diode Array Detector and Single Quadrupole Detector). It consists of the following modular components: a binary pump, an automatic sample injector with two 48-well trays, a column oven, a diode array detector and a simple quadrupole detector with ESI. All the analyses were performed using ESI ionization with the following settings: positive mode, electrospray source temperature 135 °C, desolvatation temperature 300 °C, capillary voltage 2.8 kV, cone voltage 3 V, extractor voltage 2.0 V and RF lens voltage 0.1 V. The full scan mass spectra were acquired over a range of *m*/*z* 100–1000. The separations were achieved on a Kinetex C18 (100 mm × 2.1 mm, particle size 1.7 μm) column. The mobile phase consisted of water with 0.02% formic acid (solvent A) and acetonitrile with 0.02% formic acid (solvent B) at a flow rate of 0.6 mL min^−1^. The temperature of the column oven was set at 40 °C. The chromatographic analysis consisted of an isocratic elution with a 65% A/35% B mixture for 0.5 min followed by a linear gradient program: from 65% A/35% B to 35% A/65% B between 0.5 and 15 min and finally to 0% A/100% B over 3 min. After each run, the percentage of solvents ramped to their initial composition in 1 min and then the column was re-equilibrated for 2 min. The UV detection and quantification were performed at 238 nm and UV-Vis spectra were recorded within a range of 200–800 nm. The data acquisition and processing were done with the Empower 2 software.

#### 3.5.2. Validation Procedure

The UHPLC-UV method described was validated in terms of system suitability, linearity, precision, sensitivity (LOD, LOQ) and specificity.

The system suitability tests to ensure the reproducibility of the chromatographic system were performed by injecting six times 1 µL of a solution of standard lovastatin. The RSD found was 0.2% for a 0.42 mg mL^−1^ solution and 1.5% for a 4.2 × 10^−3^ mg mL^−1^ solution and was acceptable as it was less than 2% [13].

The linearity of the UHPLC-UV assay was tested for eight concentration levels of standard lovastatin in the range 4.18 × 10^−3^–0.42 mg mL^−1^ in CH_3_CN:H_2_O (80:20 *v*/*v*). The correlation coefficient value (r² = 0.9993) of the calibration curve obtained by plotting the peak areas versus concentrations indicated satisfactory linearity of the method in the range studied. All the monacolins with a characteristic UV maximum absorption peak at ≈238 nm were quantified using the calibration curve established for standard lovastatin and their amounts in mg calculated considering their respective molecular weights. DiMK was not detected at this wavelength and was thus quantified by MS from its [MH]^+^ peak area by comparison to that of MK whose amount was previously determined by UV, considering that their ionization efficiencies were similar.

Each solution of extracted RYR sample was analyzed three times. Two independently prepared samples were analyzed for 18 RYR DS and three for the other 13.

The specificity of the method, under the conditions described above, was verified using the chromatographic peak purity tool included in the Empower 2 software and showed no co-elution between peaks of monacolins and those of the complex matrix.

### 3.6. High-Resolution Mass Spectrometry (HR-MS) Experiments

HR-MS and HR-MS/MS were performed on a Waters Xevo G2 QTOF mass spectrometer (Waters, Manchester, UK) with ESI in positive mode (ESI^+^) and in few cases in negative mode (ESI^−^). For both modes, the instrument parameters were as follows: for MS analysis, cone voltage 30 or 50 V, source temperature 110 °C, desolvatation temperature 300 °C, cone gas flow rate 20 L h^−1^, scan range *m*/*z* 50–1200; for MS/MS analysis, three collision energies were used 15, 25 and 35 V with the cone voltage maintained at 30 V or 50 V and the spectra were acquired with a mass precursor ion selection of 3 Da. All analyses were performed using the lockspray, which ensured accuracy and reproducibility. Leucine enkephalin (1 ng μL^−1^) introduced by a lockspray at 3 μL min^−1^ was used as the lockmass generating reference ions at *m*/*z* 556.2771 or 554.2615 in positive or negative mode respectively. The MassLynx software was used to calculate the most probable chemical formula and the theoretical exact mass of the molecular and fragment ions by comparison with their measured accurate ionic masses.

### 3.7. Statistical Analysis

Comparison of quantitative determination of the different monacolin sets obtained by ^1^H-NMR and UHPLC was performed (i) by simple linear regression and (ii) with the Wilcoxon signed-rank test, a non-parametric test that can be used as an alternative to the paired Student’s t-test for matched pairs when the population cannot be assessed to be normally distributed; the *p*-values > 0.05 (95% confidence level) were considered as proofs of no significant difference between the values measured by the two methods.

## 4. Conclusions

This work demonstrated that ^1^H-NMR is a powerful method to establish rapidly (<1 min) the spectral signature of RYR DS and to afford the quantitative determination of their total monacolin content in a reasonable recording time (from ≈10 to 90 min depending on their concentration in the solution analyzed). The method was validated against UHPLC-DAD-MS, the gold standard technique for a detailed determination of all the monacolins existing in RYR products. Indeed, the quantification of the ^1^H resonances of the hexahydronaphthalene ring at 5.84 and 5.56 ppm, characteristic of all the monacolin families except the dihydomonacolins present in very low amounts, led to values in close agreement with those of all the monacolins, including dihydromonacolins, measured by UHPLC. These two resonances did not overlap with matrix signals in the RYR DS tested and their quantification is only limited by the sensitivity of the method (LOQ ≈ 0.25 mg per capsule or tablet in our recording conditions). Therefore, ^1^H-NMR can be considered as an accurate method for determining the total monacolin content of RYR DS, not only those of the main ingredients MK and MKA, but also of minor monacolins which are also active HMG-CoA reductase inhibitors although generally less potent, accounting for a non-negligible amount (mean of ≈ 18%) of TotalM. Taking into account the easy preparation of the RYR DS for the analysis (a simple extraction of capsule/tablet powder by an adequate deuterated solvent), ^1^H-NMR can be proposed as a high-throughput (thanks to commonly used sample changers) screening method for quality control of RYR formulations on the market. Indeed, the product labels are often incomplete and inappropriate: 42% of the RYR DS analyzed in this study did not provide information on the concentration of monacolins; when the label mentioned MK, it was not specified whether the amount of MK included or not MKA; the amount of the other minor monacolins was never indicated. The deviation between labelled and measured contents must also be emphasized: only 50% of the labelled formulations actually contained the declared amount of monacolin(s). In conclusion, health authorities should impose to manufacturers a strict control of the quality of the RYR DS to assess the ability of the marketed RYR products in reducing/regulating cholesterol level.

## Figures and Tables

**Table 1:** Red Yeast Rice (RYR) dietary supplements investigated in this study.

Number	Formulation Name (Origin ^1^)	Batch Number Expiration Date		Form	RYR Extract Content Per Capsule or Tablet on the Label (mg)
1	Arkopharma (HFS)	C02194A	04/2016	Capsule	175
2	Arterin (I)	1983-1	07/2016	Tablet	670
3	B.concept (I)	13DN8	09/2016	Capsule	186
4	Belle & Bio (I)	B524A	06/2015	Capsule	250
5	Blue bonnet (I)	31201702	01/2017	Capsule	600
6	Boutique nature (I)	32443A	03/2016	Capsule	222.3
7	Doctor’s best (I)	ML847	01/2017	Tablet	1200
8	Ephyto (I)	B1204059CZ	01/2015	Tablet	600
9	Fushi (I)	EPAN110412	08/2015	Capsule	600
10	Hanoju (I)	20130612	06/2016	Capsule	450
11	Health Ace (I)	106012W9	03/2015	Capsule	650
12	Health Spark (I)	1060012/A1	10/2014	Capsule	650
13	Liposterol (HFS)	03023	05/2016	Tablet	600
14	MRM (I)	130736	08/2016	Capsule	600
15	Nat et form (I)	14387J	10/2016	Capsule	600
16	Natrol (I)	2055200	10/2015	Tablet	400
17	Nature algues (I)	G0112112602 PF01G	11/2016	Capsule	250
18	Nature’s plus (I)	1249540	09/2016	Tablet	600
19	Nature’s way (I)	20012472	05/2016	Capsule	600
20	Naturland (HFS)	C02196A	04/2016	Capsule	175
21	Now (I)	16117750521	06/2015	Capsule	600
22	Nutrisanté (I)	961PAA	05/2016	Capsule	222.2
23	Pharma nature (HFS)	1009.211	12/2014	Capsule	300
24	Phytalessence (HFS)	10134	10/2016	Tablet	600
25	Phytoreponse (I)	B1210229CZ	10/2015	Tablet	600
26	Rizocol (I)	9545	06/2016	Tablet	335
27	Santé verte (HFS)	L066X2	03/2016	Tablet	600
28	Solaray (I)	171306	12/2016	Capsule	600
29	Solgar (I)	747369-02	07/2016	Capsule	600
30	Tradition soleil levant (I)	89853	07/2016	Capsule	208
31	Vit’All + (HFS)	A7248-11	11/2013	Tablet	600

^1^ Dietary supplements bought on internet web sites (I) or in health food stores (HFS).

**Table 3:** ^1^H-NMR analysis of RYR dietary supplements: quantitative determination of monacolins (mg per capsule or tablet) ^1^ and list of other compounds identified.

DS Number	Signal at 5.84 ppm ^2^ H6	Signal at 5.56 ppm ^2^ H4	Mean Intensity of Signals H6 and H4	Signal at 5.33 ppm ^2^ H1	Signal at 4.60 ppm ^2^ H20	Signal at 4.25 ppm ^2^ H22	Compounds Identified Other than Monacolins ^3,4^
1	1.67	1.70	1.685	3.31	1.40	1.34	SFA *, UFA, glycerol, glucose, monascin
2	9.14	9.18	9.16	9.26		7.10	SFA *, UFA, glycerol, glucose
3	3.07	3.07	3.07	2.39			SFA *, UFA, carnitine *, vitamin B3 *, vitamin C *
4	1.02	0.95	0.985	1.64		0.71	SFA, UFA, glucose
5	2.47	2.53	2.50	11.3	2.15		SFA *, UFA, glycerol, glucose, monascin
6	2.92	2.63	2.775	3.27		2.19	SFA *, UFA, glucose, monascin
7	3.76	3.73	3.745				SFA *, UFA, glucose, piperine *
8	8.68	8.79	8.735	8.65	6.89	7.73	SFA, UFA, glycerol, monascin
9	5.14	5.21	5.125	8.03	4.55	4.55	SFA *, UFA, glycerol, glucose, monascin
10	2.10	2.04	2.07	8.67		1.22	SFA, UFA, glycerol, glucose, monascin
11							SFA, UFA, glycerol, glucose, monascin
12							SFA, UFA, glycerol, glucose, monascin
13	23.9	23.9	23.9	21.9	23.9	23.2	SFA *, UFA, glycerol, glucose
14	3.45	3.28	3.365	10.8		2.26	SFA, UFA, glycerol, glucose, monascin
15	11.9	12.3	12.1		9.85		SFA *, UFA, glycerol, monascin
16		0.31					SFA *, UFA, glycerol, glucose, monascin, piperine *
17	1.47	1.51	1.49	4.06	1.28		SFA, UFA, glycerol, monascin
18	8.84	8.19	8.515		6.68	7.04	SFA *, UFA, glycerol, glucose, monascin
19	0.36	0.44	0.40				SFA *, UFA, glycerol, glucose, monascin
20	1.75	1.77	1.76	3.51	1.27	1.49	SFA *, UFA, glycerol, glucose, monascin
21	0.48	0.47	0.475				SFA *, UFA, glycerol
22	3.09	3.02	3.055			2.26	SFA *, UFA, glycerol, glucose, monascin
23	10.6	10.7	10.65	10.0	10.0	9.77	SFA *, UFA, glycerol, glucose, monascin
24	11.5	11.8	11.65	9.38		5.97	SFA *, UFA, glucose, sorbitol *
25	10.0	9.72	9.87		5.97	7.26	SFA, UFA, glycerol, monascin
26	4.51	4.54	4.525			3.88	SFA *, UFA, glycerol, glucose, sorbitol
27	4.13	3.84	3.985		2.97	2.90	SFA, UFA, glycerol, glucose, monascin, chlorogenic acid *
28	1.64	1.75	1.695		1.05	1.31	SFA *, UFA, glycerol, glucose
29							SFA *, UFA, glycerol, glucose
30	2.99	3.04	3.015	2.61		2.43	SFA, UFA
31	1.21	1.28	1.245	2.16			SFA *, UFA, glycerol, glucose, monascin, isopropanol

^1^ The amounts of monacolins (mg per dosage unit) were calculated from the measured areas using the equation presented in the paragraph 3.4.2. ^2^ The resonances at 5.84, 5.56 ppm and 5.33 ppm are characteristic of all the monacolins bearing an hexahydronaphthalene ring but the 5.33 ppm signal does not characterize MJ and ML in lactone or in hydroxyl acid form; the resonances at 4.60 and 4.25 ppm are specific of all the monacolins in lactone form including dihydromonacolins. ^3^ SFA: saturated fatty acids; UFA: non-conjugated unsaturated fatty acids; monascin corresponds to monascin and other pigments with the same skeleton (see Table 2 and Figure 2 for the chemical structures of all these compounds). ^4^ The asterisk * means that the compound was mentioned on the label of the formulation.

**Table 4:** Retention times, UV-Vis characteristics and accurate mass measurements of the compounds observed and identified from the RYR dietary supplements analyzed with UHPLC.

Compound Name	Retention Time (min)	UV-Vis λ_max_ (nm)	Formula (Monoisotopic Mass)	Calculated Mass (*m*/*z*)		Measured Mass (*m*/*z*)	Mass Error (mDa)	Relative Mass Error (ppm)	Major ions of [M + H]^+^ MS/MS Fragmentation Unless Otherwise Indicated (*m*/*z*)
Monacolin J hydroxyl acid (MJA)	0.90	230, 239, 244	C_19_H_30_O_5_ (338.2172)	[M − H]^−^	337.2015	337.2010	−0.5	−1.5	Not recorded
Citrinin	1.45	243, 326 ^1^	C_13_H_14_O_5_ (250.0841)	[M + H]^+^	251.0919	251.0922	+0.3	+1.2	Not recorded
Monacolin J (MJ)	1.98	230, 239, 246	C_19_H_28_O_4_ (320.1988)	[M + H]^+^ [M + Na]^+^ [M − H]^−^	321.2066 343.1885 319.1909	321.2067 343.1881 319.1910	+0.1 −0.4 +0.1	+0.3 −1.2 +0.3	303.1945, 285.1847, 267.1711, 205.1604, 199.1451, 159.1162
Rubropunctamine	2.70	252, 303, 415, 525	C_21_H_23_NO_4_ (353.1627)	[M + H]^+^ [M + Na]^+^ [M − H]^−^	354.1705 376.1525 352.1549	354.1710 376.1521 352.1551	+0.5 −0.4 +0.2	+1.4 −1.1 +0.6	337.1308, 294.0762, 267.0523, 256.0866, 239.0580
Monacolin N (MN)	3.89	230, 239, 247	C_21_H_30_O_5_ (362.2093)	[M + H]^+^ [M + Na]^+^	363.2171 385.1991	363.2159 385.1988	−1.2 −0.3	−3.3 −0.8	345.2052, 285.1865, 267.1750, 243.1711, 199.1490, 173.1335, 159.1173, 143.0856
Monacolin L hydroxyl acid (MLA)	4.05	230, 239, 247	C_19_H_30_O_4_ (322.2144)	[M + H]^+^ [M + Na]^+^ [M − H]^−^	323.2222 345.2042 321.2066	323.2220 345.2037 321.2072	−0.2 −0.5 +0.6	−0.6 −1.5 +1.9	305.2126, 287.1986, 269.1901, 225.1640, 203.1800, 159.1172
Monacolin X (MX)	4.38	230, 239, 246	C_24_H_34_O_6_ (418.2355)	[M + H]^+^ [M + Na]^+^	419.2434 441.2253	419.2434 441.2250	0 −0.3	0 −0.7	303.1961, 285.1855, 267.1749, 243.1751, 225.1643, 199.1489, 173.1331, 159.1173, 143.0712
7-(2-hydroxyethyl)-monascorubramine (PP-R)	4.66	251, 302, 423, 530	C_25_H_31_NO_5_ (425.2202)	[M + H]^+^ [M + Na]^+^	426.2280 448.2100	426.2265 448.2092	−1.5 −0.8	−3.5 −1.8	Not recorded
Monascorubramine	5.55	251, 307, 412, 530	C_23_H_27_NO_4_ (381.1940)	[M + H]^+^ [M + Na]^+^ [M − H]^−^	382.2018 404.1838 380.1862	382.2018 404.1842 380.1867	0 +0.4 +0.5	0 +1.0 +1.3	365.1624, 294.0765, 267.0528, 250.0870, 239.0582
Monacolin K hydroxyl acid (MKA)	5.64	229, 238, 246	C_24_H_38_O_6_ (422.2669)	[M + H]^+^ [M + Na]^+^ [M − H]^−^ [M – H + CO]^−^	423.2747 445.2566 421.2590 449.2539	ND^2^ 445.2561 421.2589 449.2540	−0.5 −0.1 +0.1	−1.1 −0.2 +0.2	MS/MS [M − H]^−^ 319.1902, 101.0602, 85.0286
Monascuspiloin (dihydromonascin)	5.92	233, 291, 390	C_21_H_28_O_5_ (360.1937)	[M + H]^+^ [M + Na]^+^	361.2015 383.1834	361.2009 383.1836	−0.6 +0.2	−1.7 +0.5	345.2045, 300.2883, 261.1133, 215.1081, 187.1134
Monacolin L (ML)	6.09	230, 238, 246	C_19_H_28_O_3_ (304.2039)	[M + H]^+^ [M + Na]^+^	305.2117 327.1936	305.2116 327.1935	−0.1 −0.1	−0.3 −0.3	287.2004, 269.1902, 251.1179, 225.1649, 203.1797, 201.1643, 199.1488, 173.1333, 159.1174, 145.1015
Compactin (CP) (mevastatin)	6.18	230, 238, 246	C_23_H_34_O_5_ (390.2406)	[M + H]^+^ [M + Na]^+^	391.2484 413.2304	391.2488 413.2305	+0.4 +0.1	+1.0 +0.2	289.1793, 271.1693, 253.1588, 229.1587, 211.1481, 185.1324, 159.1168
Monasfluore A	6.28	379 ^3^	C_21_H_24_O_5_ (356.1624)	[M + H]^+^ [M + Na]^+^	357.1702 379.1521	357.1701 379.1522	−0.1 +0.1	−0.3 +0.3	Not recorded
Monaphilone B	7.23	227, 285, 386	C_20_H_28_O_4_ (332.1988)	[M + H]^+^ [M + Na]^+^	333.2066 355.1885	333.2062 355.1880	−0.4 −0.5	−1.2 −1.4	287.2012, 217.1234, 201.0911, 189.1385, 173.0970
Monacolin K (MK) (lovastatin)	7.79	229, 238, 246	C_24_H_36_O_5_ (404.2563)	[M + H]^+^ [M + Na]^+^ [2M + Na]^+^	405.2641 427.2460 831.5023	405.2642 427.2461 831.5026	+0.1 +0.1 +0.3	+0.3 +0.3 +0.4	303.1995, 285.1855, 267.1753, 243.1751, 225.1652, 199.1490, 173.1328, 159.1176, 143.0854
Monascin	8.19	232, 291, 392	C_21_H_26_O_5_ (358.1781)	[M + H]^+^ [M + Na]^+^	359.1859 381.1678	359.1854 381.1684	−0.5 +0.6	−1.4 +1.6	343.2274, 315.2317, 261.1112, 215.1073, 187.1121
Dihydromonacolin K (DiMK)	9.78	ND ^4^	C_24_H_38_O_5_ (406.2719)	[M + H]^+^ [M + Na]^+^	407.2798 429.2617	407.2796 429.2618	−0.2 +0.1	−0.5 +0.2	305.2115, 287.2015, 269.1899, 227.1797, 203.1802
Monasfluore B	9.97	378 ^3^	C_23_H_28_O_5_ (384.1937)	[M + H]^+^ [M + Na]^+^	385.2015 407.1835	ND ^2^ 407.1839	+0.4	+1.0	Not recorded
Dehydromonacolin L (DeML)	10.15	230, 238, 246	C_19_H_26_O_2_ (286.1933)	[M + H]^+^ [M + Na]^+^	287.2011 309.1830	287.2007 ND ^2^	−0.4	−1.4	269.1896, 225.1636, 201.1645, 199.1483, 173.1329, 159.1171, 145.1013
Monaphilone A	11.15	230, 289, 389	C_22_H_32_O_4_ (360.2301)	[M + H]^+^ [M + Na]^+^	361.2379 383.2198	361.2380 ND ^2^	+0.1	+0.3	287.2020, 217.1322, 189.1390, 173.0952
Dehydromonacolin K (DeMK)	11.42	230, 238, 246	C_24_H_34_O_4_ (386.2457)	[M + H]^+^ [M + Na]^+^ [2M + Na]^+^	387.2535 409.2355 795.4812	387.2532 409.2356 795.4821	−0.3 +0.1 +0.9	−0.8 +0.3 +1.1	345.2037, 285.1851, 267.1747, 249.1625, 199.1484, 173.1325, 159.1167, 143.0858
Ankaflavin	11.75	232, 290, 391	C_23_H_30_O_5_ (386.2093)	[M + H]^+^ [M + Na]^+^	387.2172 409.1991	387.2173 409.1994	+0.1 +0.3	+0.3 +0.7	359.2238, 315.2325, 261.1117, 215.1076, 187.1125

^1^ The UV absorption bands at 243 and 326 nm (low broad) match well with the UV profile reported in the literature for UHPLC-DAD analytical conditions similar to those used in our study (λ_max_ at 240, 282 (shoulder) and 333 (low broad) nm) [35]. ^2^ ND: not detected. ^3^ This low and broad UV absorption band is in agreement with the mean of the λ_max_ of the two enlarged and poorly separated peaks at 371 and 386 nm reported in the literature [36]. ^4^ ND: not detected at the wavelength used in this study (238 nm).

**Table 5:** Chemical fingerprints of the RYR dietary supplements analyzed by UHPLC and quantitative determination of the monacolins identified (mg per capsule or tablet).

DS	Monacolins ^1^												Total M ^2^	MK+MKA (%)	MK/MKA	%DiMK/TotalM	Azaphilones ^3^
	MJA	MJ	MN	MLA	MX	MKA	ML	CP	MK	DiMK	DeML	DeMK					
1	-	-	-	-	-	0.49	-	-	1.33	-	-	0.02	1.84	1.82 (98.9)	2.7	-	Mocusp, Mop B, Mo
2	-	-	-	0.04	-	0.82	0.20	0.03	6.77	0.21	0.05	0.68	8.80	7.59 (86.3)	8.3	2.4	
3	-	-	-	-	-	0.23	0.04	0.01	2.16	0.04	0.01	0.13	2.62	2.39 (91.2)	9.4	1.5	
4	-	0.01	0.02	0.01	-	0.10	0.04	0.03	0.48	0.11	0.02	0.29	1.11	0.58 (52.3)	4.8	9.9	Mocusp
5	-	0.01	0.02	0.02	-	0.50	0.05	0.04	1.80	0.20	0.02	0.38	3.04	2.30 (75.7)	3.6	6.6	Rubro, Moco, Mocusp, Mop B, Mo, Mof B, Mop A, Anka
6	-	-	-	0.03	-	0.31	0.08	0.03	2.05	0.11	0.03	0.27	2.91	2.36 (81.1)	6.6	3.8	Mop B
7	-	0.06	-	0.06	-	0.37	0.19	0.08	2.22	0.20	0.06	0.85	4.09	2.59 (63.3)	6.0	4.9	
8	-	0.02	0.09	0.12	-	2.13	0.18	0.28	6.26	0.59	-	0.16	9.83	8.39 (85.4)	2.9	6.0	Cit, Rubro, Mocusp, Mof A, Mop B, Mo
9	-	-	-	-	-	0.13	-	-	4.17	-	-	0.28	4.58	4.30 (93.9)	32.1	-	Mo, Anka
10	-	0.02	-	0.01	-	0.96	0.02	0.04	0.85	0.10	0.01	0.31	2.32	1.81 (78.0)	0.9	4.3	Cit, Rubro, Mocusp, Mop B
11	-	-	-	-	-	-	-	-	0.07	-	-	-	0.07	0.07 (100)	-	-	Rubro, Moco, Mop B, Mo, Mop A, Anka
12	-	-	-	-	-	-	-	-	0.08	-	-	-	0.08	0.08 (100)	-	-	Rubro, PP-R, Moco, Mop B, Mo, Mof B, Mop A, Anka
13	-	-	-	-	-	0.62	-	-	23.18	-	-	0.04	23.84	23.80 (99.8)	37.4	-	
14	0.03	0.05	0.07	0.04	-	0.27	0.18	0.08	2.19	0.23	0.06	0.56	3.76	2.46 (65.4)	8.1	6.1	Mocusp, Mop B, Mo
15	-	-	-	0.19	-	1.33	0.86	0.58	8.27	1.16	0.16	1.44	13.99	9.60 (68.6)	6.2	8.3	Rubro, Mocusp, Mof A, Mop B, Mo
16	-	-	-	-	-	-	0.02	0.02	0.33	0.01	-	0.03	0.41	0.33 (80.5)	-	2.4	Rubro, PP-R, Moco, Mop B, Mo, Mof B, Mop A, Anka
17	-	-	-	0.03	-	0.16	0.12	0.16	1.09	0.13	0.02	0.15	1.86	1.25 (67.2)	6.8	7.0	Mocusp, Mof A, Mop B, Mo
18	-	-	-	-	-	0.98	-	-	6.44	0.13	-	0.38	7.93	7.42 (93.6)	6.6	1.6	Mocusp, Mop B, Mo
19	-	0.01	-	0.01	-	0.05	0.03	0.01	0.25	0.02	0.01	0.11	0.50	0.30 (60.0)	5.0	4.0	
20	-	-	-	-	-	0.48	-	-	1.29	-	-	0.02	1.79	1.77 (98.9)	2.7	-	Mocusp, Mop B, Mo
21	-	0.01	0.01	0.01	-	0.07	0.02	-	0.24	0.02	0.01	0.12	0.51	0.31 (60.8)	3.4	3.9	
22	-	-	-	0.02	-	0.68	0.03	0.08	1.77	0.17	0.01	0.37	3.13	2.45 (78.3)	2.6	5.4	Rubro, Mocusp, Mop B, Mo
23	-	-	-	0.04	0.01	0.68	0.05	0.10	8.47	0.17	0.03	0.23	9.78	9.15 (93.6)	12.5	1.7	Rubro, Mocusp, Mop B, Mo
24	-	-	-	-	-	5.50	-	-	5.21	-	-	0.07	10.78	10.71 (99.4)	0.95	-	
25	-	0.09	0.10	0.11	0.04	3.56	0.14	0.29	5.69	0.67	0.03	0.56	11.28	9.25 (82.0)	1.6	5.9	Rubro, Mocusp, Mof A, Mop B, Mo
26	-	-	-	0.03_5_	-	0.39	0.10	0.04	3.52	0.15	0.03_5_	0.48	4.75	3.91 (82.3)	9.0	3.2	Mocusp
27	-	-	-	-	-	0.61	-	-	2.68	-	-	0.09	3.38	3.29 (97.3)	4.4	-	Mop B, Mo
28	-	0.03	0.04	0.04	-	0.41	0.07	0.05	0.98	0.15	0.02	0.32	2.11	1.39 (65.9)	2.4	7.1	Mop B
29	-	-	-	-	-	0.01	-	-	0.08	-	-	0.04	0.13	0.09 (69.2)	8.0	-	
30	-	0.02	0.03	0.02	-	0.37	0.05	0.02	2.19	0.07	0.02	0.30	3.09	2.56 (82.8)	5.9	2.3	Mop B
31	-	-	-	-	-	0.31	0.04	0.03	0.85	0.07	0.01	0.12	1.43	1.16 (81.1)	2.7	4.9	Rubro, Mop B, Mo, Anka

^1^ Monacolins are listed in increasing order of elution times. MJ, MN, MX, ML, MK: monacolins J, N, X, L, K; MJA, MLA, MKA: monacolins J, L, K in hydroxyl acid forms; CP: compactin; DiMK: dihydromonacolin K; DeML, DeMK: dehydromonacolins L,K. ^2^ TotalM: total monacolins. ^3^ Azaphilones are listed in increasing order of elution times. Anka: ankaflavin; Cit: citrinin; Mo: monascin; Moco: monascorubramine; Mocusp: monascuspiloin; Mof A, Mof B: monasfluore A, monasfluore B; Mop A, Mop B: monaphilone A, monaphilone B; PP-R: 7-(2-hydroxyethyl)-monascorubramine; Rubro: rubropunctamine.

**Table 6:** Comparison of the percentages of MK, MK + MKA and total monacolins per pill to monacolin labelling; amounts of MK, MK + MKA and total monacolins per recommended daily serving.

N°	Amount of Monacolin(s) Claimed Per Pill (mg) ^1^	% Measured/Claimed Per Pill				Serving(s) Per Day ^4^	MK Amount (mg/day) ^5^	MK + MKA Amount (mg/day) ^5^	Total Monacolins (mg/day) ^5^	
		MK ^2^	MK + MKA ^2^	Total Monacolins ^3^					UHPLC ^3^	NMR ^3^
				UHPLC	NMR					
1	MK 2.6	51	70	71	65	1	1.3	1.8	1.8	1.7
2	MK + MKA 10.05	68	76	88	91	1	6.8	7.6	8.8	9.2
3	MK 2.8	77	85	94	110	3	6.5	7.2	7.9	9.2
4	*M. purpureus* 1.0 ^6^	48	58	109	99	3	1.4	1.7	3.3	3.0
5						1	1.8	2.3	3.0	2.5
6	MK 3.33	62	71	87	83	3	6.2	7.1	8.7	8.3
7						1	2.2	2.6	4.1	3.8
8						2	12.5	16.8	19.7	17.5
9						2	8.3	8.6	9.2	10.3
10	MK 9	9	20	26	23	4	3.4	7.2	9.3	8.3
11	MK 2.6	3	3	3		4	0.3	0.3	0.3	
12	MK 2.6	3	3	3		1	0.08	0.08	0.08	
13	MK 24.0	97	99	99	100	2	46.2	47.6	47.7	47.8
14						2	4.4	4.9	7.5	6.7
15	MK 9.6	86	100	146	126	1	8.3	9.6	14.0	12.1
16						2	0.7	0.7	0.8	0.6
17						2–3	2.2–3.3	2.5–3.8	3.7–5.6	3.0–4.5
18						1	6.4	7.4	7.9	8.5
19						2–4	0.5–1.0	0.6–1.2	1.0–2.0	0.8–1.6
20	MK 2.6	50	68	69	68	1	1.8	1.8	1.8	1.7
21						2–4	0.5–1.0	0.6–1.2	1.0–2.0	0.95–1.9
22	MK 2.33	76	105	134	131	3	5.3	7.4	9.4	9.2
23	MK 9	94	102	109	118	1	8.5	9.2	9.8	10.6
24	MK 10.0	52	107	108	117	1	5.2	10.7	10.8	11.7
25	Monacolin 9.6	59	96	118	103	1	5.7	9.3	11.3	9.9
26	MK 5.0	70	78	95	91	1–2	3.5–7.0	3.9–7.8	4.8–9.5	4.5–9.1
27	MK 2.6	103	126	130	153	1	2.7	3.3	3.4	4.0
28						1	1.0	1.4	2.1	1.7
29						2–4	0.2–0.3	0.2–0.4	0.25–0.5	
30	Monacolin 3.3	66	77	93	91	3	6.6	7.7	9.3	9.0
31						1	0.9	1.2	1.4	1.2

^1^ The exact monacolin labelling is indicated for each formulation. ^2^ Percentages of MK and MK + MKA were calculated from amounts determined by UHPLC-DAD-MS (see Table 5). ^3^ Percentages of total monacolins determined by UHPLC-DAD-MS and ^1^H-NMR correspond, respectively, to TotalM and TotalM-DiMK (see Table 3 and Table 5). ^4^ Serving(s) recommended per day on the label. ^5^ Amounts of MK, MK + MKA and total monacolins per recommended daily serving. ^6^ The exact label is: 750 mg of organic RYR titrated at 0.4% of *Monascus purpureus* for three pills.

## Supplementary Materials

The following are available online, Table S1: ^1^H and ^13^C-NMR data (solvent: CD_3_CN:D_2_O 80:20) of monacolin K in lactone (MK) and hydroxyl acid (MKA) forms, compactin and dihydromonacolin K. Table S2: ^1^H-NMR quantitative determination of monacolins in RYR dietary supplements. Table S3: Comparison of monacolin amounts measured in RYR dietary supplements by ^1^H-NMR and UHPLC. Figure S1: ^1^H-NMR spectra (CD_3_CN:D_2_O (80:20)) of all the dietary supplements analyzed in the present study. Upper part: entire spectrum, lower part: enlarged downfield region (4–9 ppm). Figure S2: UHPLC chromatograms with UV detection at 238 nm (A) and full scan MS profile in positive ESI mode (B) of 9 RYR dietary supplements.
