# Supplementary material for: Comparative Chemical Profiling and Monacolins Quantification in Red Yeast Rice Dietary Supplements by 1H-NMR and UHPLC-DAD-MS
Source: Molecules. 2020 Jan 13;25(2):317. doi: 10.3390/molecules25020317 (PMC7024183; doi:10.3390/molecules25020317)
Supplement: Supplementary file 1 [file molecules-25-00317-s001.pdf]

SUPPORTING INFORMATION

Comparative chemical profiling and monacolins quantification in red yeast rice dietary supplements by ^{1}H NMR and UHPLC-DAD-MS

Rabab Hachem[1], Gaëtan Assemat[1], Stéphane Balayssac[1], Nathalie Martins-Froment[2], Véronique Gilard[1], Robert Martino[1], Myriam Malet-Martino[1,*]

[1] Biomedical NMR Group, Laboratoire SPCMIB, UMR CNRS 5068, Université Paul Sabatier, 118 route de Narbonne, 31062 Toulouse Cedex 9, France; Rabab.Hachem@evotec.com (R.H.); gaetan-ass@hotmail.fr (G.A.); balayssac@chimie.ups-tlse.fr (S.B.); gilard@chimie.ups-tlse.fr (V.G.); rmartino@chimie.ups-tlse.fr (R.M.); martino@chimie.ups-tlse.fr (M.M.-M.)

[2] Service commun de spectrométrie de masse, Institut de Chimie de Toulouse, Université Paul Sabatier, 118 route de Narbonne, 31062 Toulouse Cedex 9, France; martins@chimie.ups-tlse.fr (N.M.-F.)

* Correspondence: martino@chimie.ups-tlse.fr

Table S1. ^{1}H and ^{13}C NMR data (solvent: $CD_3CN:D_2O$ 80:20) of monacolin K in lactone (MK) and hydroxyl acid (MKA) forms, compactin and dihydromonacolin K.

Monacolin K (MK)

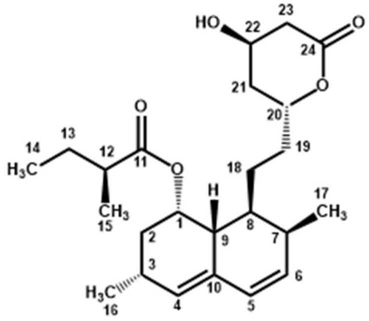

Positions		δ ^{1}H (ppm)	Multiplicity[1] (J (Hz))	COSY ^{1}H-^{1}H correlations $^{2}J_{HH}$	$^{3}J_{HH}$	$^{4}J_{HH}$	δ ^{13}C (ppm)	HMBC ^{1}H-^{13}C correlations $^{2}J_{CH}$	$^{3}J_{CH}$
5	CH	6.01	d (9.6)	-	H6	H7	131.0	-	H4/H7
6	CH	5.84	dd (6.1, 9.6)	-	H5/H7	H17	136.4	H7	H17
4	CH	5.56	app t (2.8)	-	H3	H2/H9/H16	132.4	-	H2/H9/H5/H16
1	CH	5.35	q (3.2)	-	H2/H9	-	71.2	H2	-
20	CH	4.59	m	-	H19/H21	-	79.7	H19/H21	-
22	CH	4.25	app quint (3.9)	-	H21/H23	-	64.6	H21/H23	-
23	CH_2	2.69	Ad (4.9, 17.6)	H23	H22	-	40.8	-	-
		2.51	Bdd (1.7, 3.8, 17.6)	H23	H22	H21			
3	CH	2.45	m	-	H2/H4/H16	-	30.1	H2/H4/H16	H1
7	CH	2.42	m	-	H6/H8/H17	H5	33.3	H6	H5/H18
9	CH	2.37	m	-	H8/H1	H4	39.7	H1	H2/H4/H5/H7/H18
12	CH	2.35	m	-	H13/H15	-	44.3	H13/H15	H14
2	CH_2	1.96	m	-	H3/H1	-	35.3	-	H4/H16
21	CH_2	1.90	m	H21	H20/H22	-	37.9	-	H19/H23
		1.71	m	H21	H20/H22	H23		-	
8	CH	1.69	m	-	H9/H7/H18	-	39.4	H9/H7/H18	H6
13	CH_2	1.62	app qd (7.4, 13.6)	H13	H12/H14	-	29.5	H12/H14	H15
		1.46	m	H13	H12/H14	-			
19	CH_2	1.81	m	H19	H18/H20	-	35.0	H18	-
		1.37	m	H19	H18/H20	-			
18	CH_2	1.46	m	-	H8/H19	-	26.5	H19	-
		1.36	m	-	H8/H19	-			
15	CH_3	1.08	d (6.9)	-	H12	-	18.5	H12	H13
16	CH_3	1.06	d (7.4)	-	H3	-	25.0	-	H2
17	CH_3	0.89	d (6.9)	-	H7	-	15.9	H7	-
14	CH_3	0.88	t (7.5)	-	H13	-	14.0	H13	H12
10	Cq	-	-	-	-	-	134.7	H9/H5	H6/H1
24	Cq	-	-	-	-	-	175.1	H23	-
11	Cq	-	-	-	-	-	180.1	H12	H13/H15/H1

Monacolin K hydroxyl acid form (MKA)

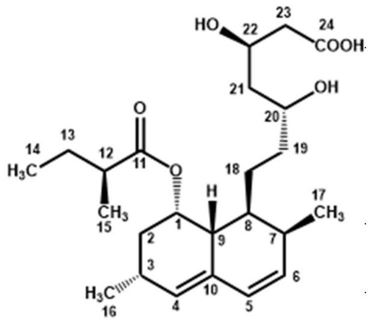

Positions		δ ^{1}H (ppm)	Multiplicity[1] (J (Hz))	COSY ^{1}H-^{1}H correlations			δ ^{13}C (ppm)	HMBC ^{1}H-^{13}C correlations	
				$^2J_{HH}$	$^3J_{HH}$	$^4J_{HH}$		$^2J_{CH}$	$^3J_{CH}$
5	CH	5.99	d (9.6)	-	H6	H7	130.9	-	H4/H7
6	CH	5.83	dd (6.1, 9.6)	-	H5/H7	H17	136.9	H7	H17
4	CH	5.53	app t (2.8)	-	H3	H2/H9/H16	132.2	-	H2/H9/H5/H16
1	CH	5.33	q (3.2)	-	H2/H9	-	71.6	H2	-
20	CH	3.63	app hept (4.1)	-	H19/H21	-	73.3	H21	H18
22	CH	4.05	m	-	H21/H23	-	70.7	H21/H23	-
23	CH_2	2.33	m	H23	H22	-	46.6	-	-
		2.16	dd (8.7, 15.2)	H23	H22	-			
3	CH	2.42	m	-	H2/H4/H16	-	30.2	H2/H4/H16	H1
7	CH	2.39	m	-	H6/H8/H17	H5	33.4	H6	H5/H18
9	CH	2.35	m	-	H8/H1	H4	39.7	H1	H2/H4/H5/H7/H18
12	CH	2.33	m	-	H13/H15	-	44.5	H13/H15	H14
2	CH_2	1.93	m	-	H3/H1	-	35.0	-	H4/H16
21	CH_2	1.57	m	H21	H20/H22	-	46.3	-	H19/H23
		1.51	m	H21	H20/H22	-		-	
8	CH	1.63	m	-	H9/H7/H18	-	39.7	H9/H7/H18	H6
13	CH_2	1.59	m	H13	H12/H14	-	29.6	H12/H14	H15
		1.45	m	H13	H12/H14	-			
19	CH_2	1.53	m	H19	H18/H20	-	37.2	H18	-
		1.15	m	H19	H18/H20	-			
18	CH_2	1.32	m	-	H8/H19	-	27.3	H19	-
				-	H8/H19	-			
15	CH_3	1.08	d (6.9)	-	H12	-	18.7	H12	H13
16	CH_3	1.05	d (7.4)	-	H3	-	25.1	-	H2
17	CH_3	0.87	d (6.9)	-	H7	-	16.1	H7	-
14	CH_3	0.86	t (7.4)	-	H13	-	14.1	H13	H12
10	Cq	-	-	-	-	-	135.0	H9/H5	H6/H1
24	Cq	-	-	-	-	-	181.5	H23	-
11	Cq	-	-	-	-	-	180.9	H12	H13/H15/H1

Compactin (CP)
(mevastatin)

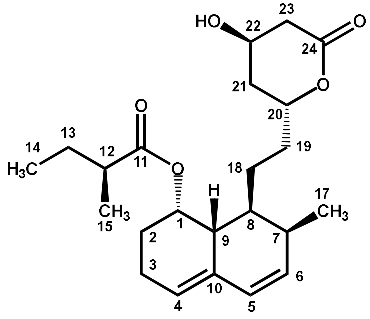

Positions		δ ^{1}H (ppm)	Multiplicity[1] (J (Hz))	COSY ^{1}H-^{1}H correlations			δ ^{13}C (ppm)	HMBC ^{1}H-^{13}C correlations	
				$^2J_{HH}$	$^3J_{HH}$	$^4J_{HH}$		$^2J_{CH}$	$^3J_{CH}$
5	CH	6.00	d (9.7)	-	H6	H7	130.8	-	H4/H7
6	CH	5.79	dd (6.0, 9.7)	-	H5/H7	-	135.9	H7	H17
4	CH	5.57	m	-	H3	H9	126.5	-	H2/H5/H9
1	CH	5.30	m	-	H2/H9	-	70.8	-	H3
20	CH	4.60	m	-	H19/H21	-	79.7	H19	H18/H22
22	CH	4.25	app quint (3.9)	-	H21/H23	-	64.6	H21/H23	H20
23	CH_2	2.69<br>2.51	Ad (4.8, 17.6)<br>Bdd (1.7, 3.6, 17.6)	H23<br>H23	H22<br>H22	-<br>-	40.7	-	H21
3	CH_2	2.15	m	-	H2/H4	-	23.4	H2/H4	H1
7	CH	2.42	m	-	H6/H8/H17	H5	33.5	H6/H17	H5/H18
9	CH	2.42	m	-	H8/H1	H4	39.8	H1	H2/H4/H5/H7/H18
12	CH	2.38	m	-	H13/H15	-	44.5	H13/H15	H14
2	CH_2	2.08<br>1.72	m<br>m	-	H3/H1<br>H3/H1	-	28.6	-	H4
21	CH_2	1.90<br>1.72	m<br>m	H21<br>H21	H20/H22<br>H20/H22	-<br>-	37.9	-	H19/H23
8	CH	1.68	m	-	H9/H7	-	39.6	H7/H9/H18	H6
13	CH_2	1.62<br>1.46	app qd (7.6, 13.6)<br>m	H13<br>H13	H12/H14<br>H12/H14	-<br>-	29.5	H12/H14	H15
19	CH_2	1.81<br>1.37	m<br>m	H19<br>H19	H18/H20<br>H18/H20	-<br>-	35.2	H18	-
18	CH_2	1.48<br>1.39	m<br>m	-<br>-	H19<br>H19	-<br>-	26.3	H19	-
15	CH_3	1.11	d (7.0)	-	H12	-	19.1	H12	H13
17	CH_3	0.90	d (7.0)	-	H7	-	15.9	H7	-
14	CH_3	0.89	t (7.5)	-	H13	-	14.0	H13	H12
10	Cq	-	-	-	-	-	136.7	H5/H9	H1/H3/H6
24	Cq	-	-	-	-	-	174.2	H23	-
11	Cq	-	-	-	-	-	179.2	H12	H1/H13/H15

Dihydromonacolin K
(DiMK)

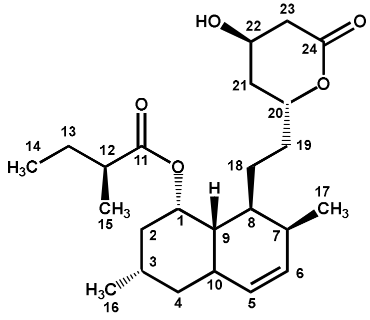

Positions		δ ^{1}H (ppm)	Multiplicity[1] (J (Hz))	COSY ^{1}H-^{1}H correlations $^2J_{HH}$	$^3J_{HH}$	$^4J_{HH}$	δ ^{13}C (ppm)	HMBC ^{1}H-^{13}C correlations $^2J_{CH}$	$^3J_{CH}$
5	CH	5.42	d (9.8)	-	H6	H7	133.8	H10	H4/H7
6	CH	5.69	ddd (2.7, 5.0, 9.8)	-	H5/H7	-	135.6	H7	H17
1	CH	5.15	q (2.7)	-	H2/H9	-	73.1	H2	H3
20	CH	4.58	m	-	H19/H21	-	79.7	H19/H21	-
22	CH	4.25	app quint (3.8)	-	H21/H23	-	64.6	H21/H23	-
23	CH_2	2.68	Ad (4.9, 17.7)	H23	H22	-	40.7	-	-
		2.50	Bdd (1.7, 3.7, 17.7)	H23	H22	H21			
3	CH	2.05	m	-	H4/H16	-	29.5	H2/H4/H16	H1
7	CH	2.34	m	-	H6/H8/H17	H5	33.9	H6	H5/H18
9	CH	1.30	m	-	H8/H1	H4	44.3	H1	H2/H4/H5/H7/H18
10	CH	2.46	m	-	H4/H5/H9	-	33.7	H5	H1/H3/H6
12	CH	2.39	m	-	H13/H15	-	44.6	H13/H15	H14
2	CH_2	1.80	m	-	H3/H1	-	38.0	-	H4/H16
4	CH_2	1.61	m	H4	H10	-	41.1	-	H16
		1.35	m	H4	H10	-			H16
21	CH_2	1.88	m	H21	H20/H22	-	37.8	-	H19/H23
		1.72	m	H21	H20/H22	H23			
8	CH	1.66	m	-	H9/H7	-	40.2	H9/H18	H6
13	CH_2	1.64	m	H13	H12/H14	-	29.3	H12/H14	H15
		1.49	app qd (7.4, 13.6)	H13	H12/H14	-			
19	CH_2	1.81	m	H19	H18/H20	-	35.3	H18	-
		1.37	m	H19	H18/H20	-			
18	CH_2	1.35	m	-	H8/H19	-	25.4	H19	-
		1.30	m	-	H8/H19	-			
15	CH_3	1.12	d (7.0)	-	H12	-	18.8	H12	H13
16	CH_3	1.11	d (7.6)	-	H3	-	23.3	-	H2/H4
17	CH_3	0.86	d (7.0)	-	H7	-	17.0	H7	-
14	CH_3	0.90	t (7.5)	-	H13	-	14.0	H13	H12
24	Cq	-	-	-	-	-	175.2	H23	-
11	Cq	-	-	-	-	-	179.9	H12	H13/H15/H1

[1] d: doublet, dd: doublet of doublet, ddd: doublet of doublet of doublet, t: triplet, q: quadruplet, qd: doublet of quadruplet, quint: quintuplet, hept: heptuplet, m: multiplet, A: part A of an AB system, B: part B of an AB system, app: apparent.

Table S2. ^{1}H NMR quantitative determination of monacolins in RYR dietary supplements.

DS number	Amount of monacolins (mg per capsule or tablet)[1] ± CV (RSD) (n = 3)				
	**Signal at 5.84 ppm[2] H6**	**Signal at 5.56 ppm[2] H4**	**Signal at 5.33 ppm[2] H1**	**Signal at 4.60 ppm[2] H20**	**Signal at 4.25 ppm[2] H22**
1	1.67 ± 0.11 (6.0%)	1.70 ± 0.13 (7.6%)	3.31 ± 0.22 (6.6%)	1.40 ± 0.11 (7.9%)	1.34 ± 0.07 (5.2%)
2	9.14 ± 0.79 (8.7%)	9.18 ± 0.73 (7.9%)	9.26 ± 0.22 (2.4%)		7.10 ± 0.48 (6.8%)
3	3.07 ± 0.36 (11.6%)	3.07 ± 0.27 (8.8%)	2.39 ± 0.07 (2.9%)		
4	1.02 ± 0.06 (5.9%)	0.95 ± 0.07 (7.4%)	1.64 ± 0.03 (1.8%)		0.71 ± 0.02 (2.8%)
5	2.47 ± 0.14 (5.8%)	2.53 ± 0.15 (5.8%)	11.3 ± 1.1 (9.4%)	2.15 ± 0.08 (3.8%)	
6	2.92 ± 0.08 (2.7%)	2.63 ± 0.20 (7.6%)	3.27 ± 0.07 (2.1%)		2.19 ± 0.15 (6.9%)
7	3.76 ± 0.29 (7.6%)	3.73 ± 0.37 (10.0%)			
8	8.68 ± 0.43 (4.9%)	8.79 ± 0.29 (3.3%)	8.65 ± 0.15 (1.7%)	6.89 ± 0.46 (6.7%)	7.73 ± 0.37 (4.8%)
9	5.14 ± 0.44 (8.5%)	5.21 ± 0.36 (6.9%)	8.03 ± 0.20 (2.5%)	4.55 ± 0.35 (7.6%)	4.55 ± 0.40 (8.8%)
10	2.10 ± 0.06 (3.0%)	2.04 ± 0.27 (13.2%)	8.67 ± 0.13 (1.5%)		1.22 ± 0.07 (5.8%)
11					
12					
13	23.9 ± 1.8 (7.6%)	23.9 ± 1.6 (6.9%)	21.9 ± 0.5 (2.4%)	23.9 ± 0.4 (1.7%)	23.2 ± 1.9 (8.3%)
14	3.45 ± 0.10 (2.8%)	3.28 ± 0.26 (7.9%)	10.8 ± 0.2 (1.5%)		2.26 ± 0.10 (4.2%)
15	11.9 ± 0.2 (1.6%)	12.3 ± 0.3 (2.6%)		9.85 ± 0.53 (5.4%)	
16		0.31 ± 0.03 (8.7%)			
17	1.47 ± 0.04 (2.7%)	1.51 ± 0.08 (5.0%)	4.06 ± 0.07 (1.7%)	1.28 ± 0.14 (11.0%)	
18	8.84 ± 0.41 (5.0%)	8.19 ± 0.30 (3.7%)		6.68 ± 0.39 (5.8%)	7.04 ± 0.69 (9.8%)

19	0.36 ± 0.02 (5.6%)	0.44 ± 0.04 (6.9%)			
20	1.75 ± 0.07 (4.0%)	1.77 ± 0.08 (4.2%)	3.51 ± 0.21 (6.0%)	1.27 ± 0.08 (6.3%)	1.49 ± 0.08 (5.4%)
21	0.48 ± 0.06 (12.5%)	0.47 ± 0.06 (12.8%)			
22	3.09 ± 0.17 (5.4%)	3.02 ± 0.26 (8.5%)			2.26 ± 0.17 (7.6%)
23	10.6 ± 0.2 (1.9%)	10.7 ± 1.0 (9.2%)	10.0 ± 0.9 (8.9%)	10.0 ± 1.2 (11.8%)	9.77 ± 0.71 (7.3%)
24	11.5 ± 0.7 (5.9%)	11.8 ± 0.8 (6.7%)	9.38 ± 0.61 (6.5%)		5.97 ± 0.12 (2.0%)
25	10.0 ± 0.3 (2.6%)	9.72 ± 0.20 (2.1%)		5.97 ± 0.12 (2.0%)	7.26 ± 0.63 (8.7%)
26	4.51 ± 0.35 (7.4%)	4.54 ± 0.19 (4.2%)			3.88 ± 0.25 (6.5%)
27	4.13 ± 0.21 (5.1%)	3.84 ± 0.24 (6.3%)		2.97 ± 0.32 (10.8%)	2.90 ± 0.26 (9.0%)
28	1.64 ± 0.10 (6.1%)	1.75 ± 0.15 (8.6%)		1.05 ± 0.09 (8.6%)	1.31 ± 0.14 (10.7%)
29					
30	2.99 ± 0.11 (3.7%)	3.04 ± 0.12 (3.9%)	2.61 ± 0.05 (1.9%)		2.43 ± 0.23 (9.5%)
31	1.21 ± 0.10 (8.6%)	1.28 ± 0.11 (8.6%)	2.16 ± 0.09 (4.2%)		

[1] The amounts of monacolins (mg per dosage unit) were calculated from the measured areas using the equation presented in the paragraph 3.4.2.

[2] The resonances at 5.84, 5.56 ppm and 5.33 ppm are characteristic of all the monacolins bearing an hexahydronaphthalene ring but the 5.33 ppm signal does not characterize MJ and ML in lactone or in hydroxyl acid form; the resonances at 4.60 and 4.25 ppm are specific of all the monacolins in lactone form including dihydromonacolins.

Table S3. Comparison of monacolin amounts measured in RYR dietary supplements by ^{1}H NMR and UHPLC.

NMR signal considered	Number of samples	Quantified monacolins (M)[1]	Linear regression equation y = a + bx [2]			p-value[3]
			b ± Sb	a ± Sa	r^2	
5.84 ppm	27	TotalM - DiMK	0.986 ± 0.018	0.004 ± 0.130	0.992	0.9365
5.56 ppm	28	TotalM - DiMK	0.984 ± 0.017	0.044 ± 0.117	0.993	0.6570
Mean intensity of the two signals at 5.84 and 5.56 ppm	28	TotalM - DiMK	0.985 ± 0.017	0.025 ± 0.117	0.993	0.6406
4.60 ppm or 4.25 ppm or mean intensity of the two signals	22	All M in lactone form (MJ, MN, MX, ML, CP, MK, DiMK)	0.983 ± 0.015	0.013 ± 0.099	0.996	0.4953
5.33 ppm	17	MN, MX, MKA, CP, MK, DeML, DeMK	0.952 ± 0.152	-1.504 ± 1.321	0.723	0.0191
Mean intensity of the two signals at 5.84 and 5.56 ppm	28	All M determined by ^{1}H NMR (TotalM - DiMK) vs TotalM determined by UHPLC	1.001 ± 0.023	0.113 ± 0.166	0.986	0.2549

[1] The monacolin amount determined by ^{1}H NMR for each RYR dietary supplement (Table 3) was compared to that measured by UHPLC for the same group of monacolins (Table 5) except in the last line of the table.
[2] The statistics for the straight lines were obtained with the LINEST function included in the Microsoft Excel software. b represents the slope of the linear regression and a the y-intercept. Sa and Sb correspond to the standard error values for the constants a and b, and r^2 is based on the Pearson's linear correlation coefficient.
[3] p-values were determined with the Wilcoxon signed-rank test.

Figure S1. ^{1}H NMR spectra (CD_3CN:D_2O (80:20)) of all the dietary supplements analyzed in the present study. Upper part: entire spectrum, lower part: enlarged downfield region (4-9 ppm). FA: fatty acids (saturated and unsaturated); UFA: non-conjugated unsaturated fatty acids; Monacolins OHAc: monacolins in hydroxyl acid form.

DS 1

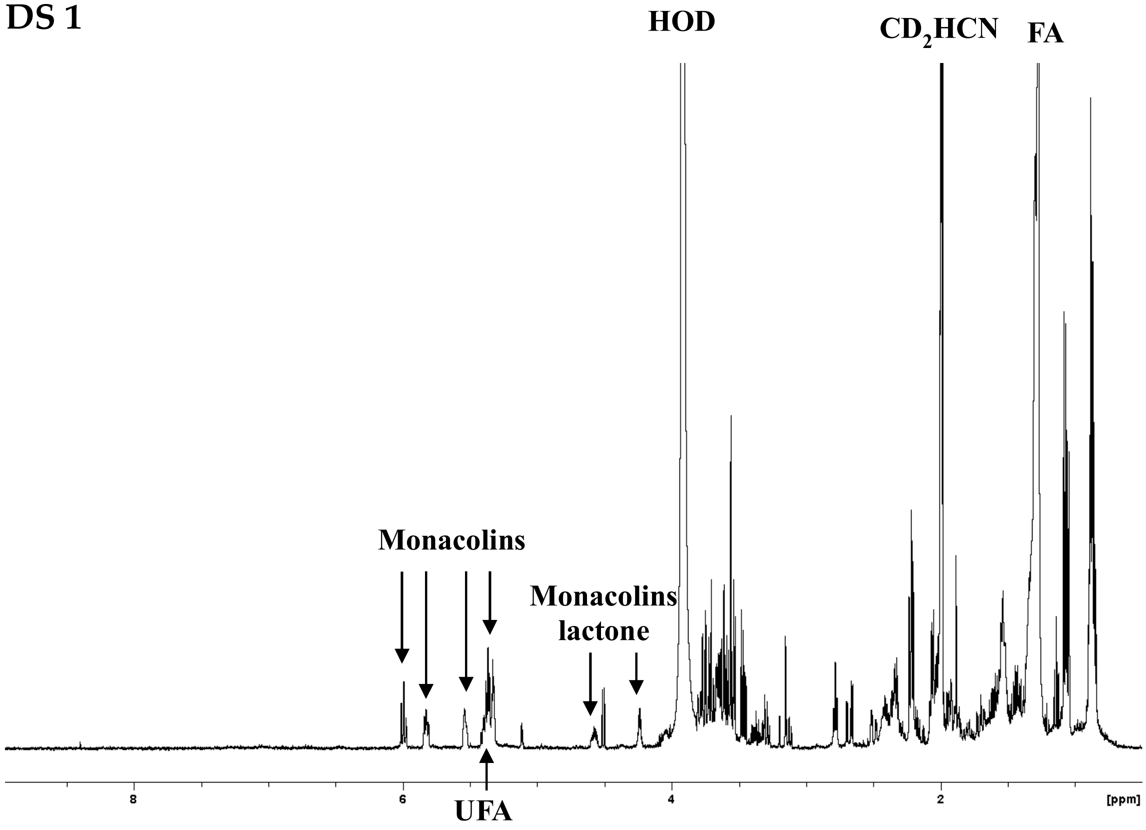

HOD
CD_2HCN
FA
Monacolins
Monacolins lactone
UFA
8
6
4
2
[ppm]

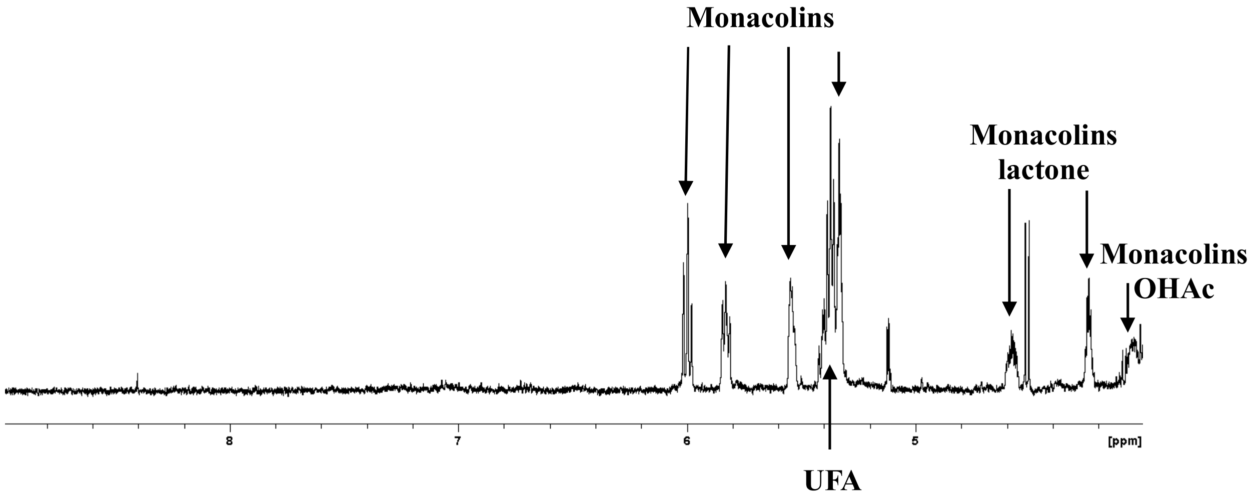

Monacolins
Monacolins lactone
Monacolins OHAc
UFA
8
7
6
5
[ppm]

DS 2

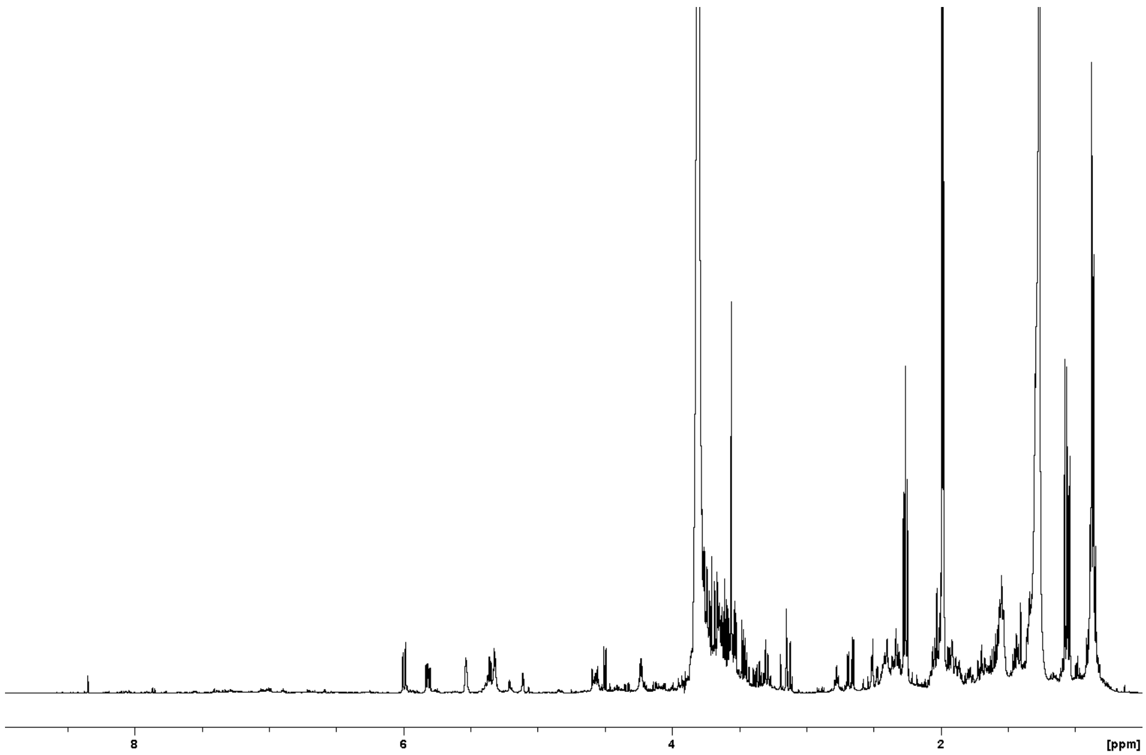

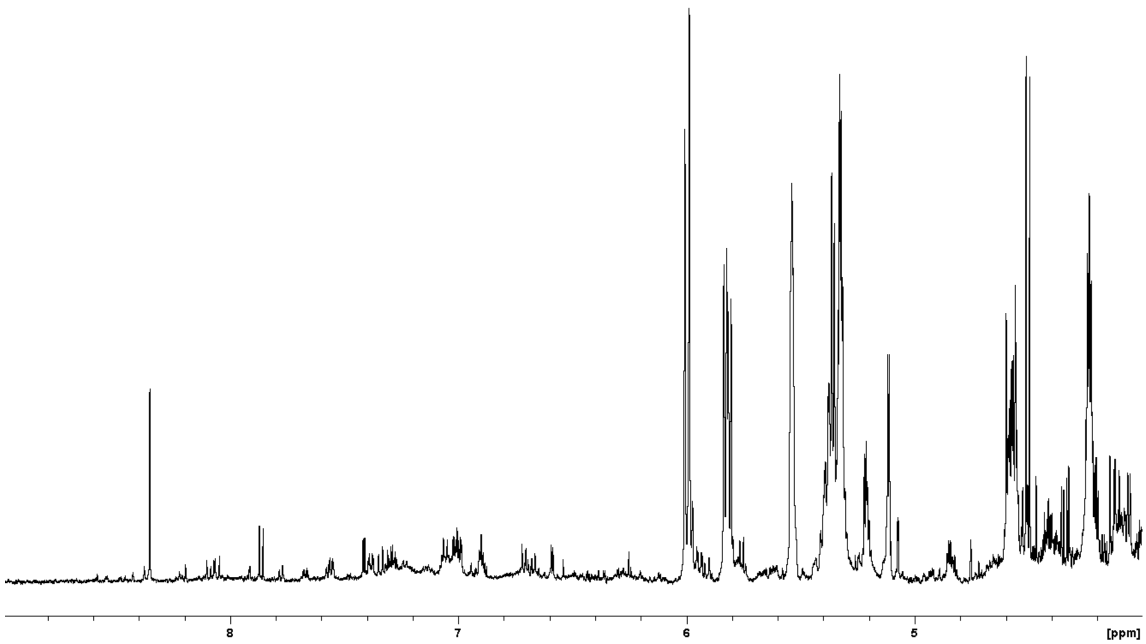

DS 3

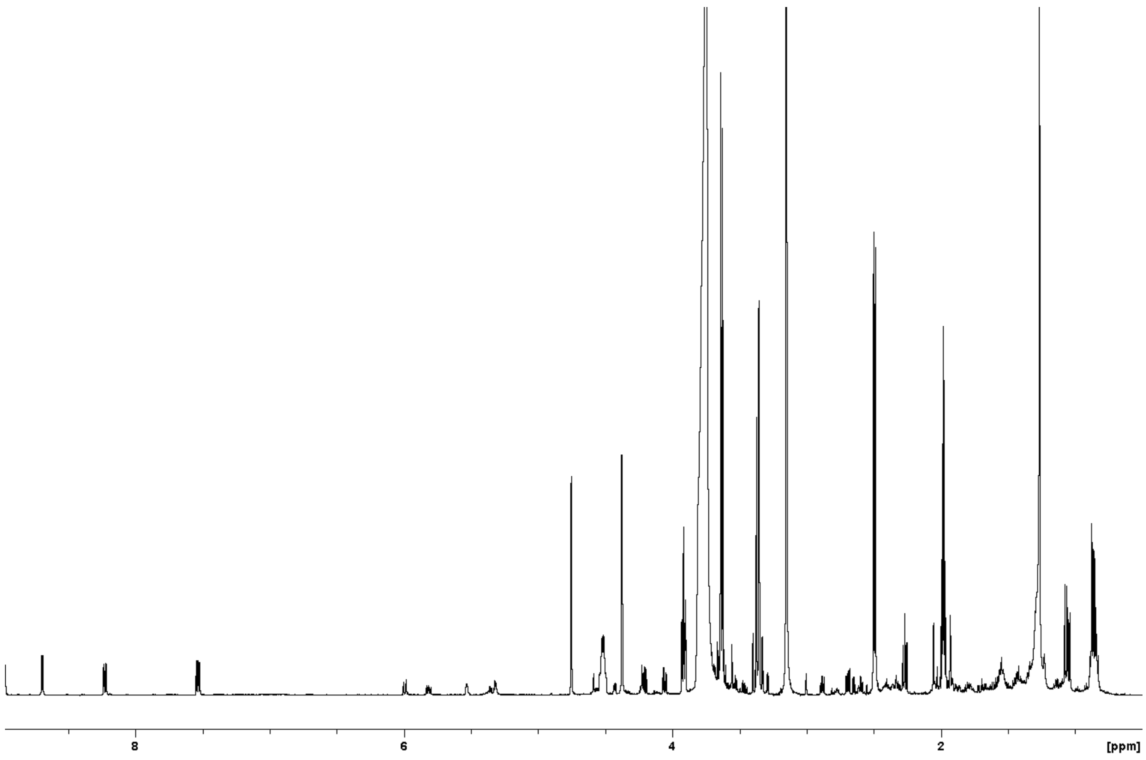

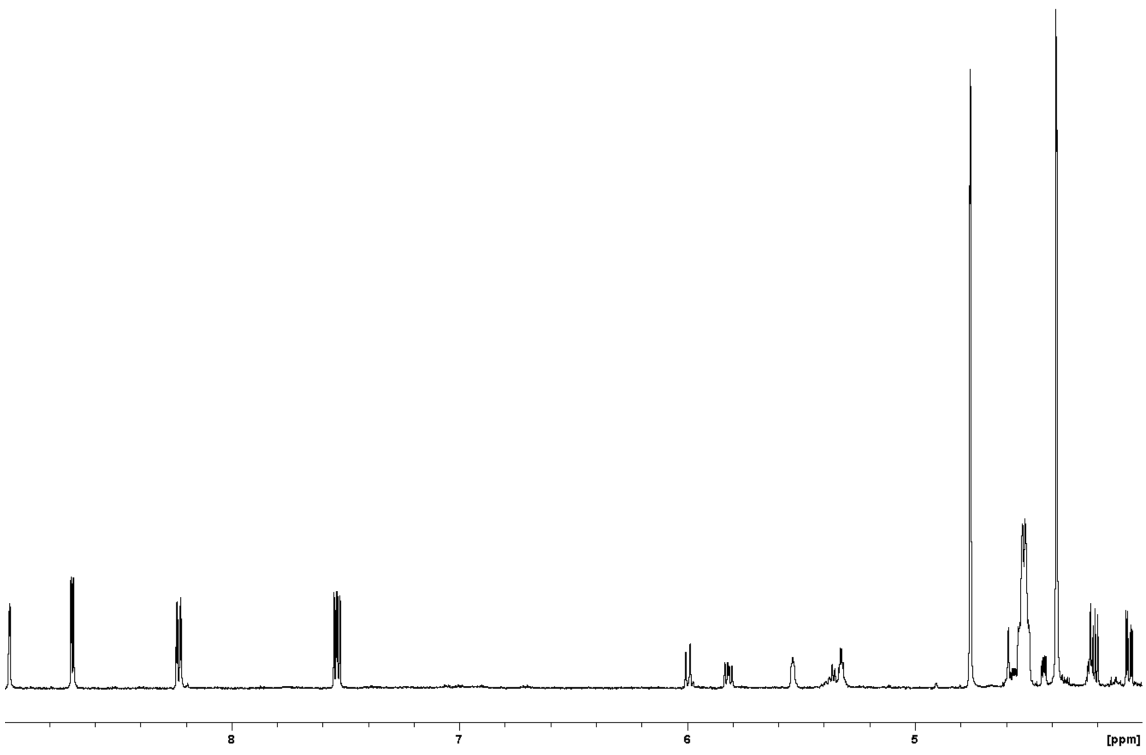

DS 4

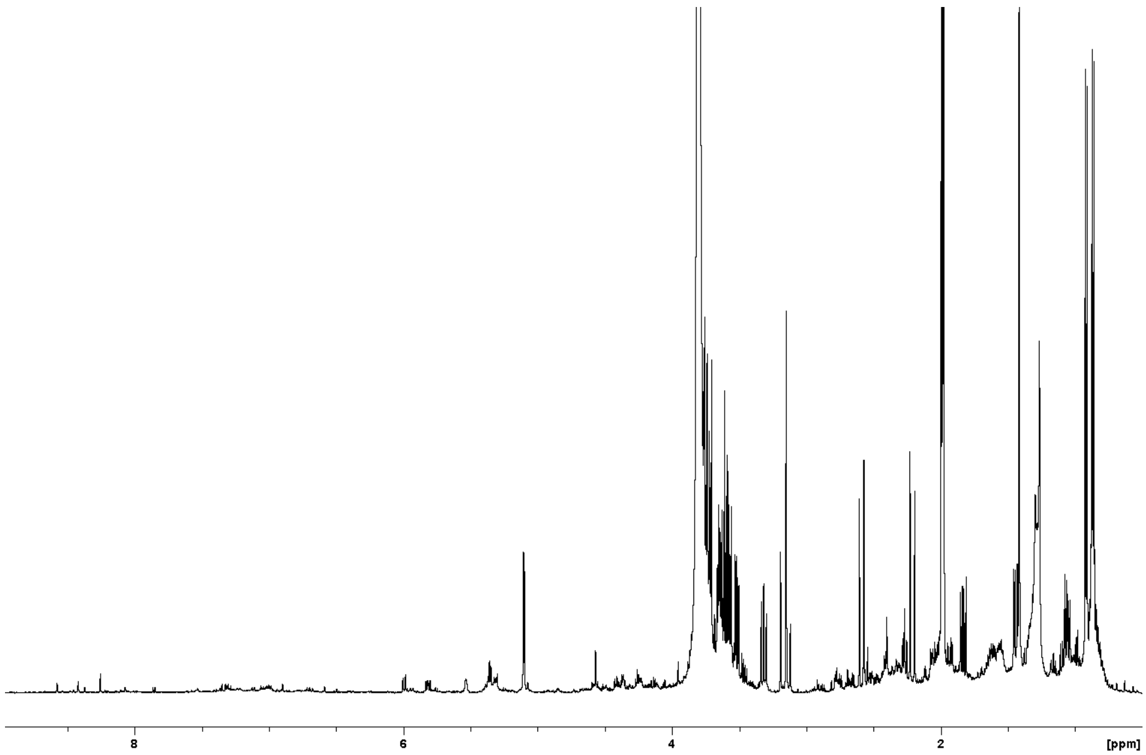

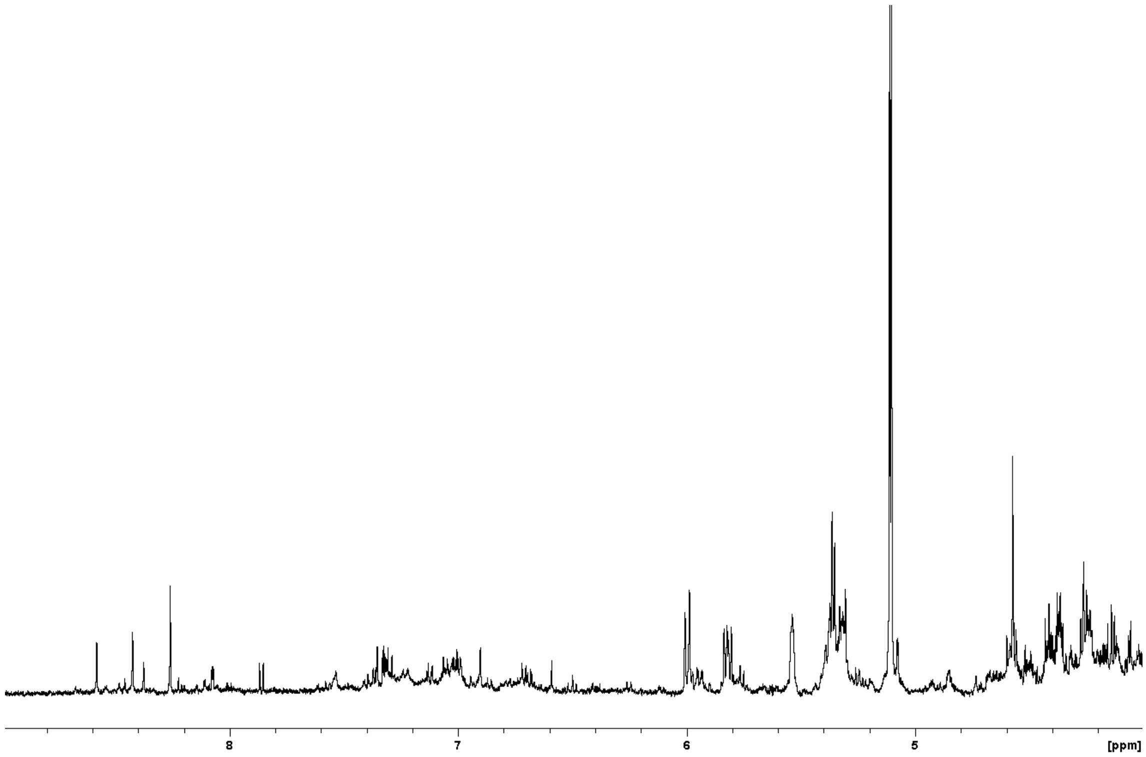

DS 5

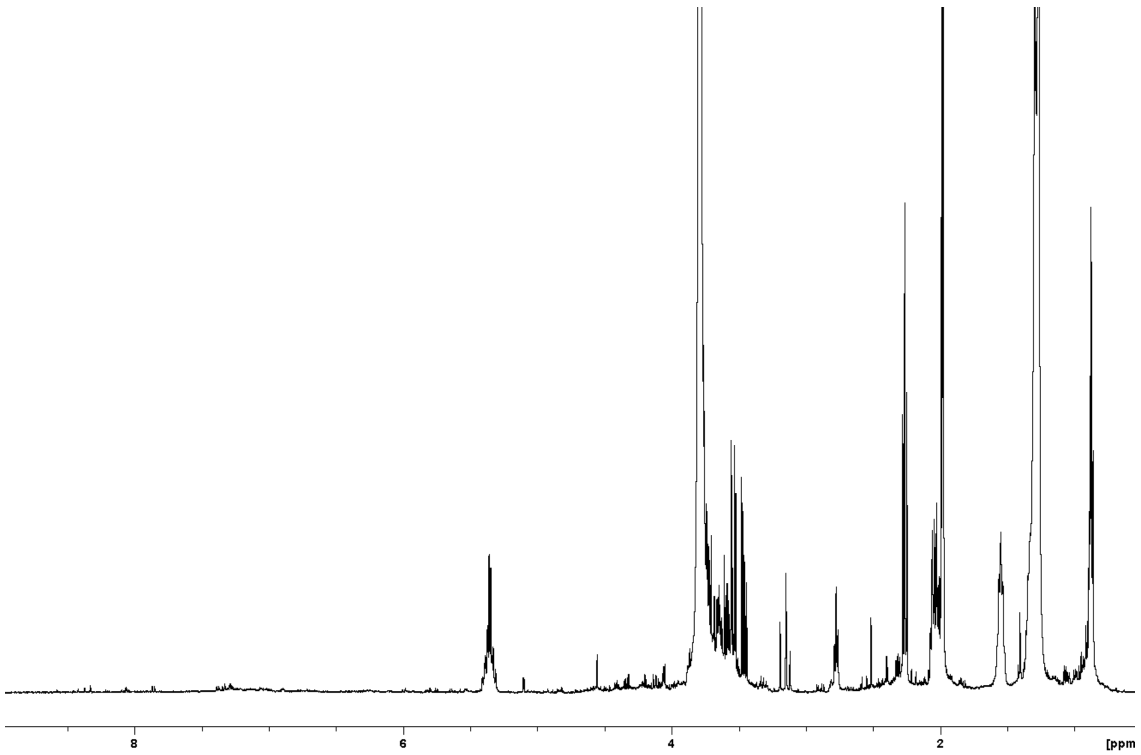

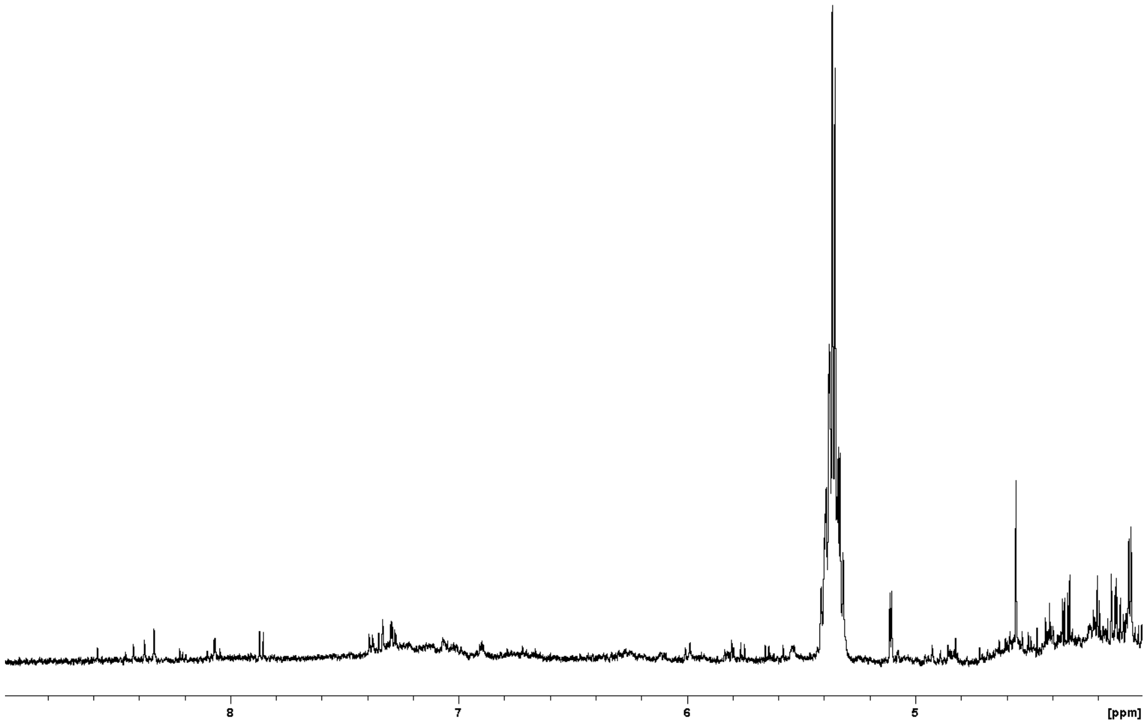

DS 6

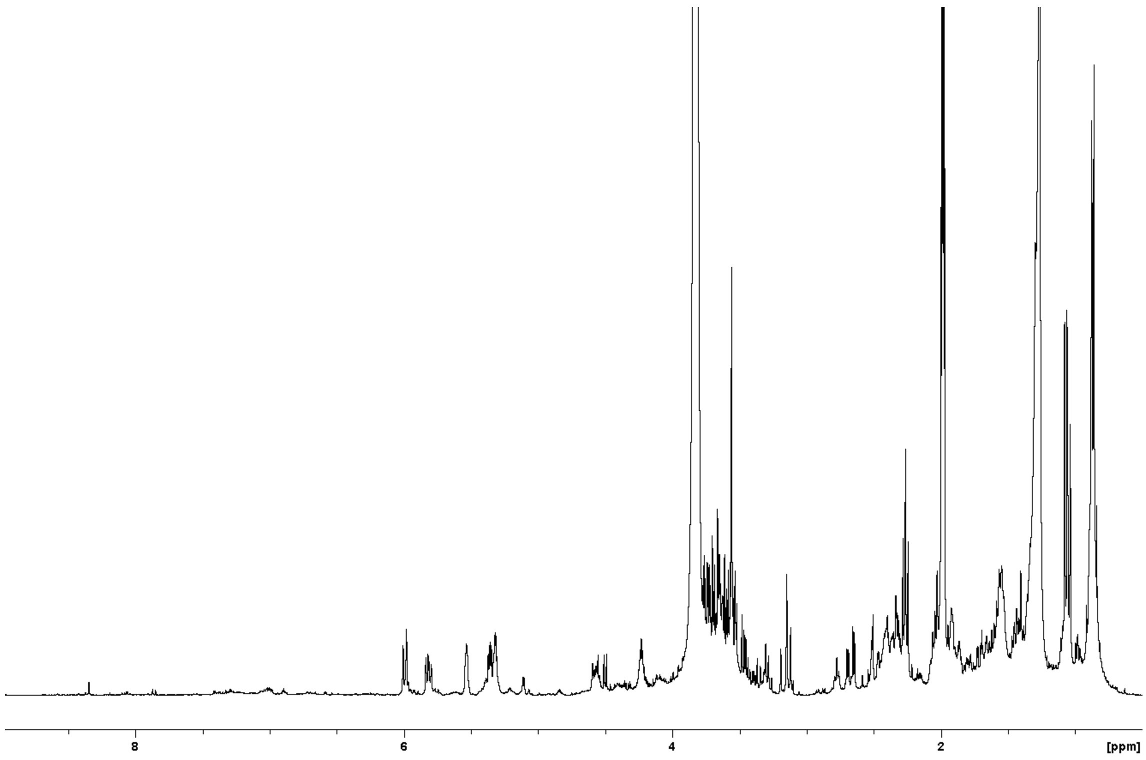

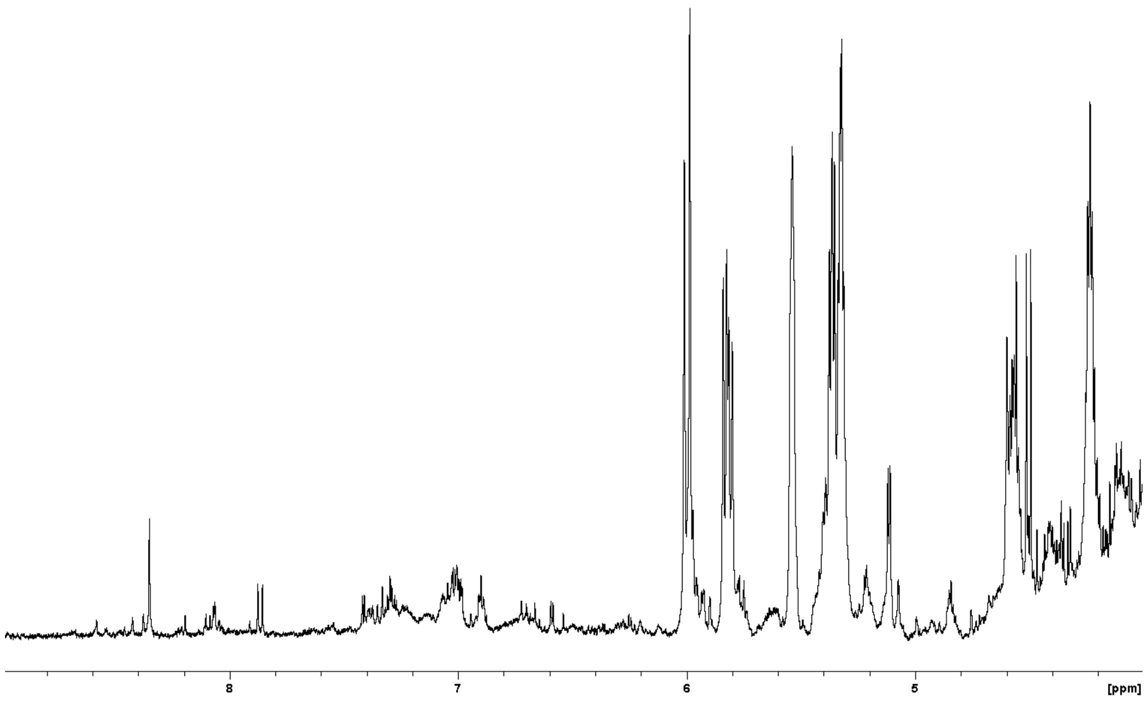

DS 7

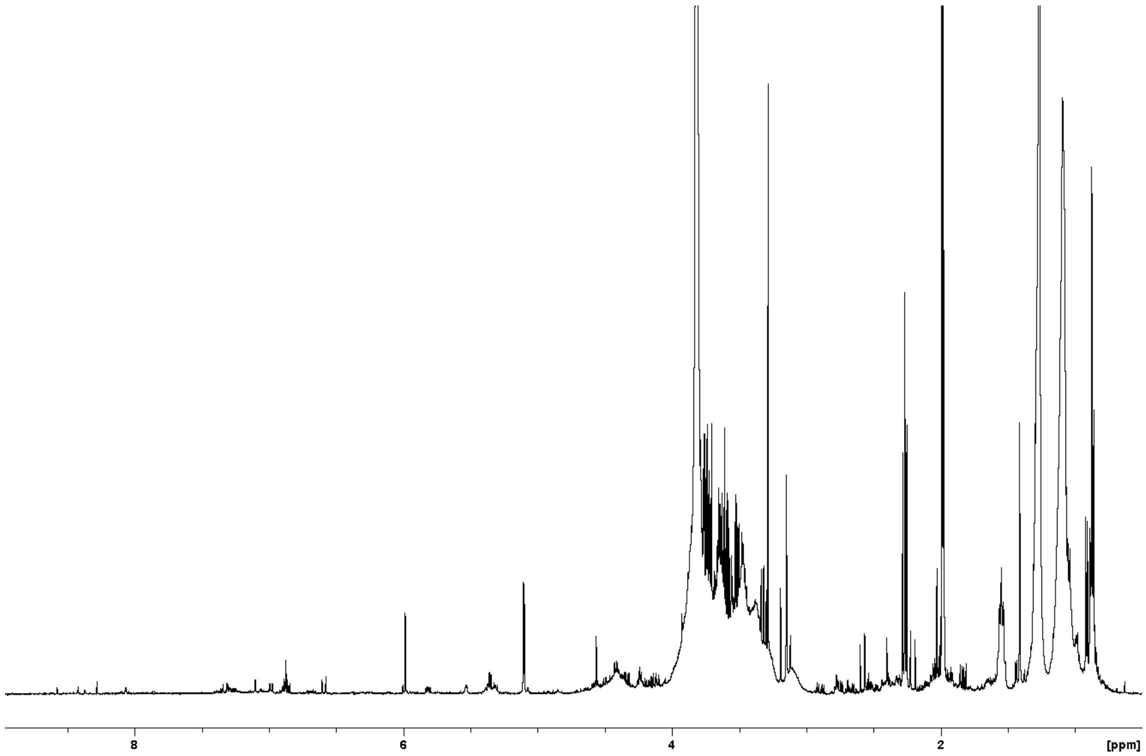

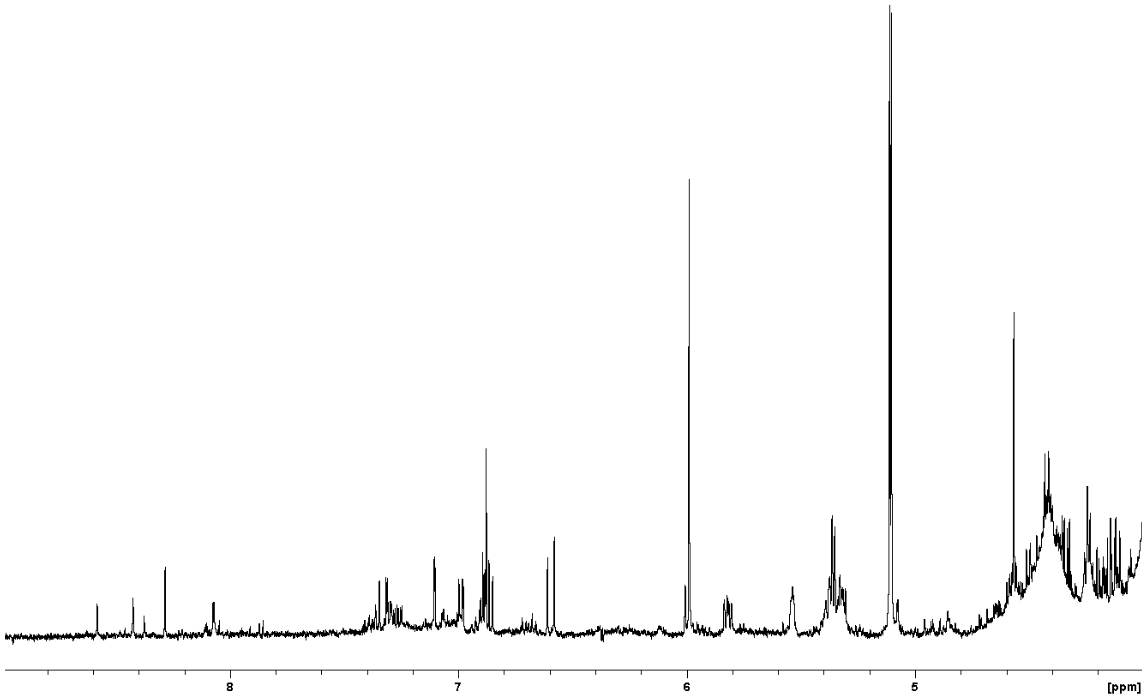

DS 8

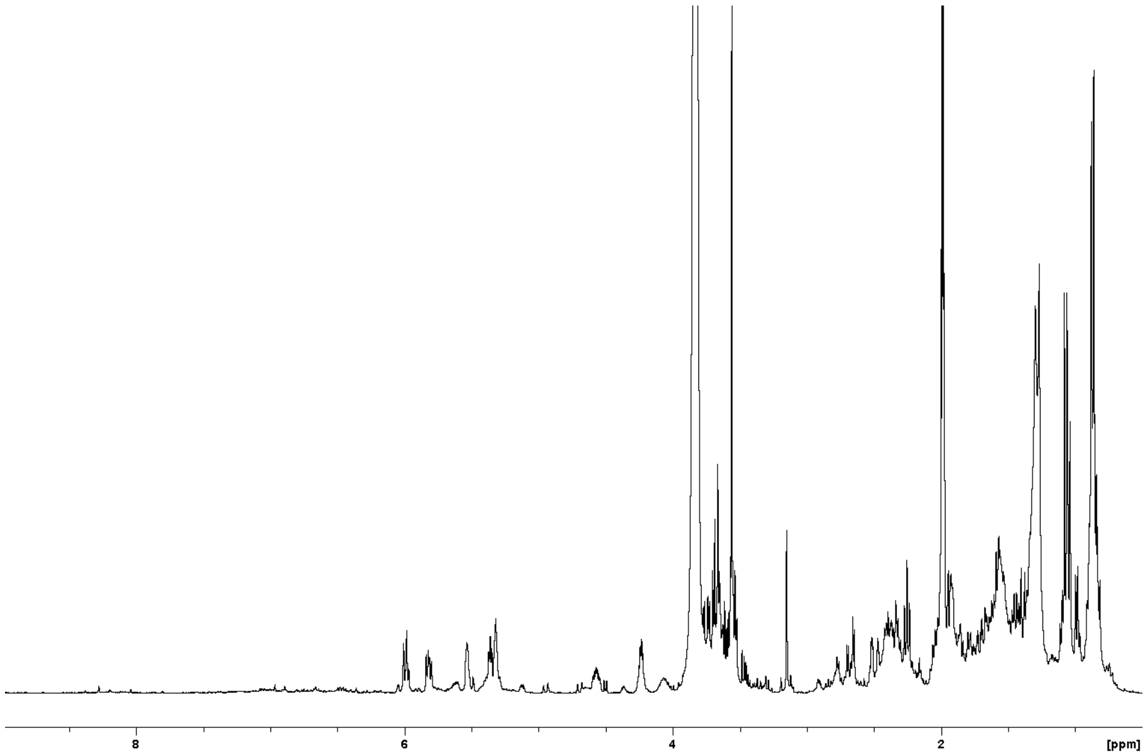

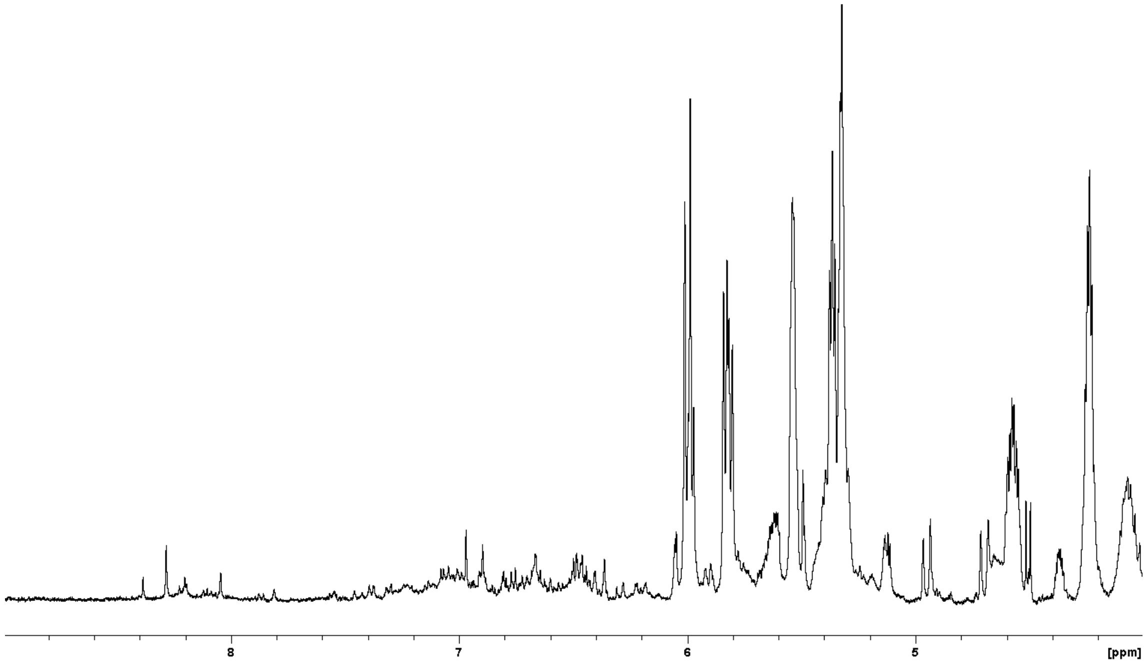

DS 9

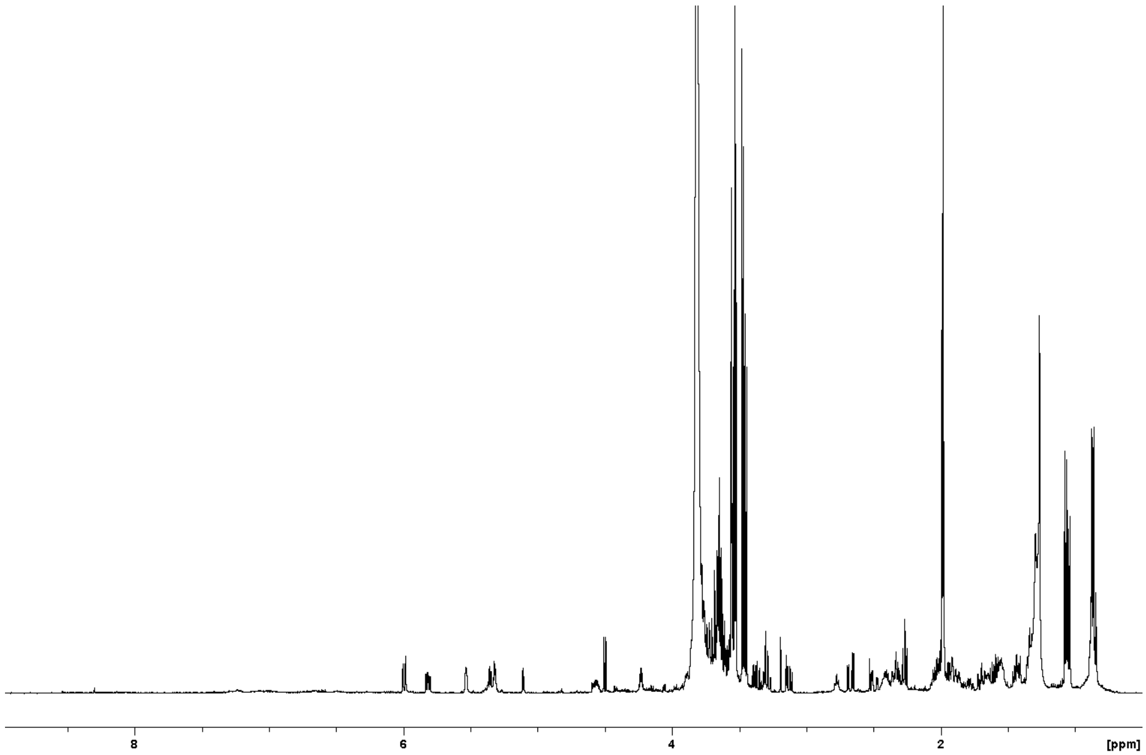

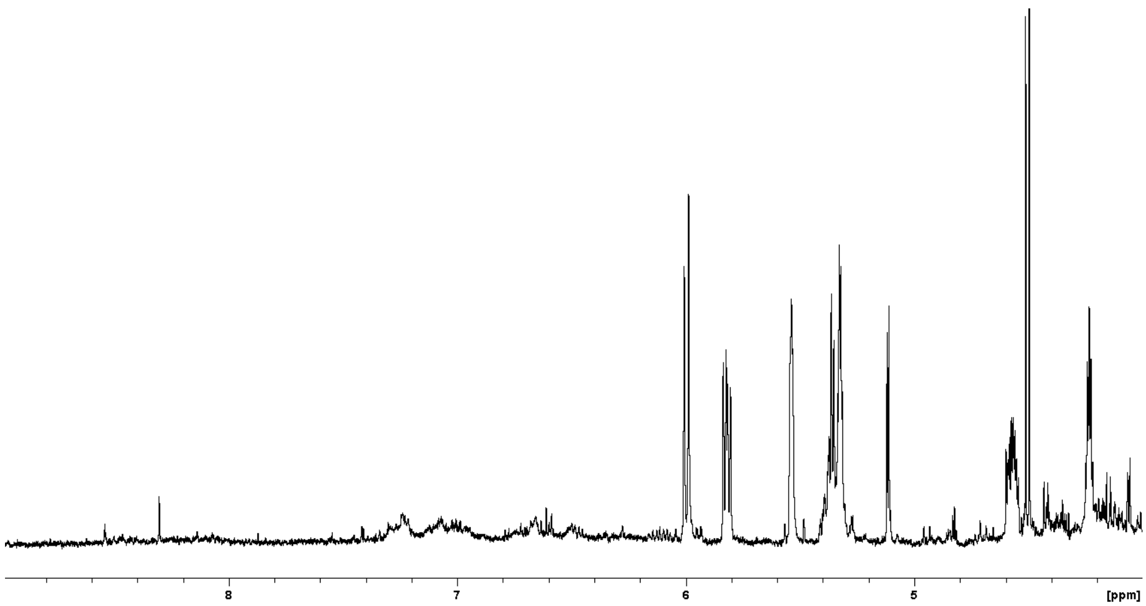

DS 10

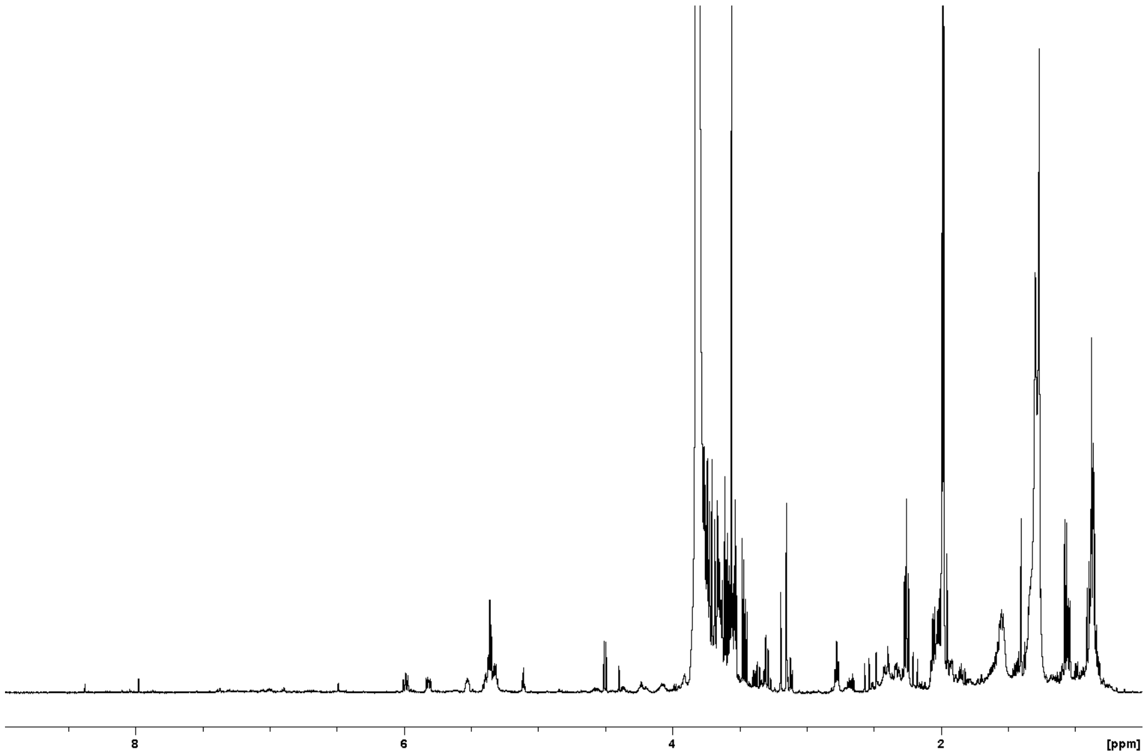

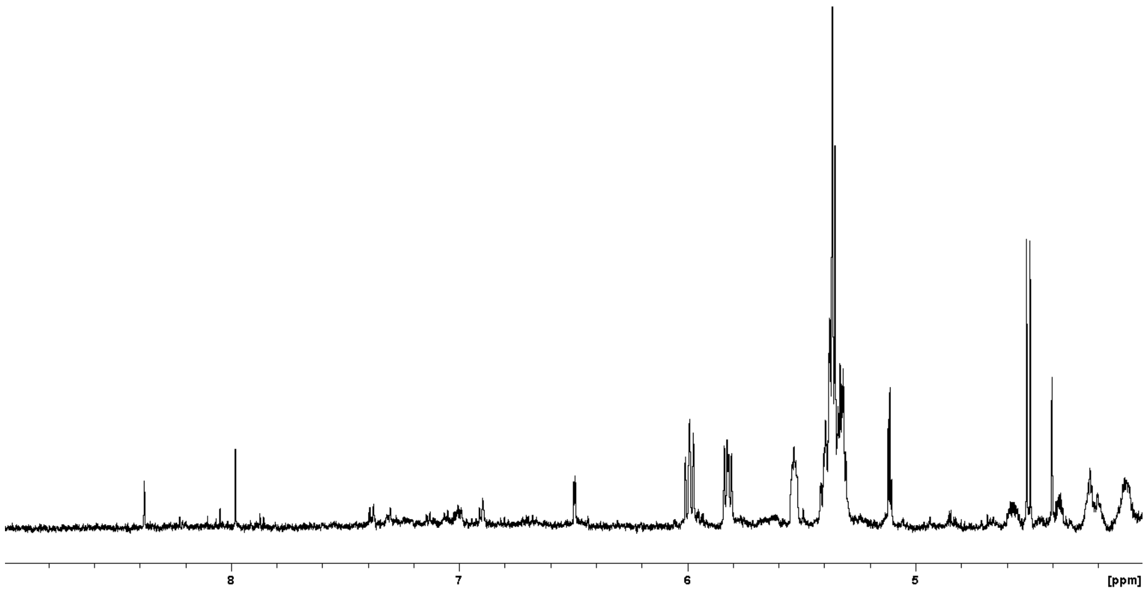

DS 11 No monacolins detected

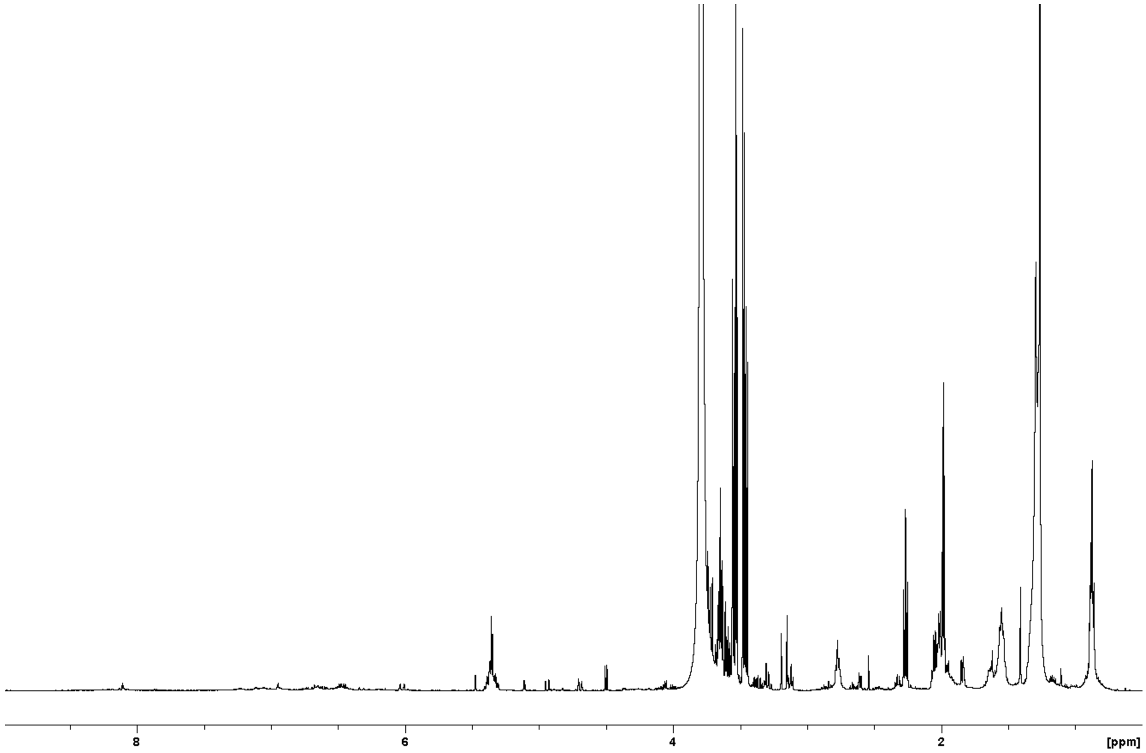

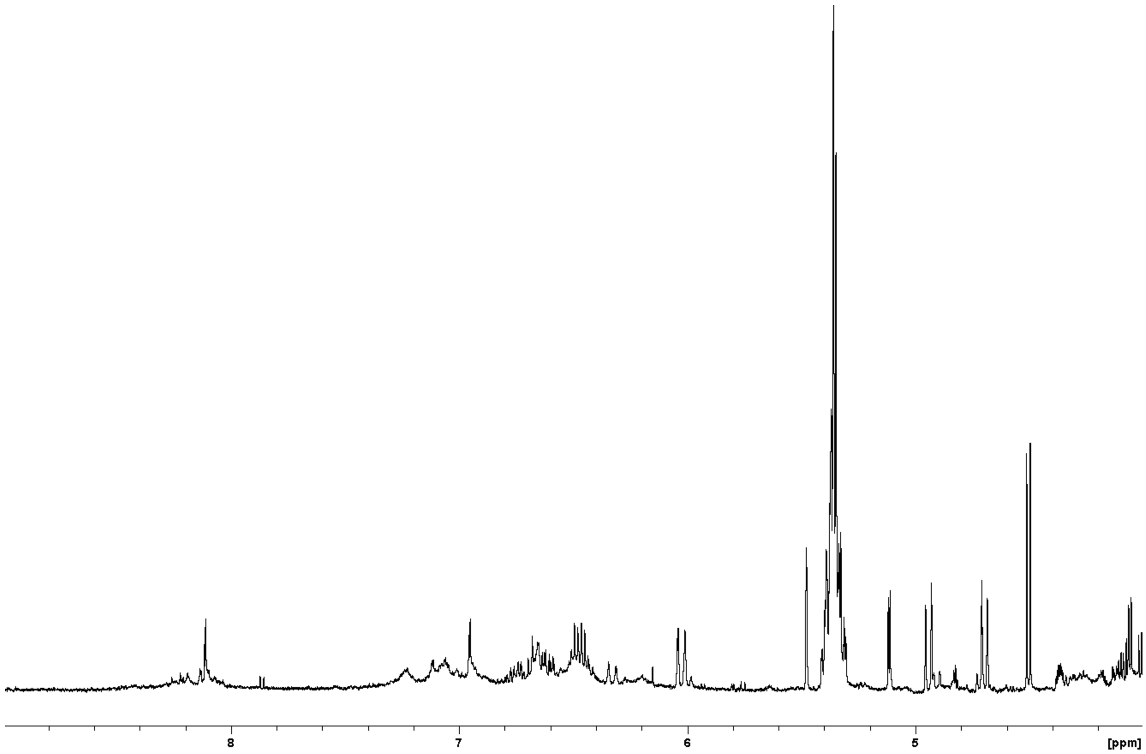

DS 12 No monacolins detected

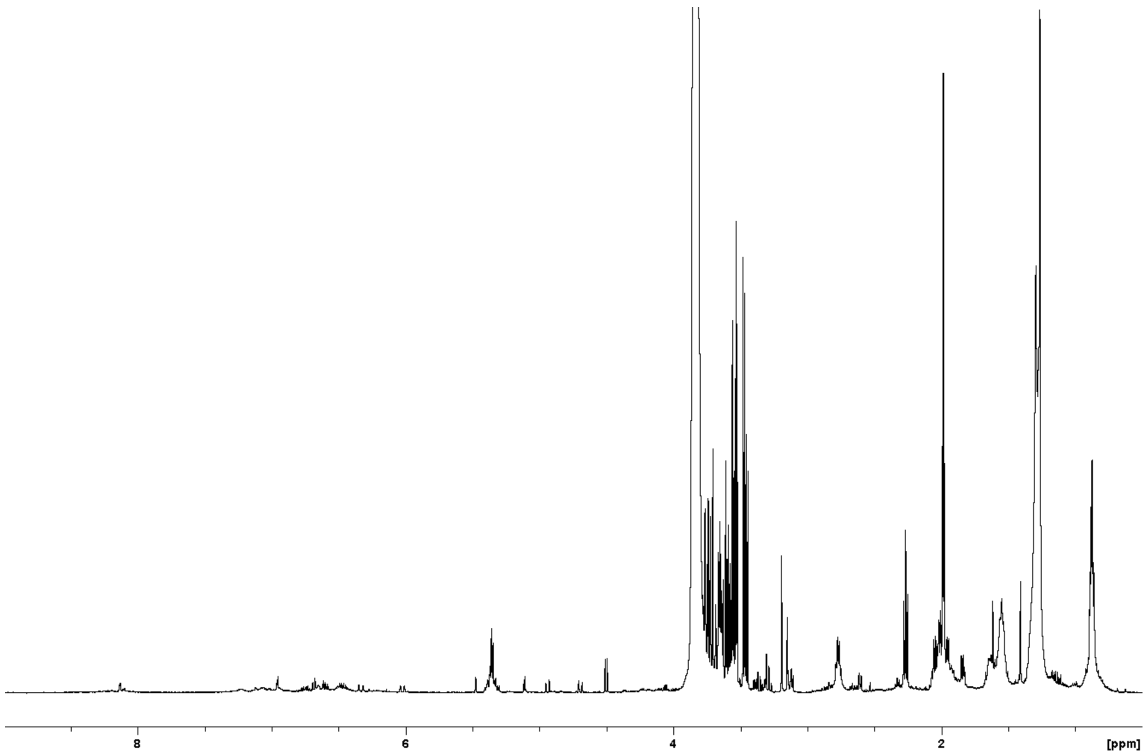

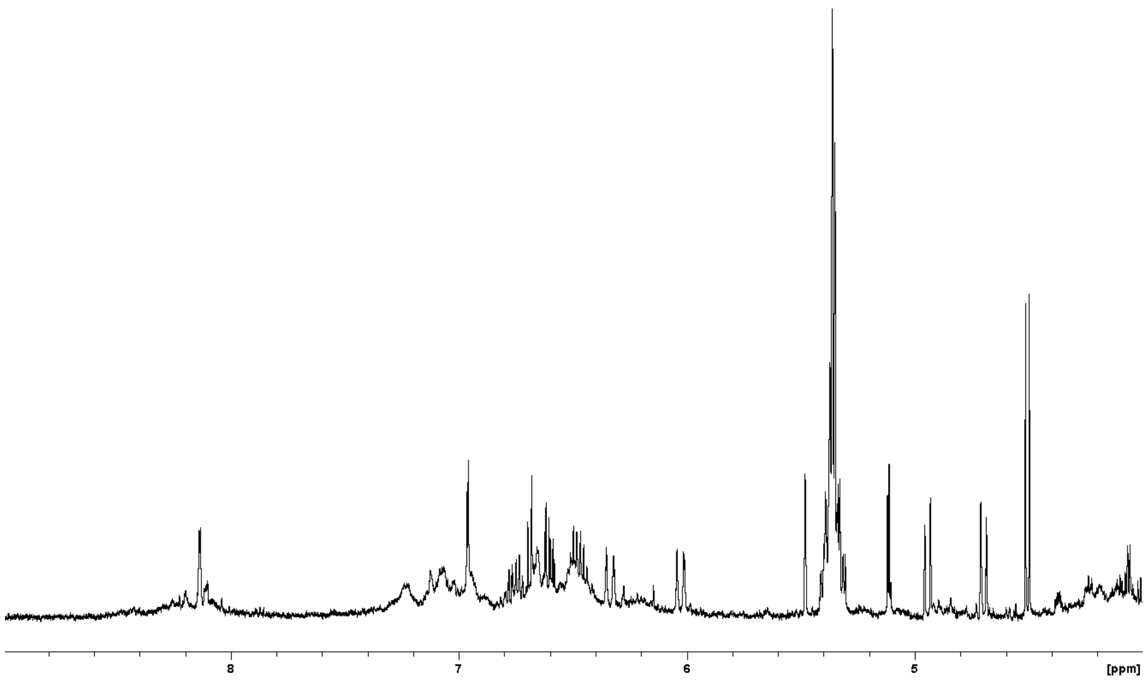

DS 13

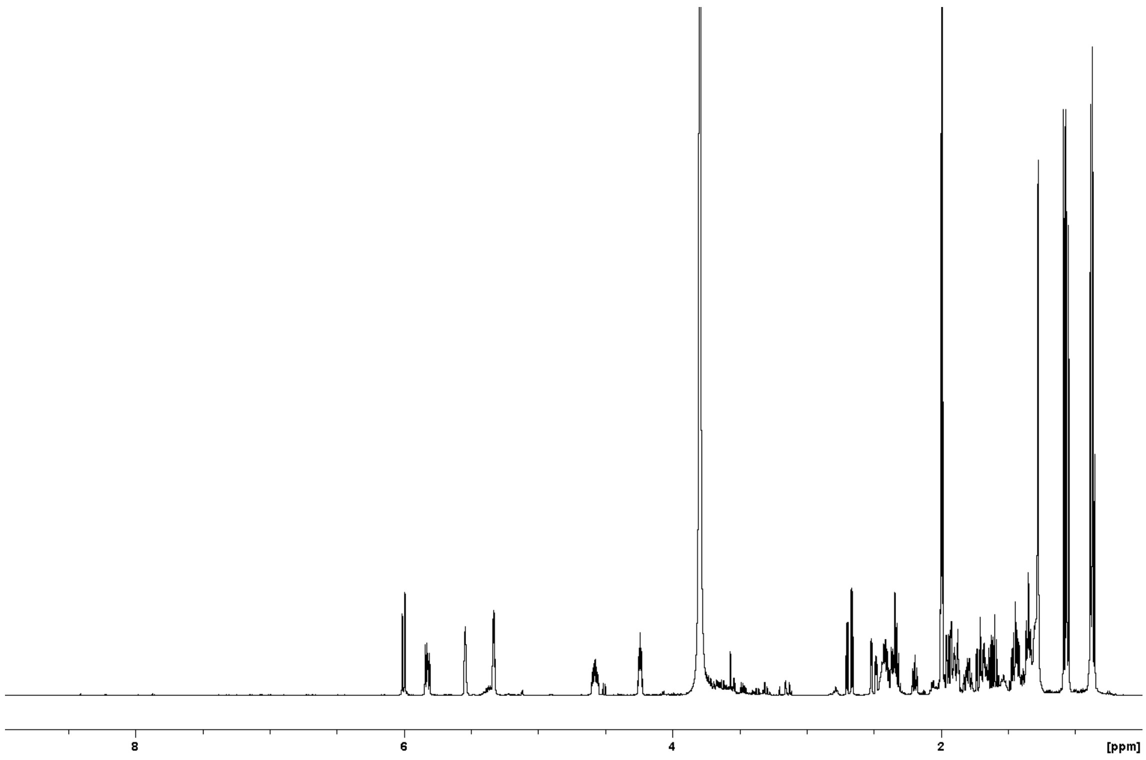

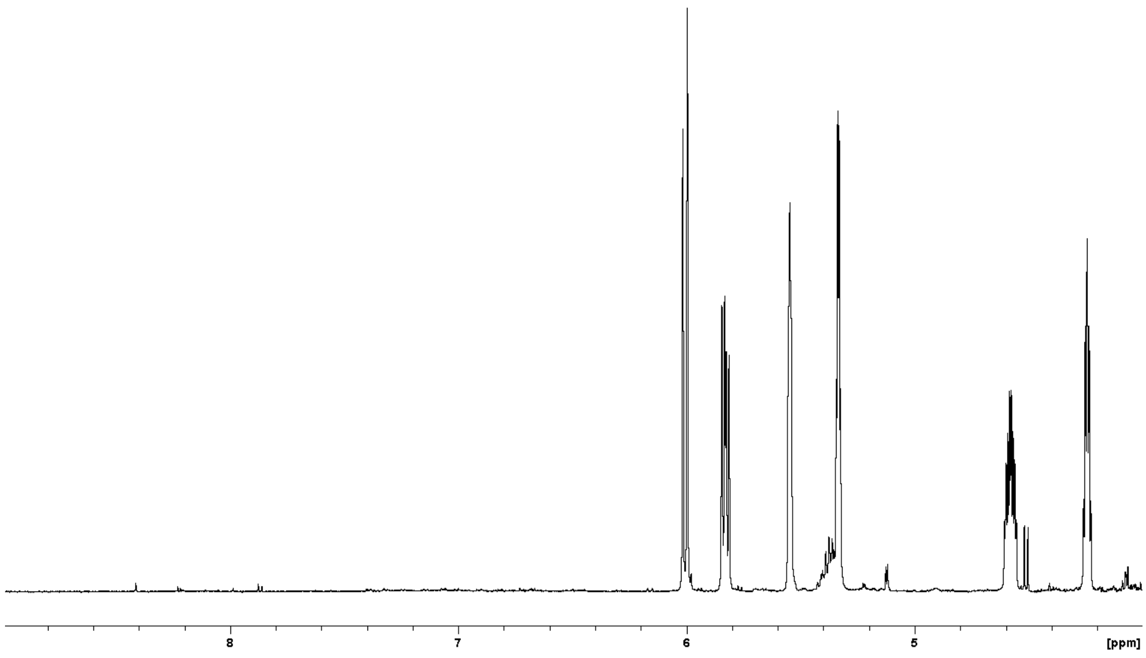

DS 14

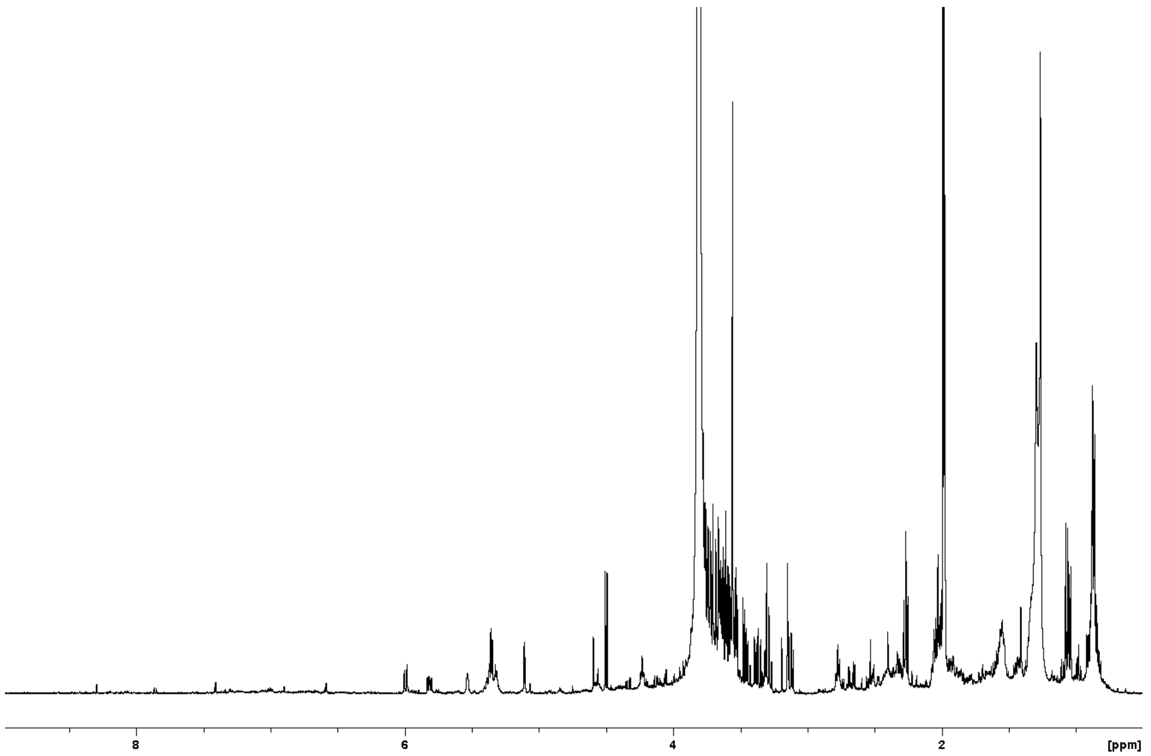

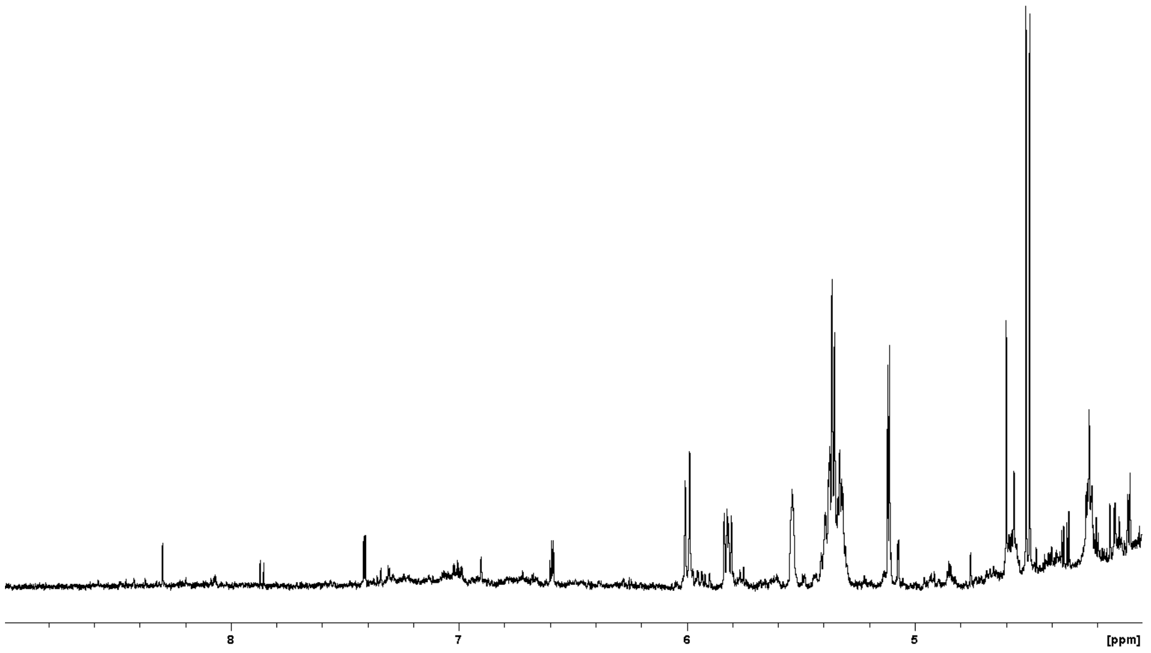

DS 15

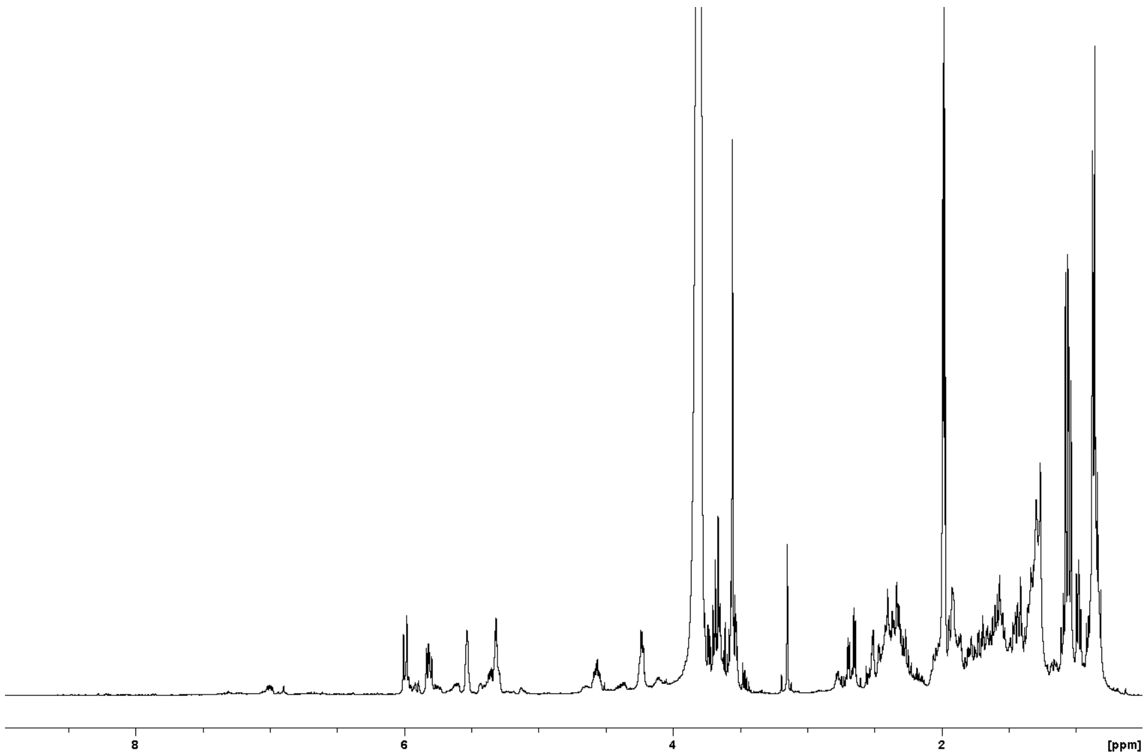

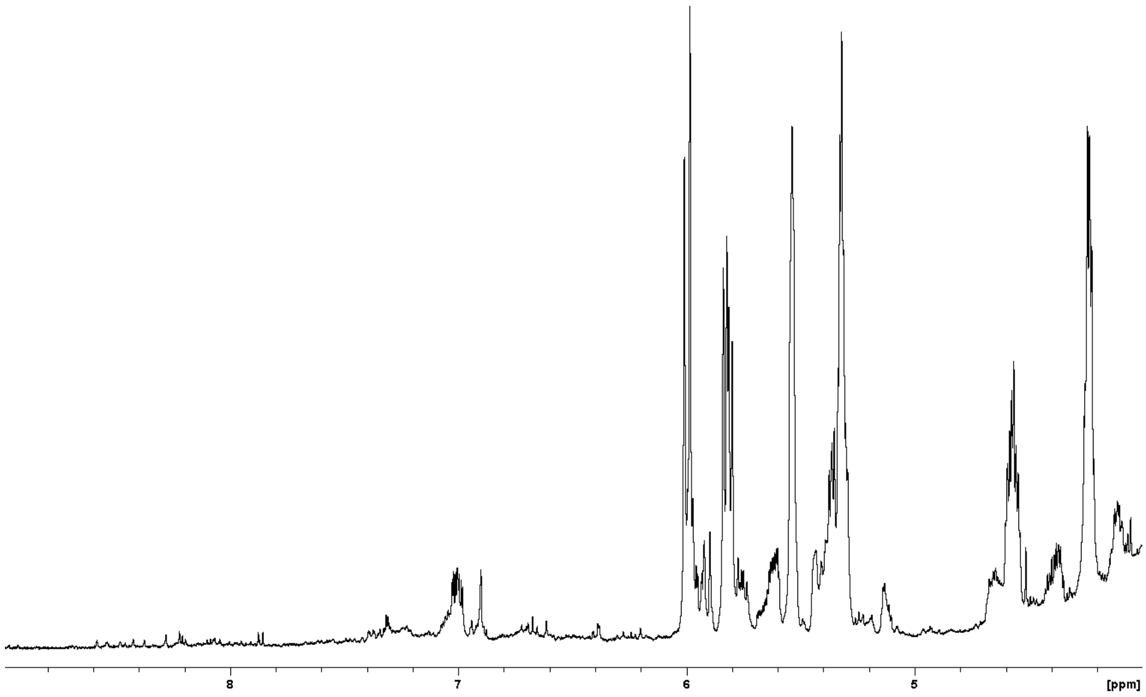

DS 16 Monacolins hardly detected

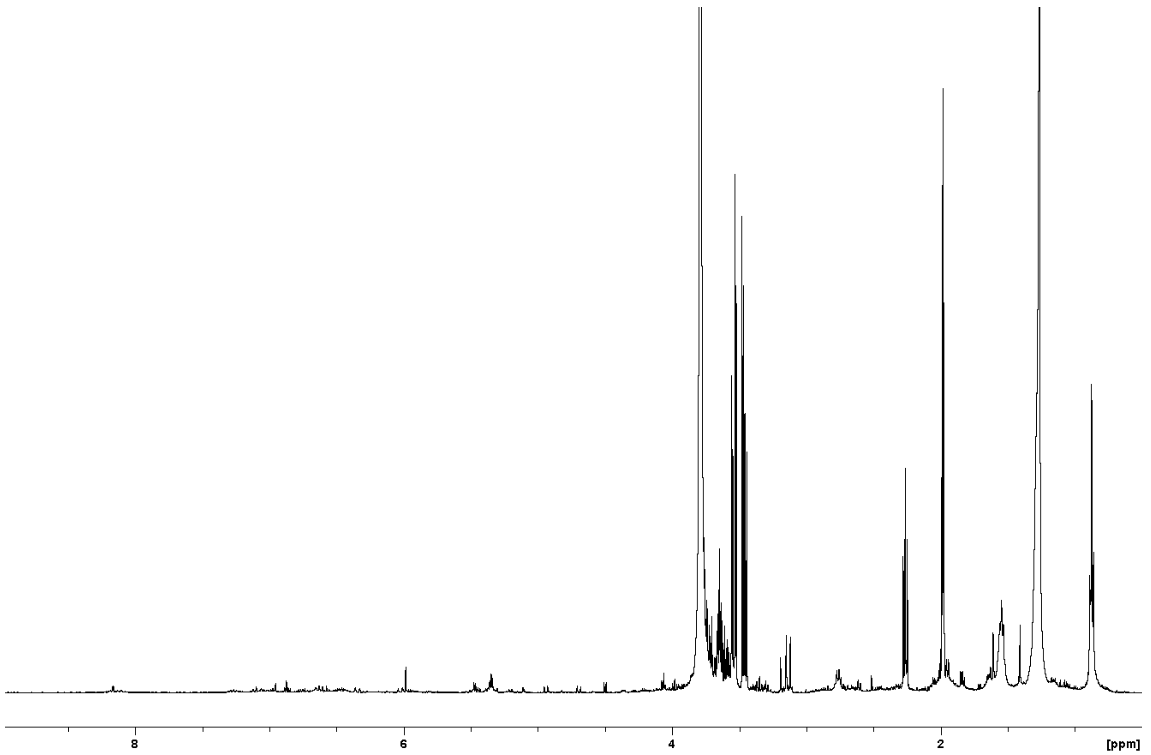

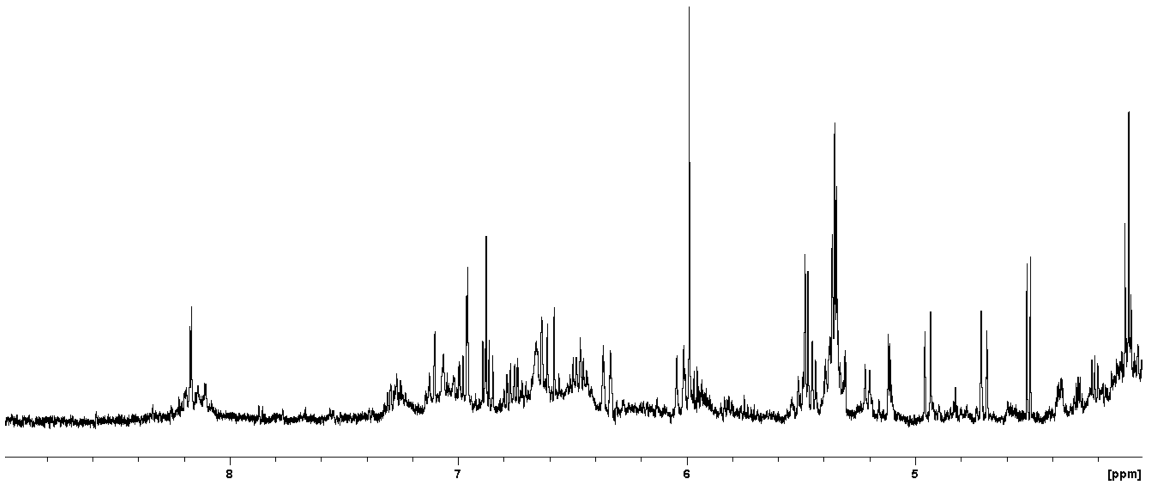

DS 17

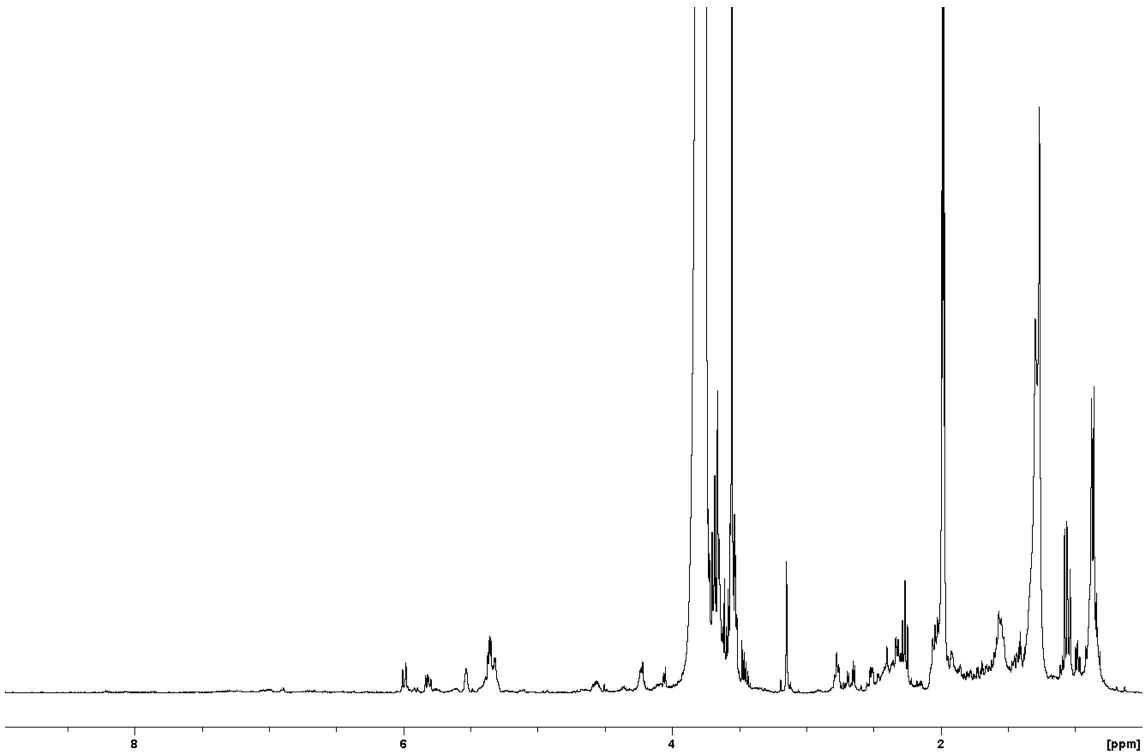

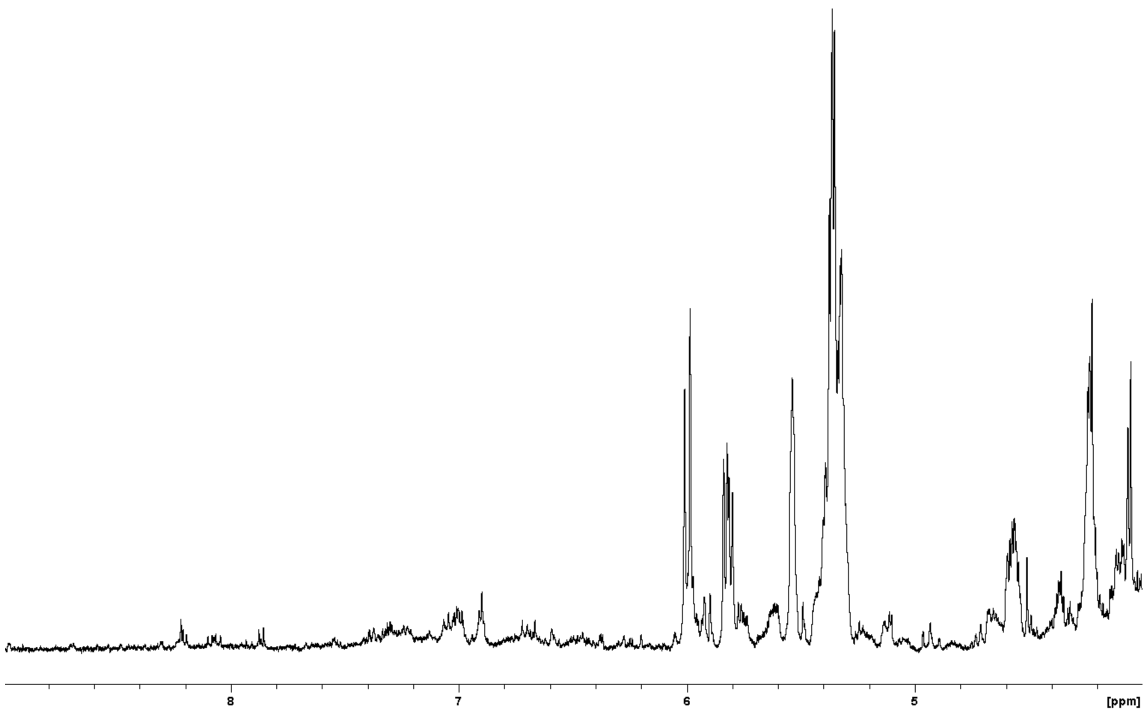

DS 18

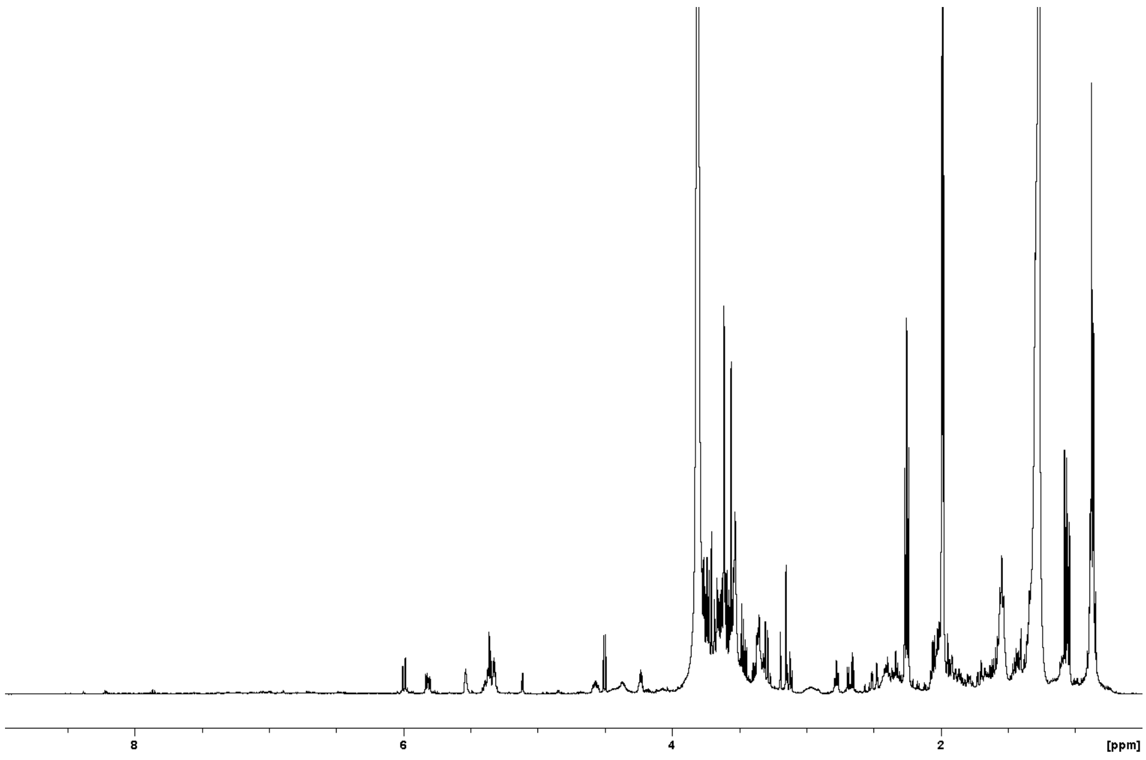

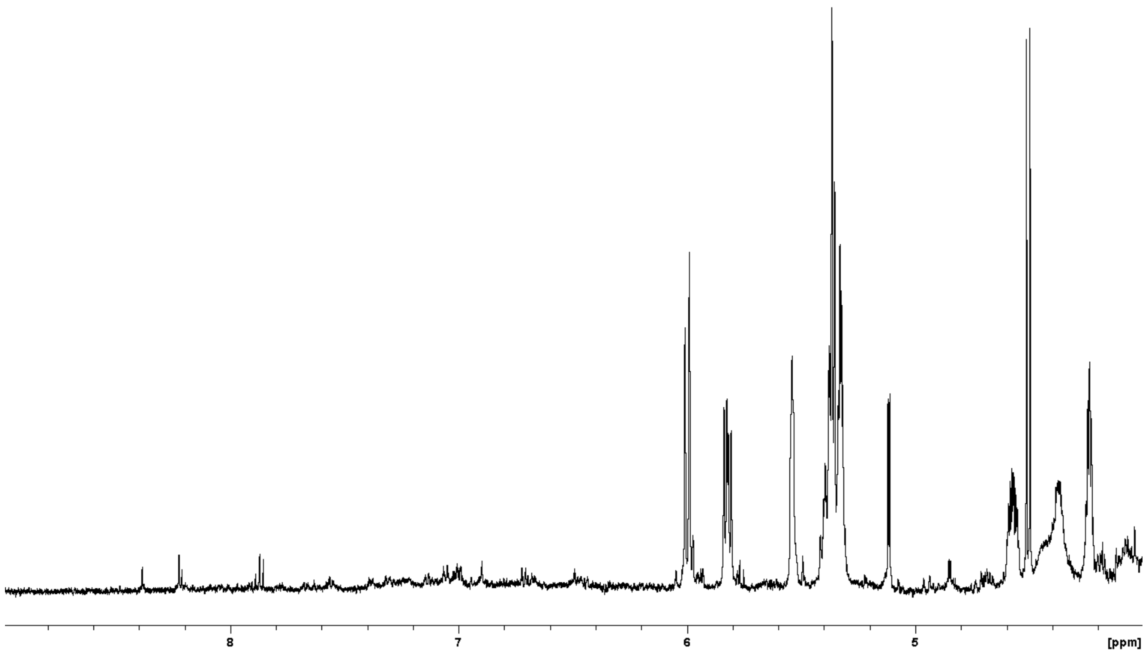

DS 19

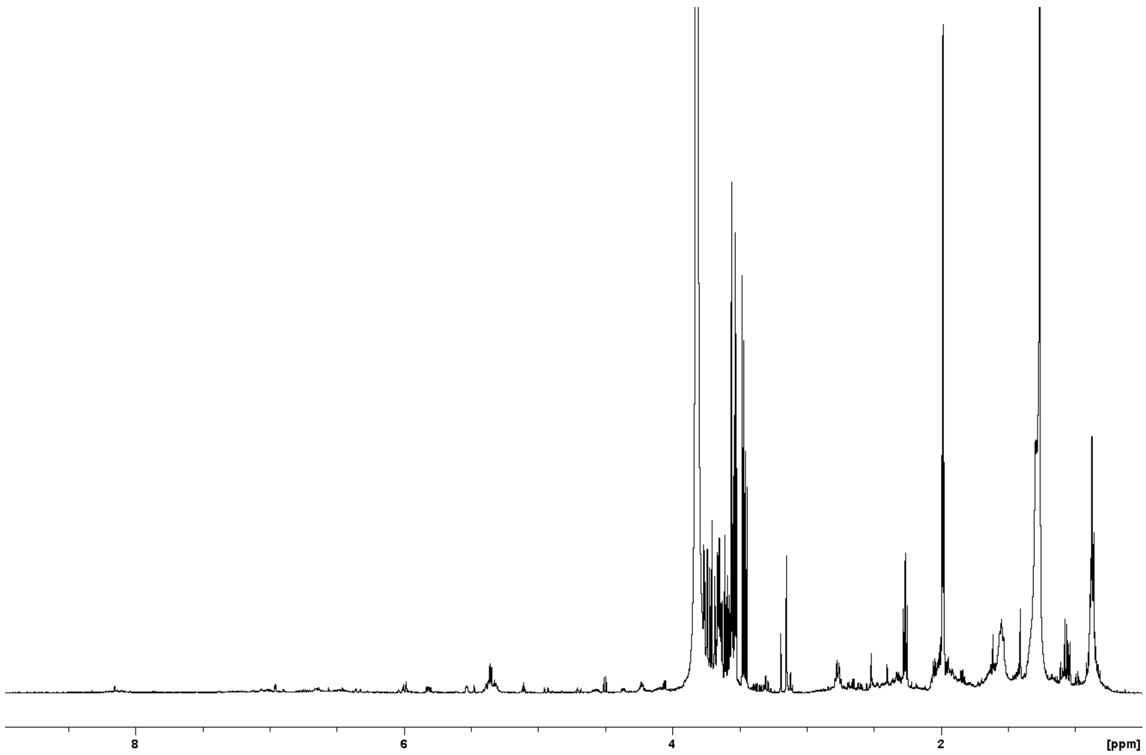

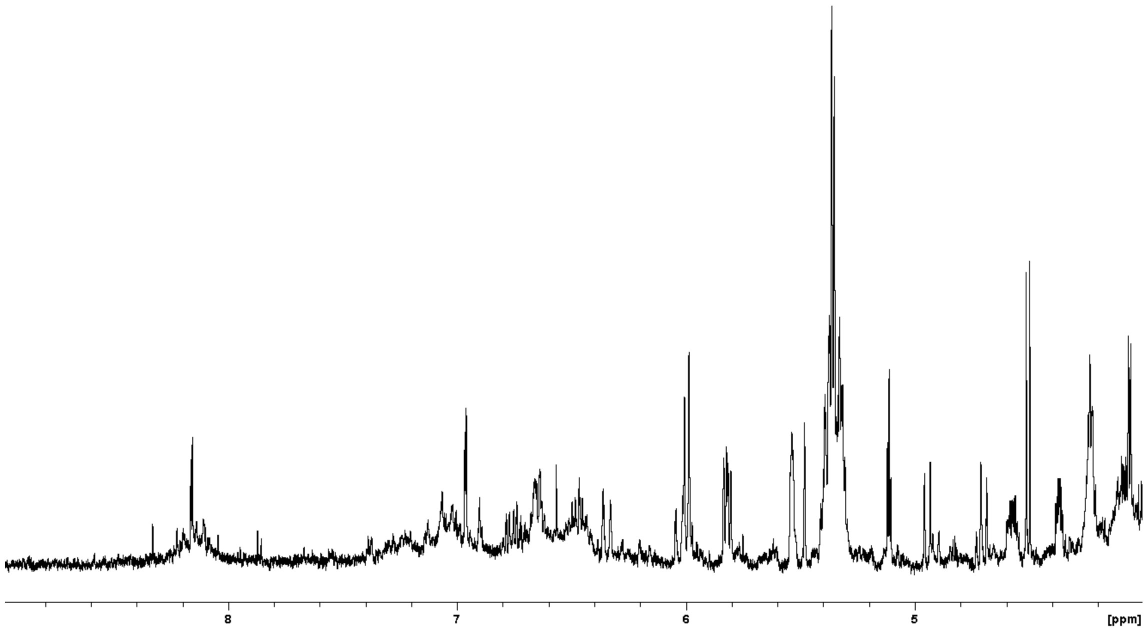

DS 20

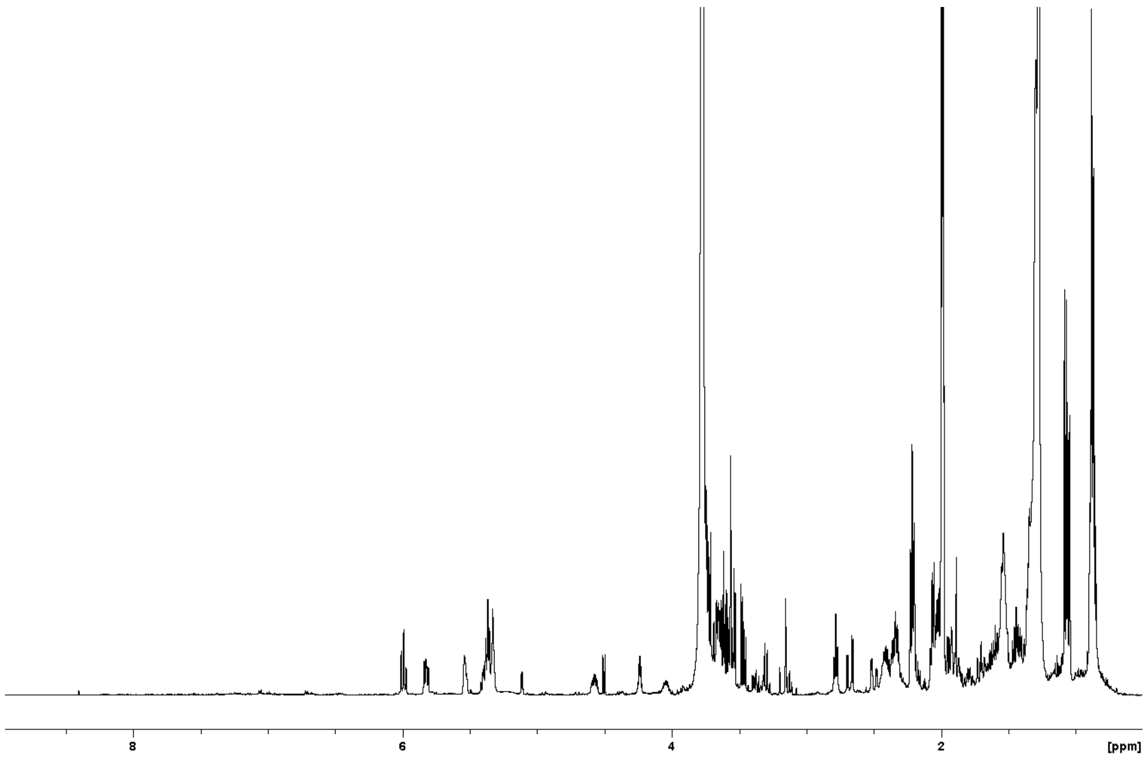

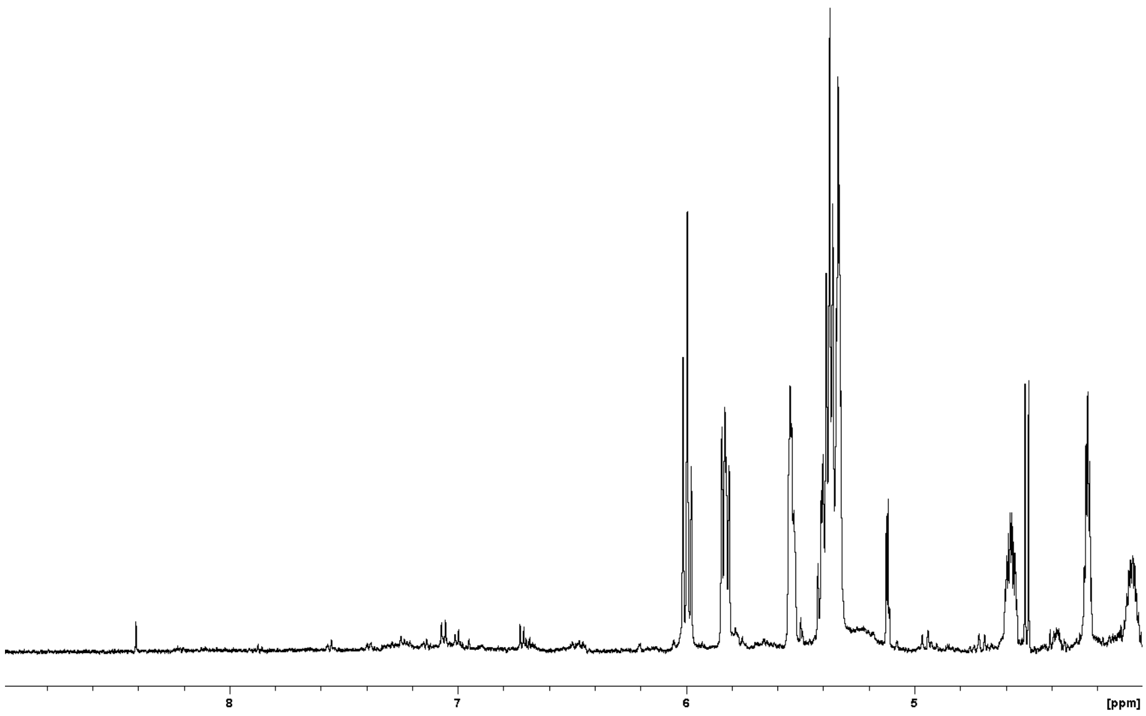

DS 21

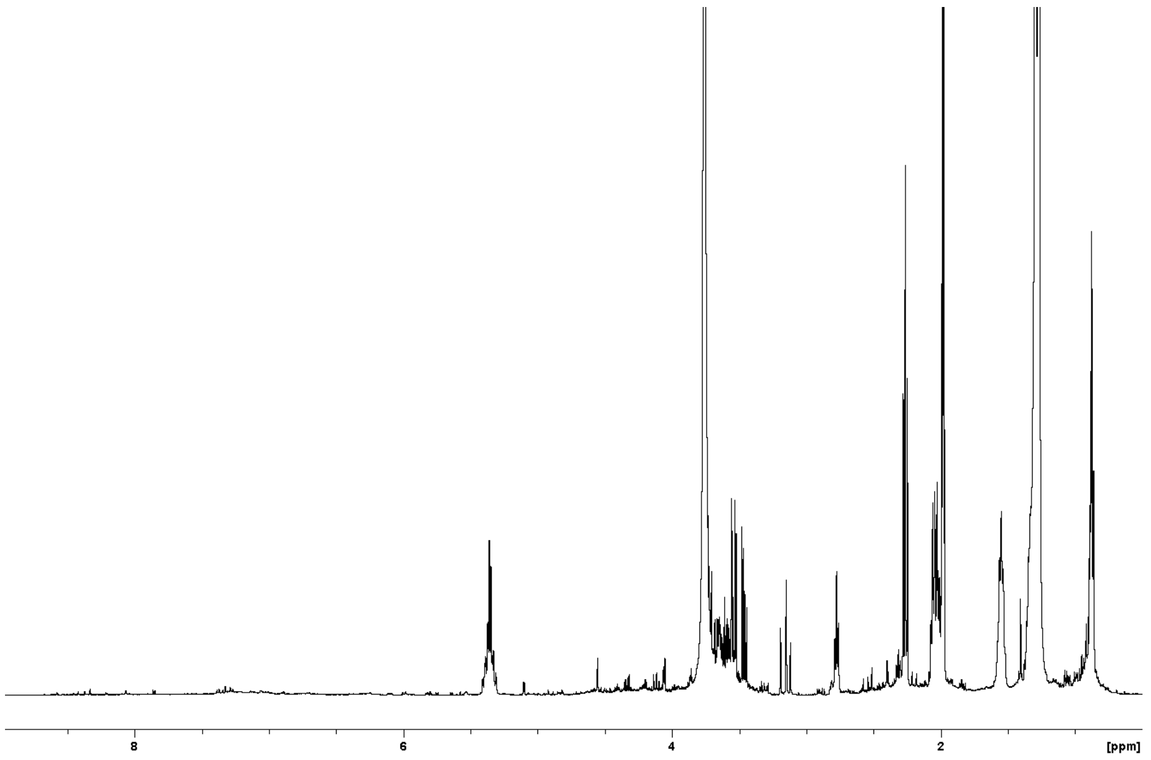

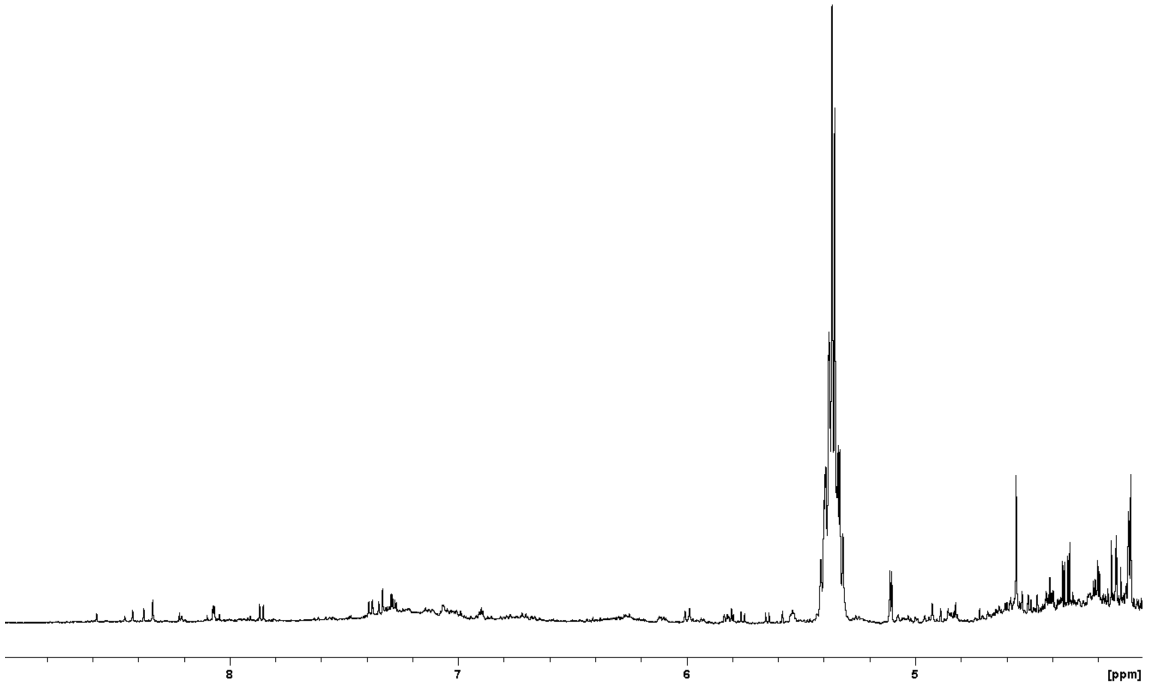

DS 22

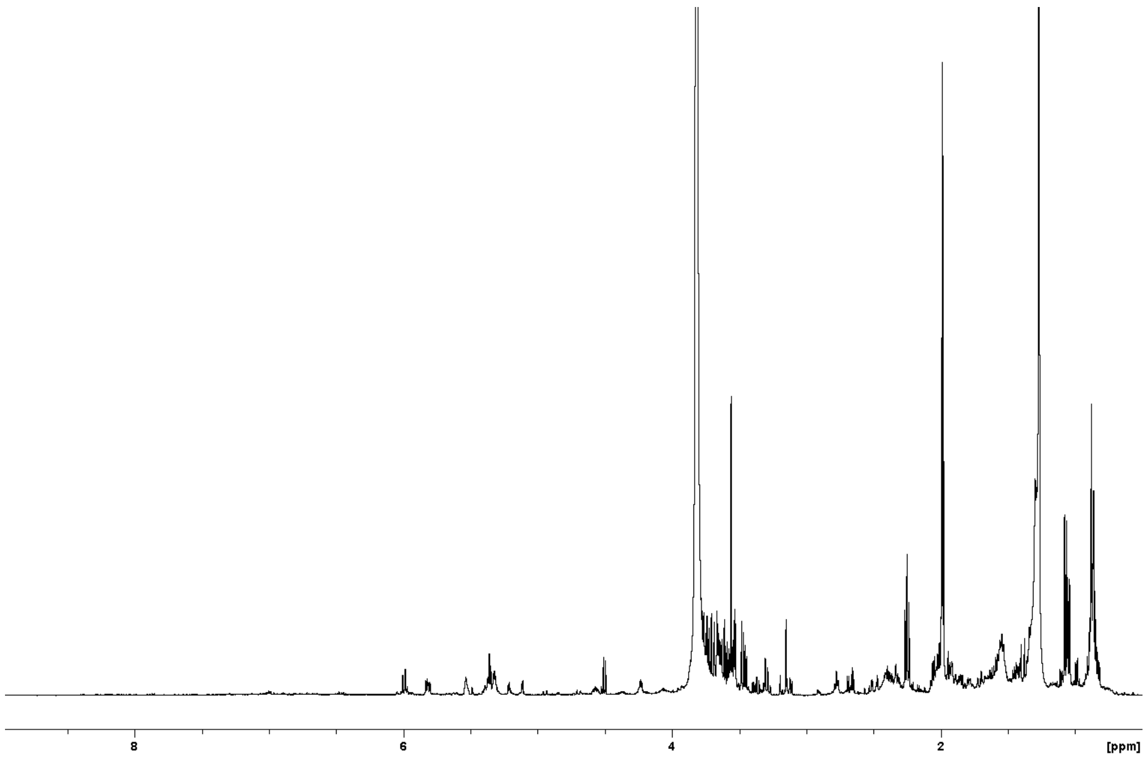

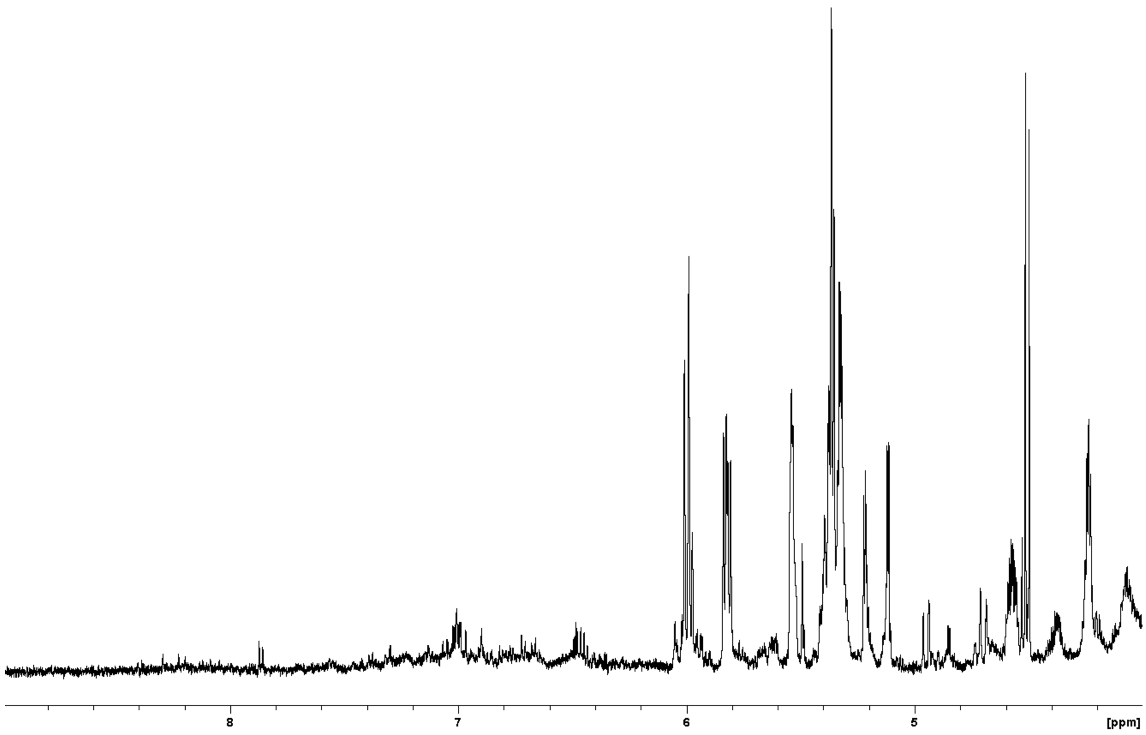

DS 23

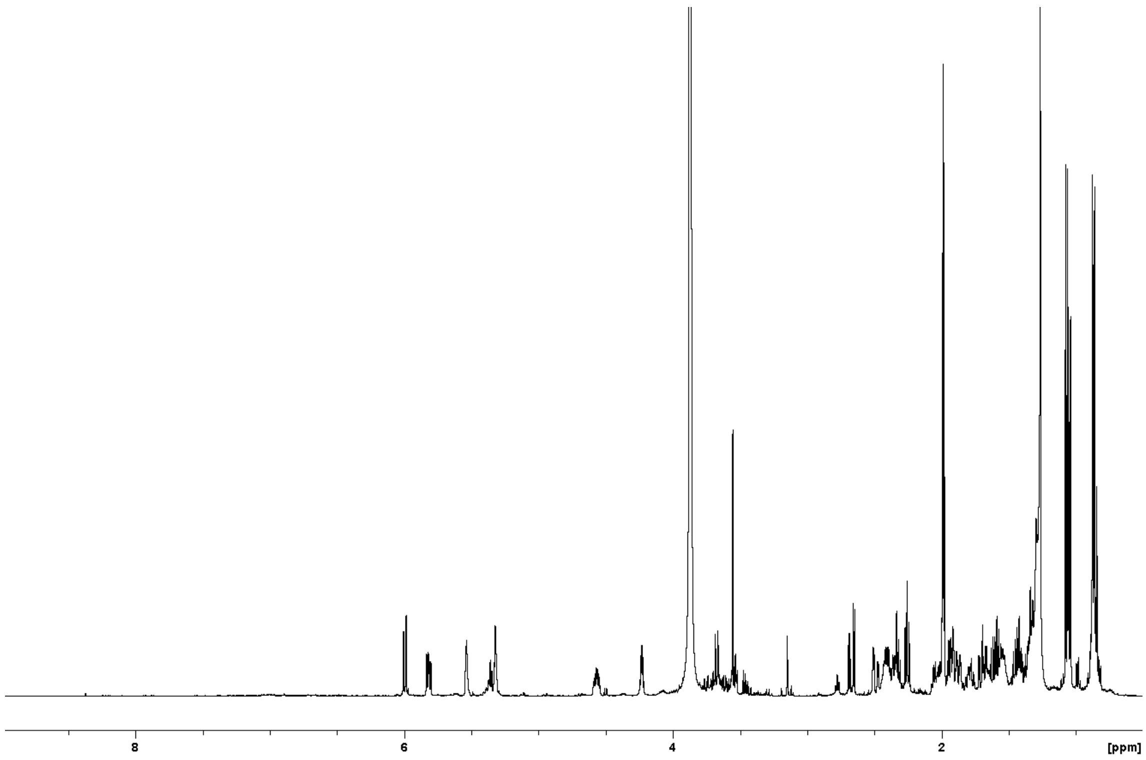

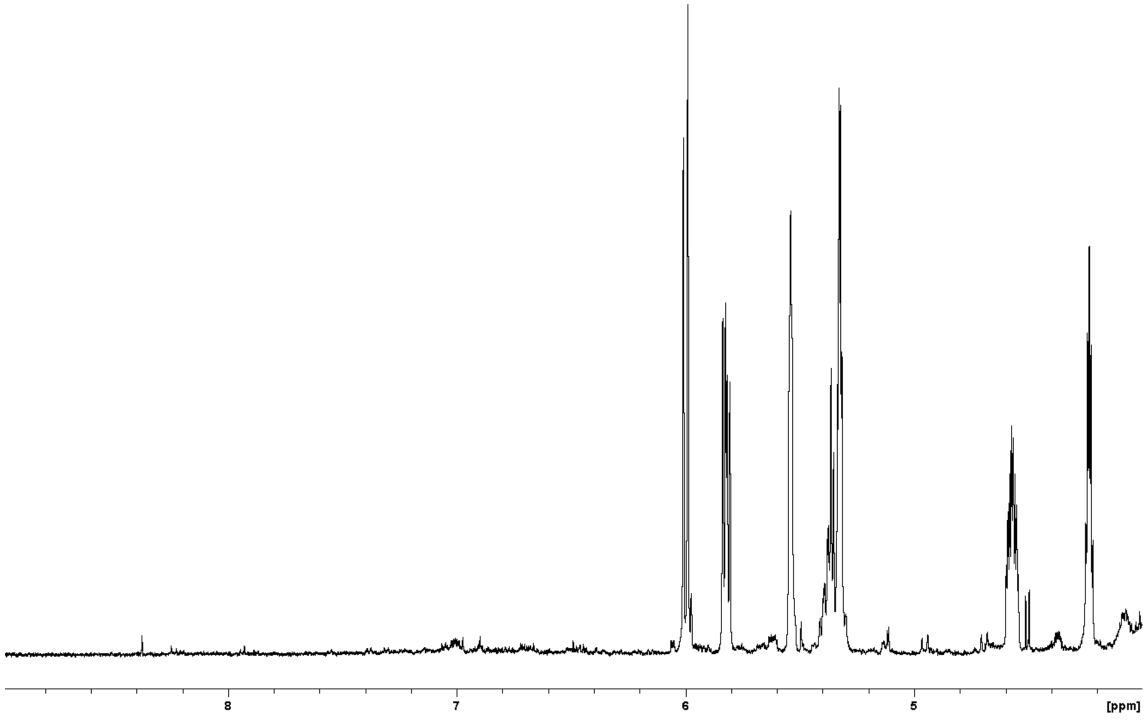

DS 24

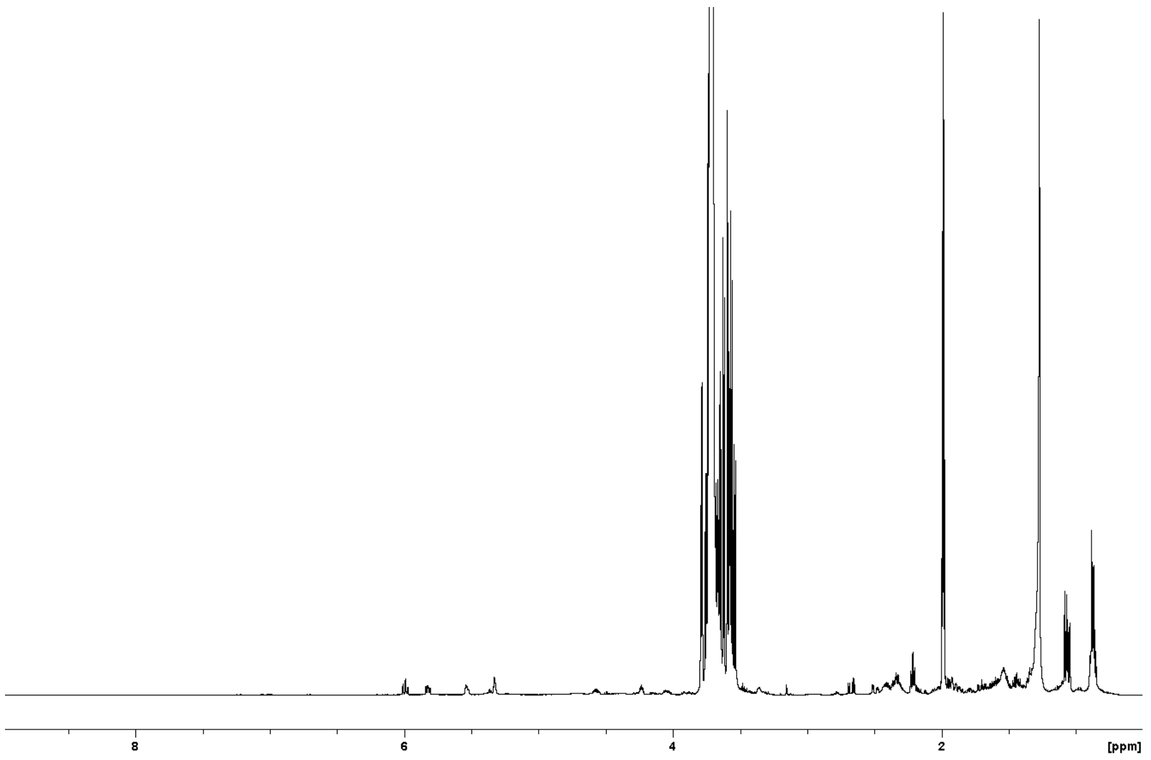

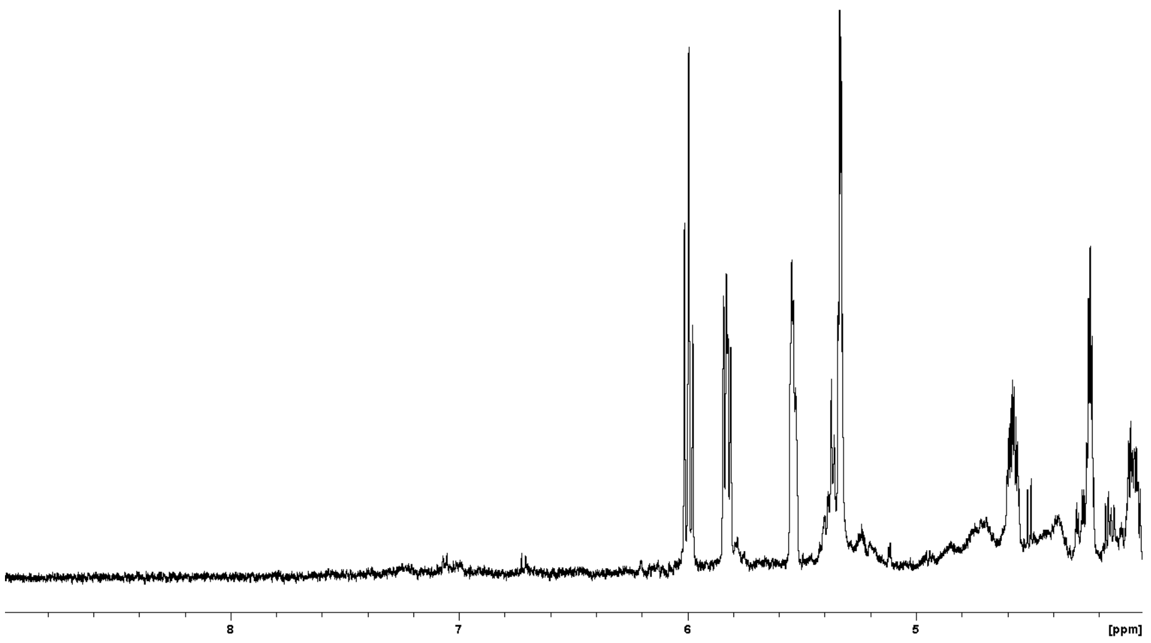

DS 25

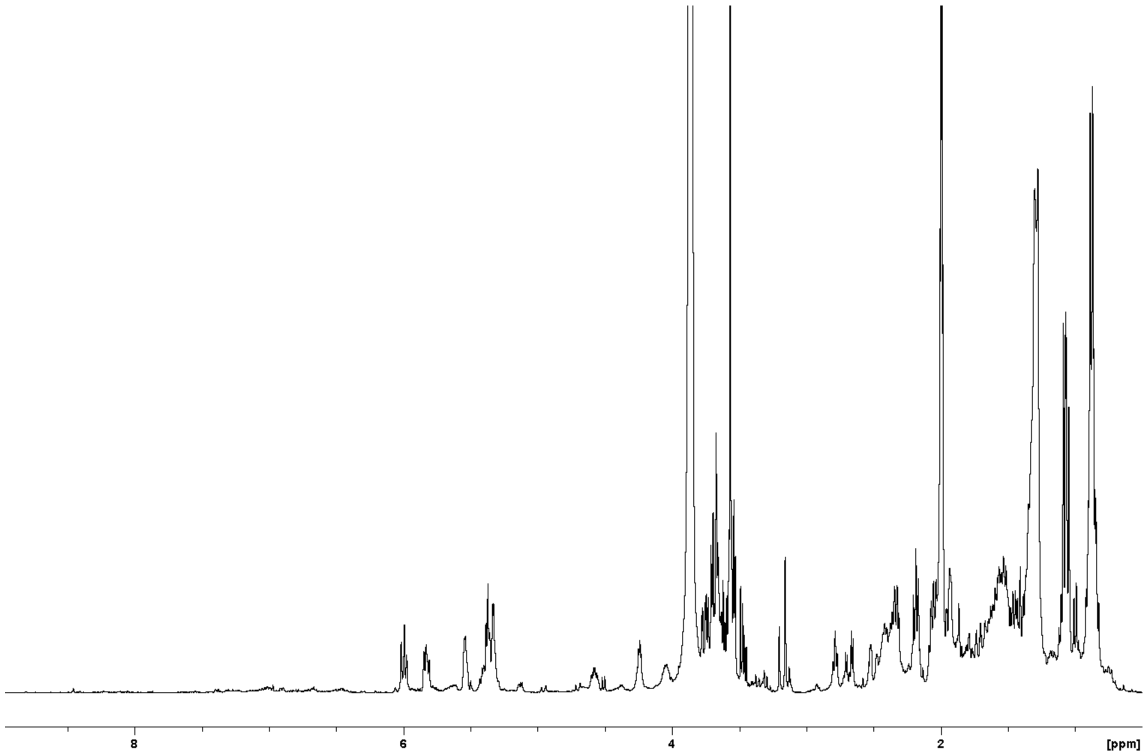

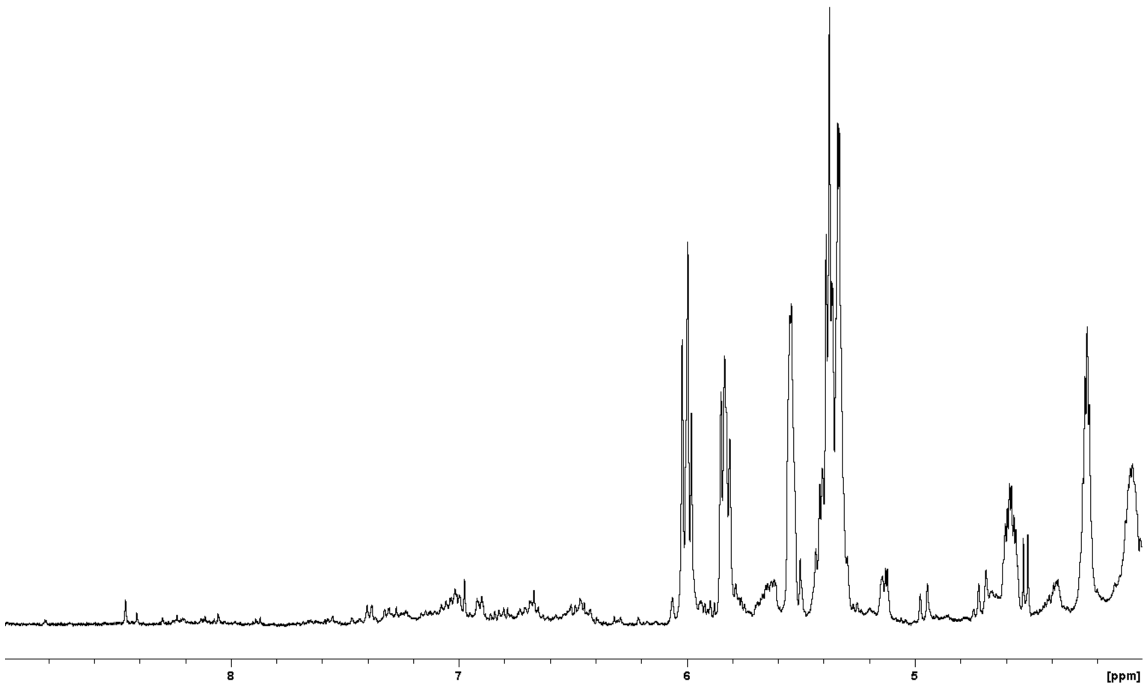

DS 26

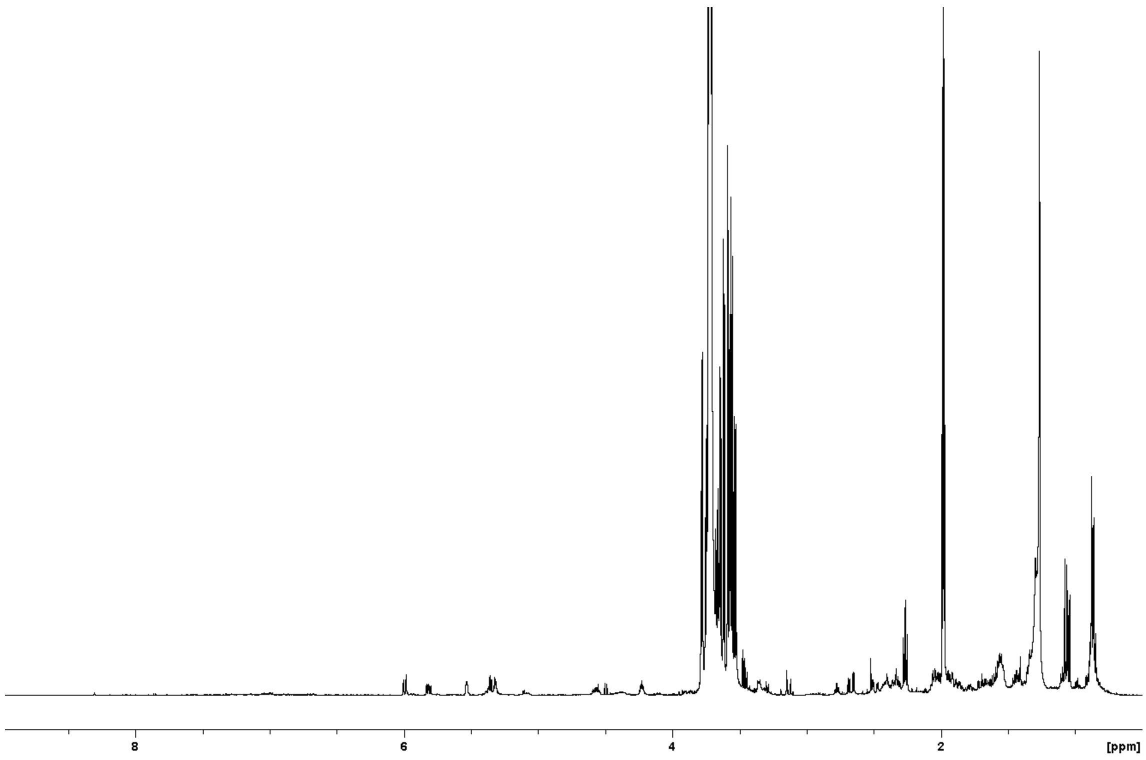

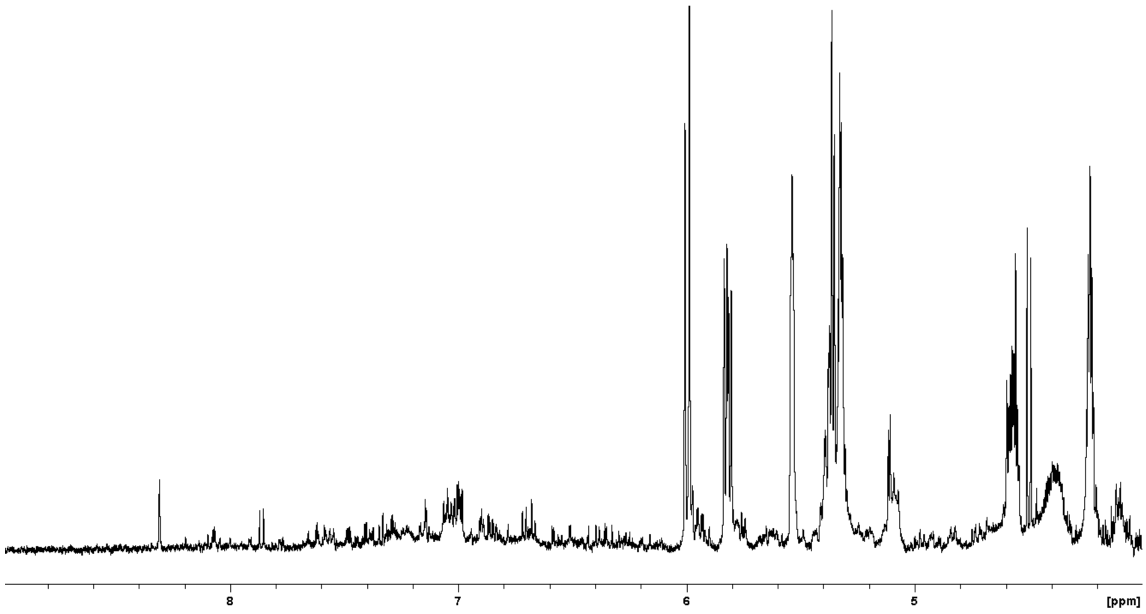

DS 27

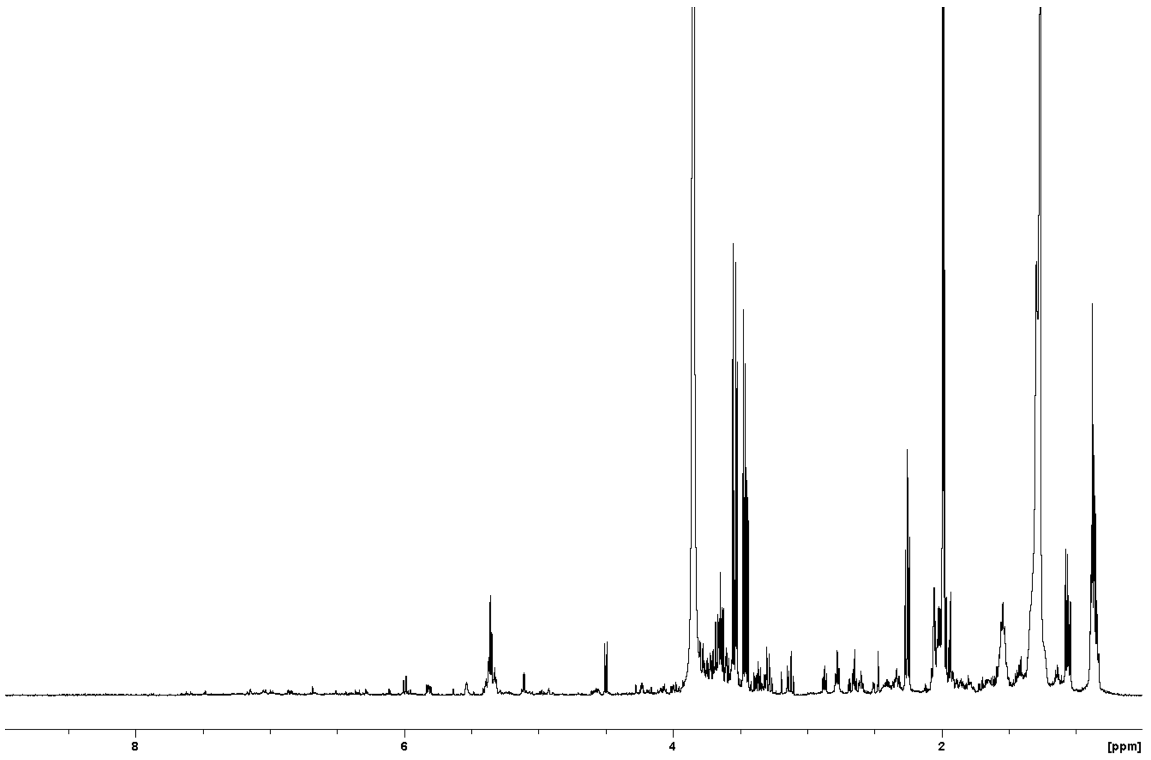

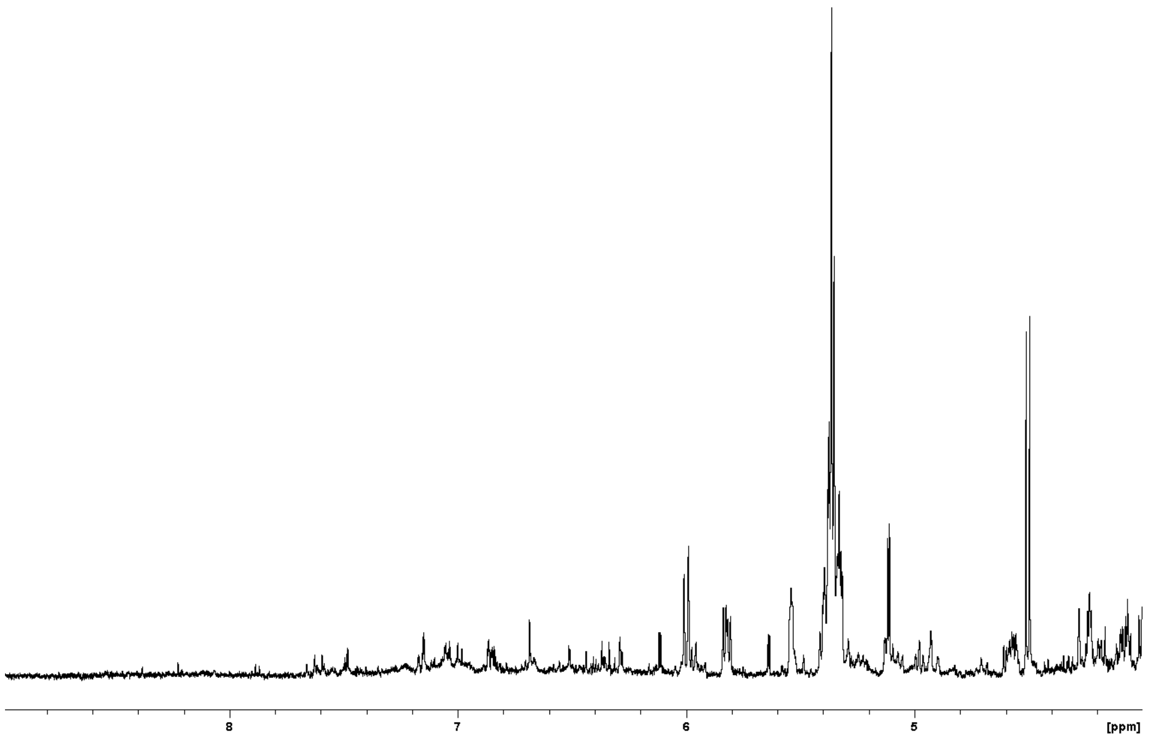

DS 28

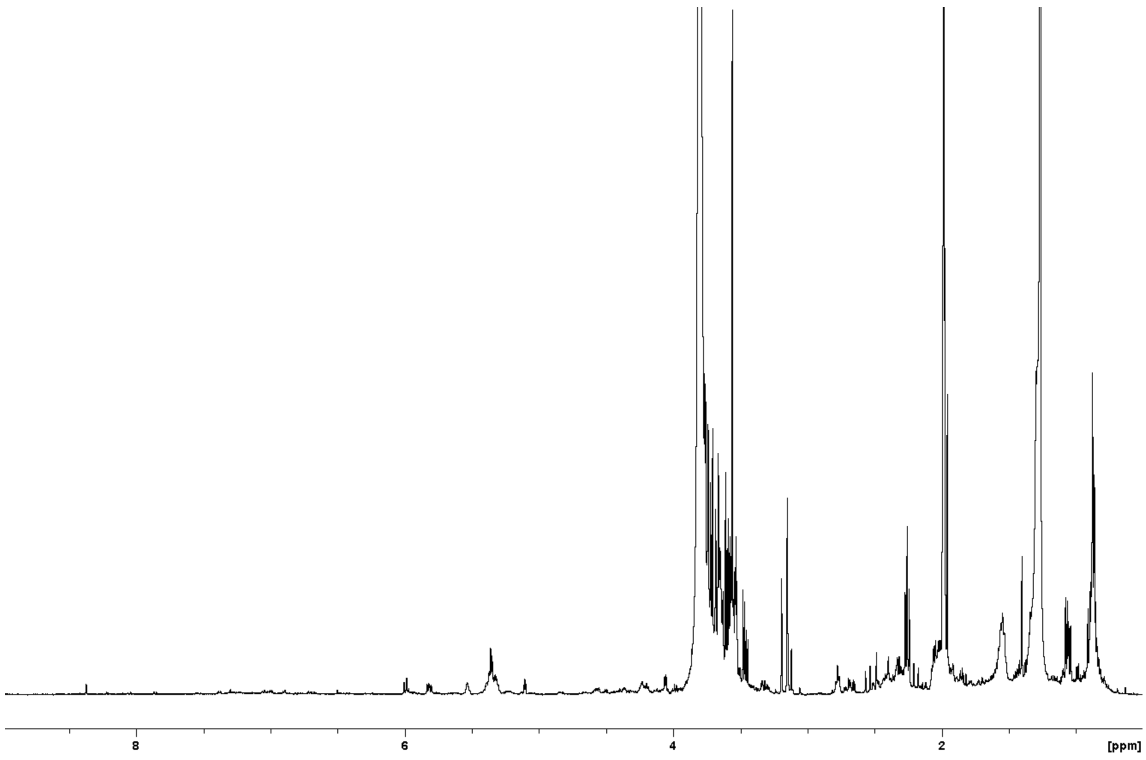

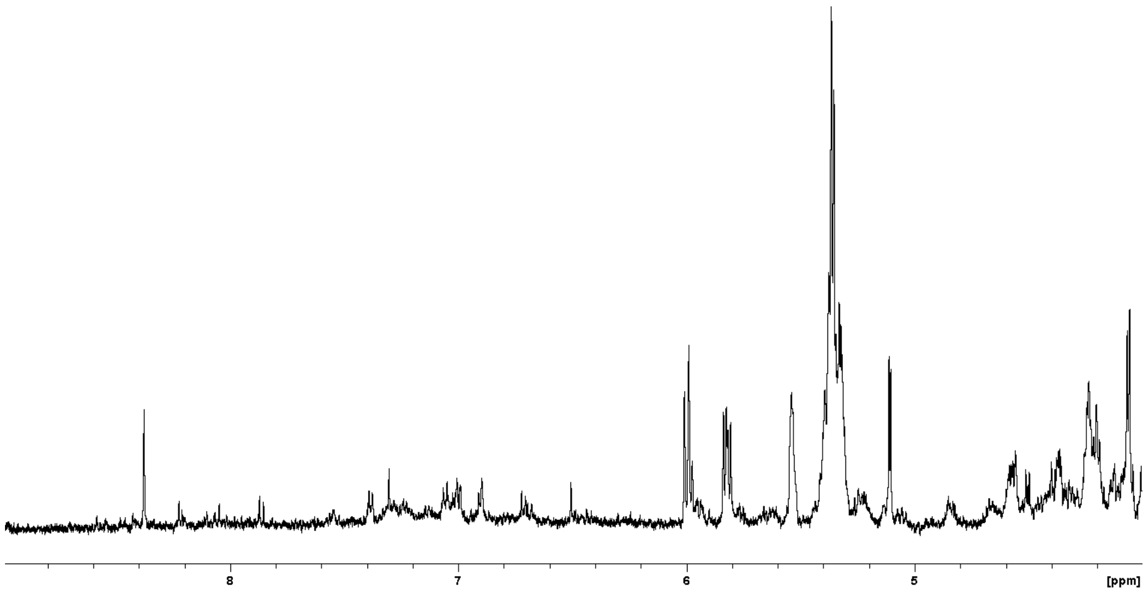

DS 29 No monacolins detected

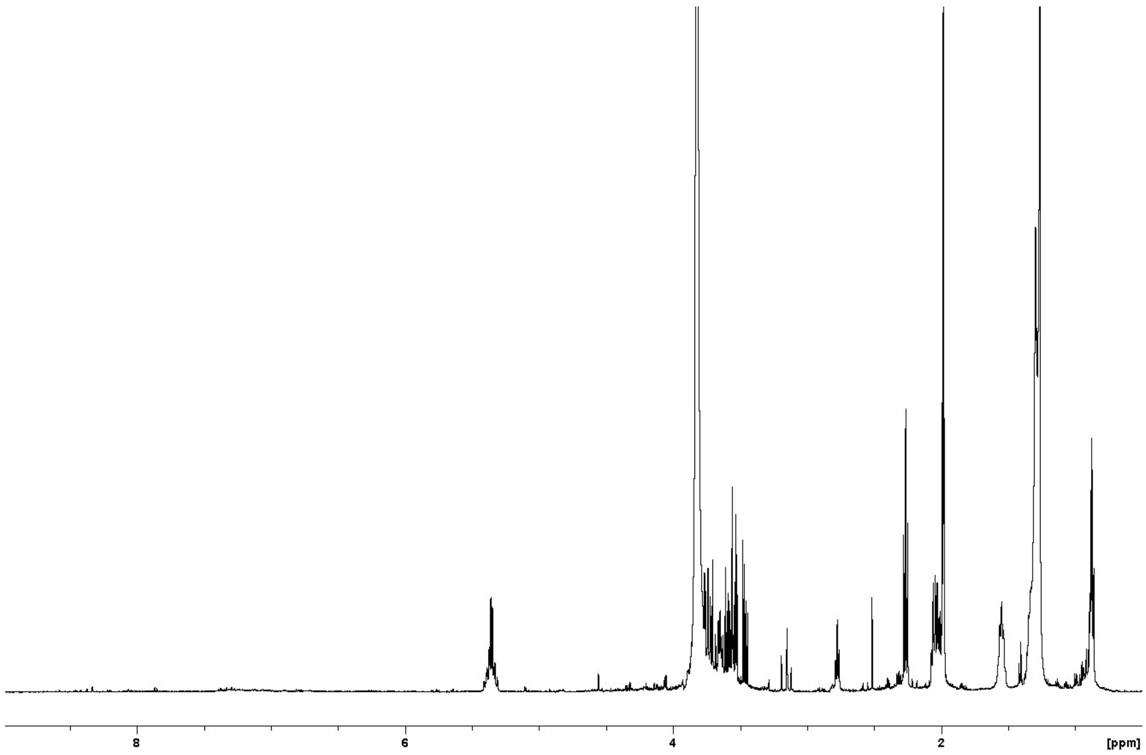

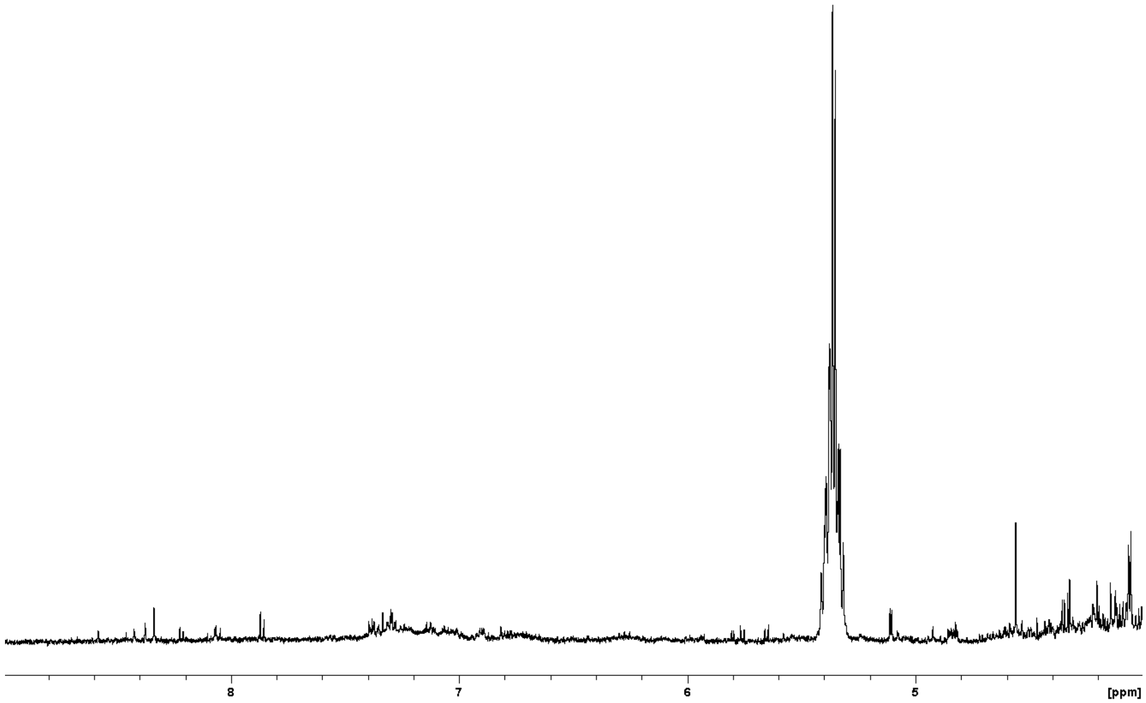

DS 30

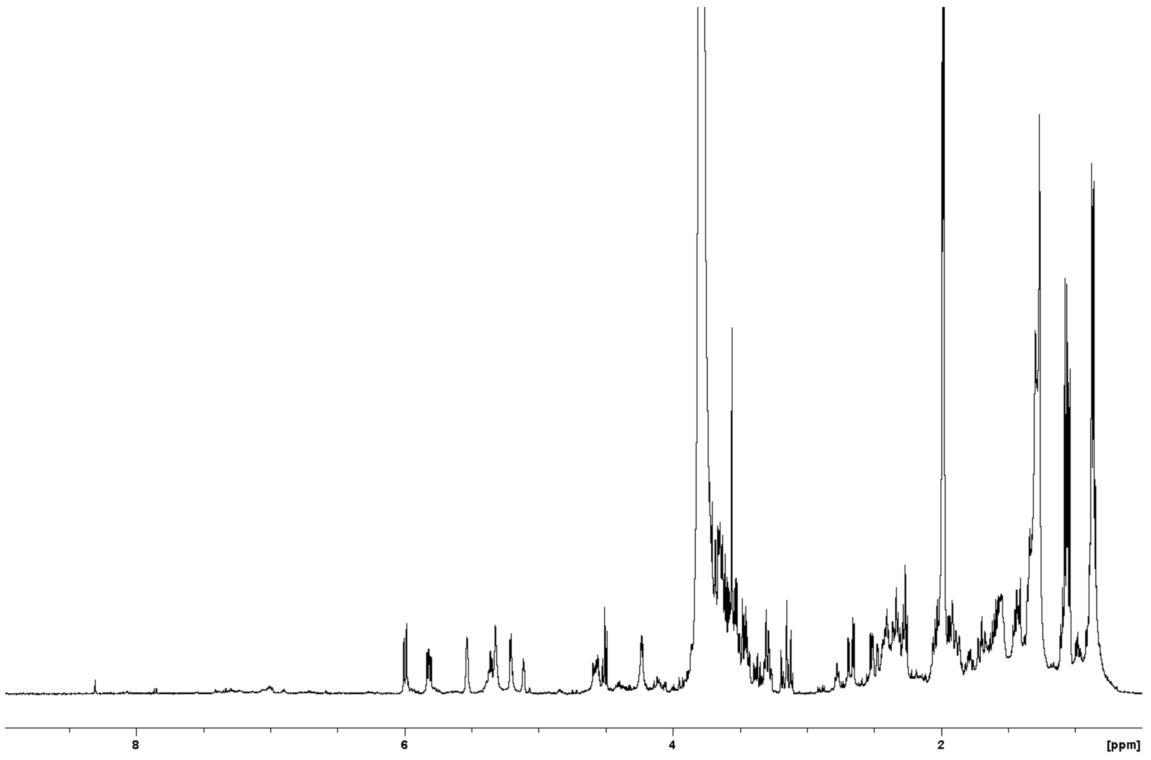

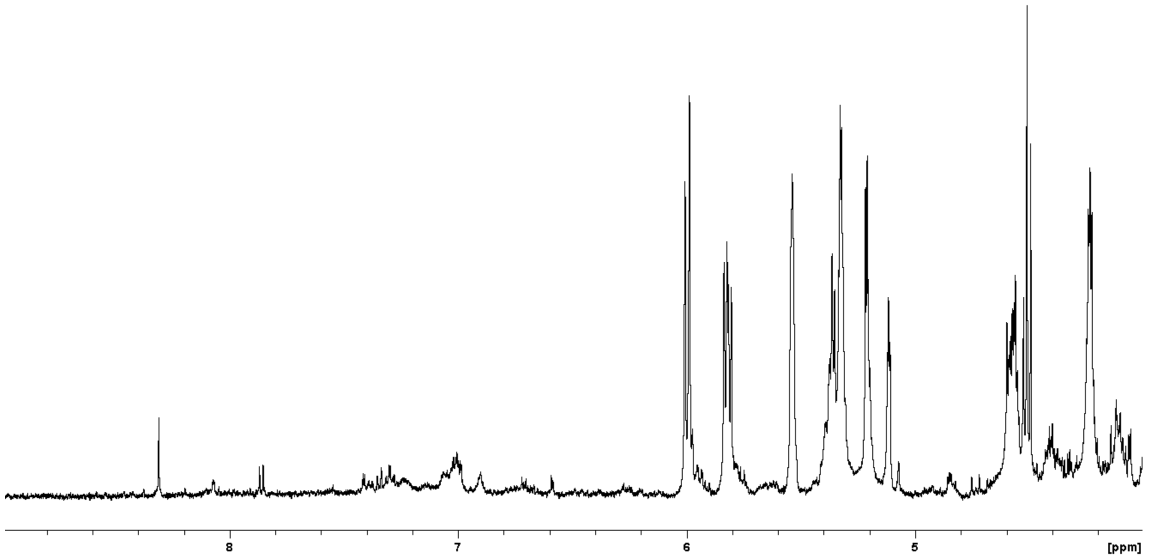

DS 31

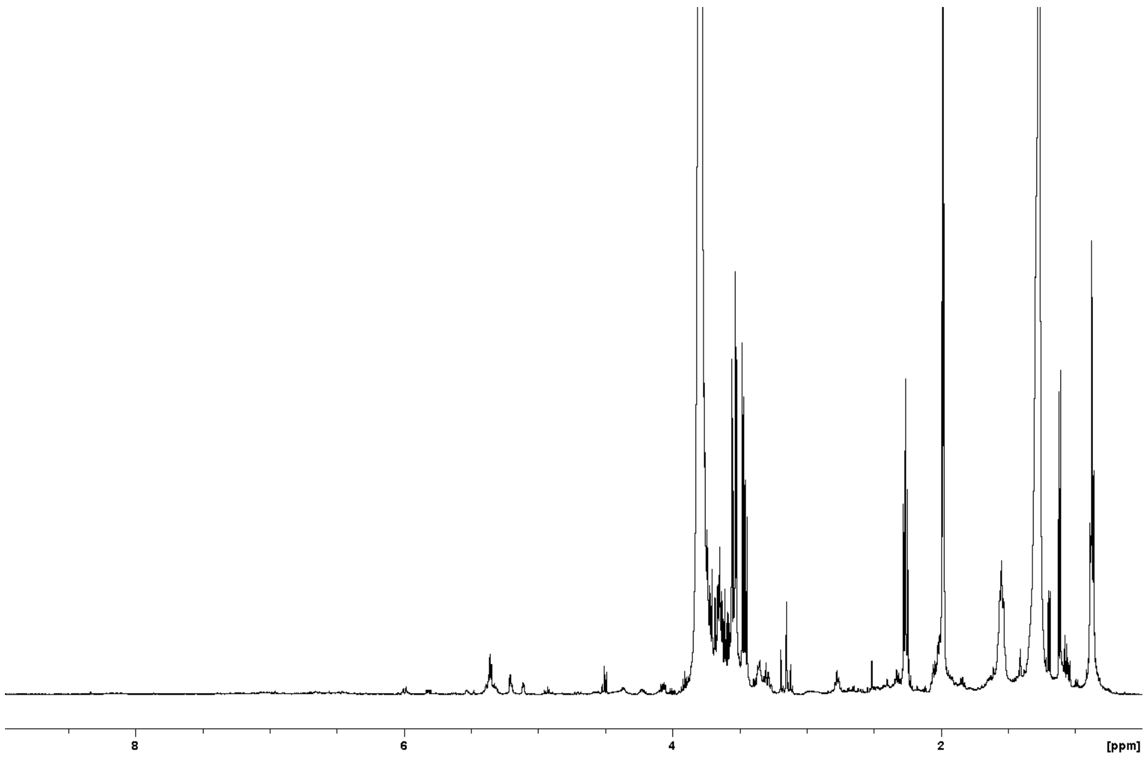

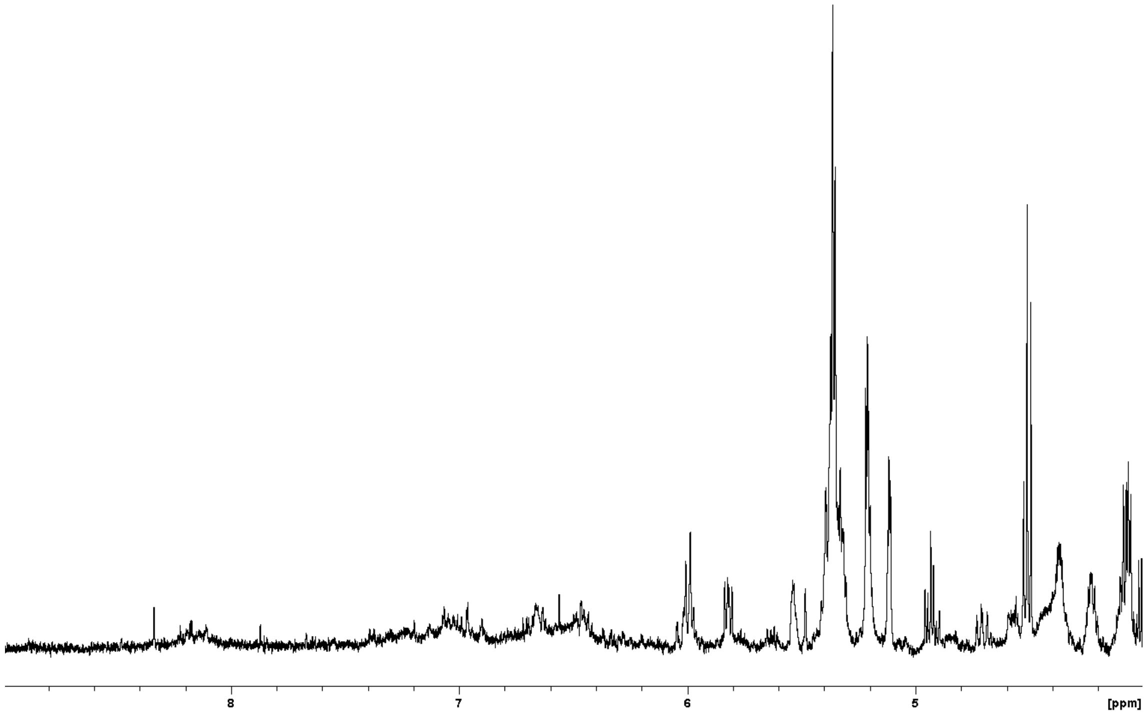

Figure S2. UHPLC chromatograms with UV detection at 238 nm (left) and full scan MS profile in positive ESI mode (right) of 9 RYR dietary supplements. MJ, MN, ML, MK: monacolins J, N, L, K; MLA, MKA: monacolins L, K in hydroxyl acid forms; CP: compactin; DiMK: dihydromonacolin K; DeML, DeMK: dehydromonacolins L,K; Cit: citrinin; Mo: monascin; Moco: monascorubramine; Mocusp: monascuspiloin.

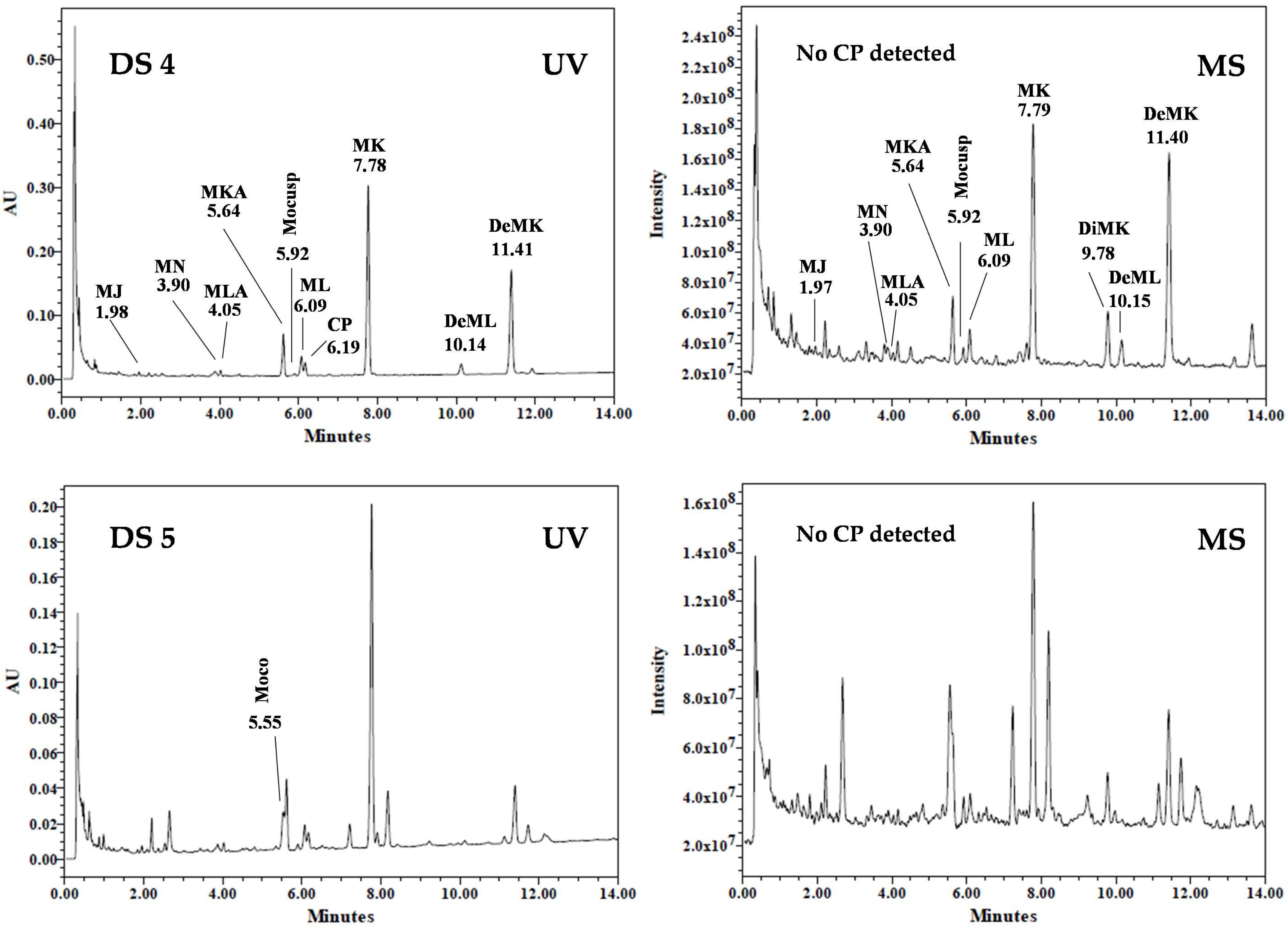
DS 4
UV
AU
MJ 1.98
MN 3.90
MLA 4.05
MKA 5.64
Mocusp 5.92
ML 6.09
CP 6.19
MK 7.78
DeML 10.14
DeMK 11.41
Minutes
No CP detected
MS
Intensity
MJ 1.97
MN 3.90
MLA 4.05
MKA 5.64
Mocusp 5.92
ML 6.09
MK 7.79
DiMK 9.78
DeML 10.15
DeMK 11.40
Minutes
DS 5
UV
AU
Moco 5.55
Minutes
No CP detected
MS
Intensity
Minutes

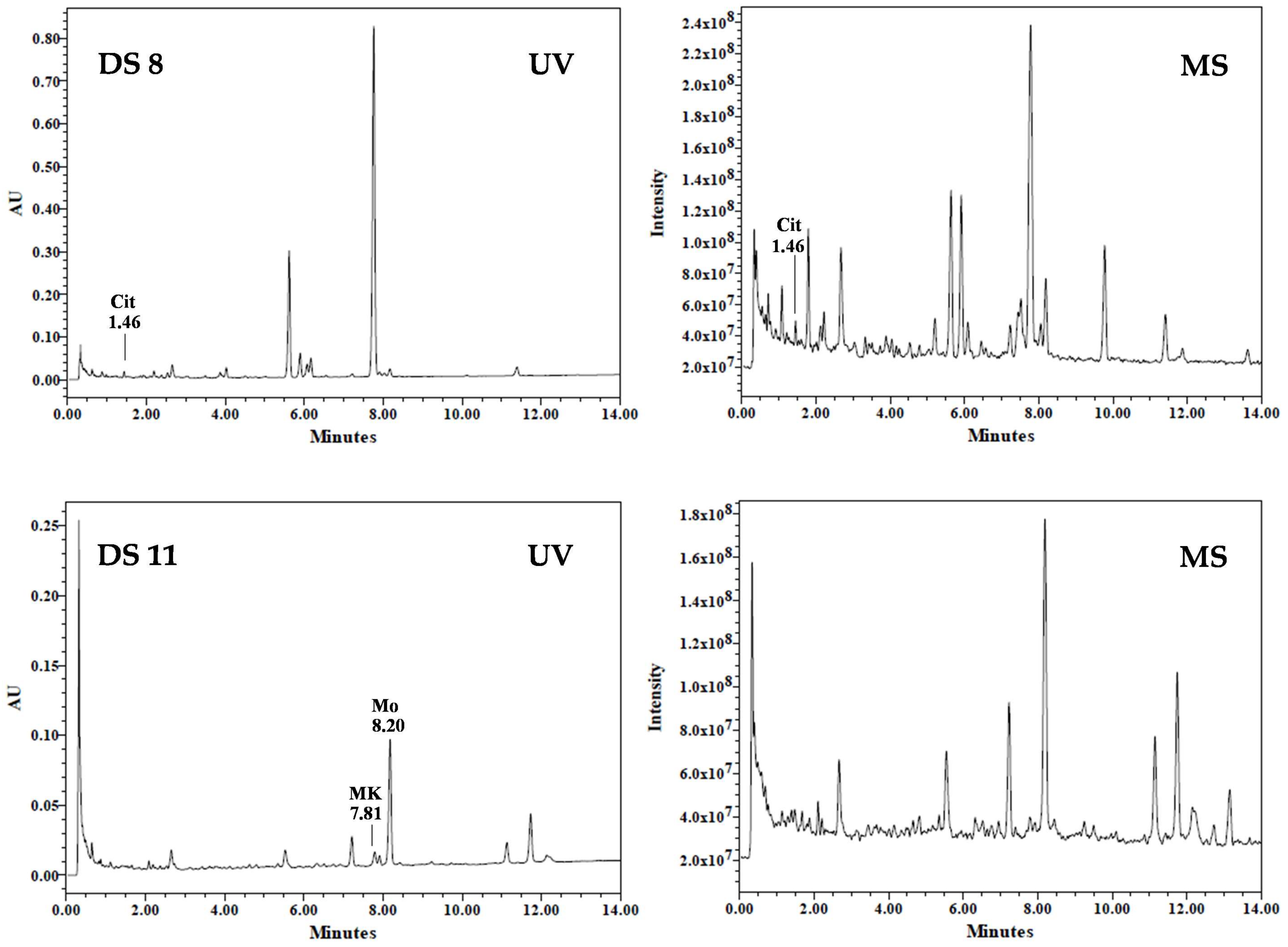
DS 8
UV
AU
Cit
1.46
Minutes
MS
Intensity
Cit
1.46
Minutes
DS 11
UV
AU
Mo
8.20
MK
7.81
Minutes
MS
Intensity
Minutes

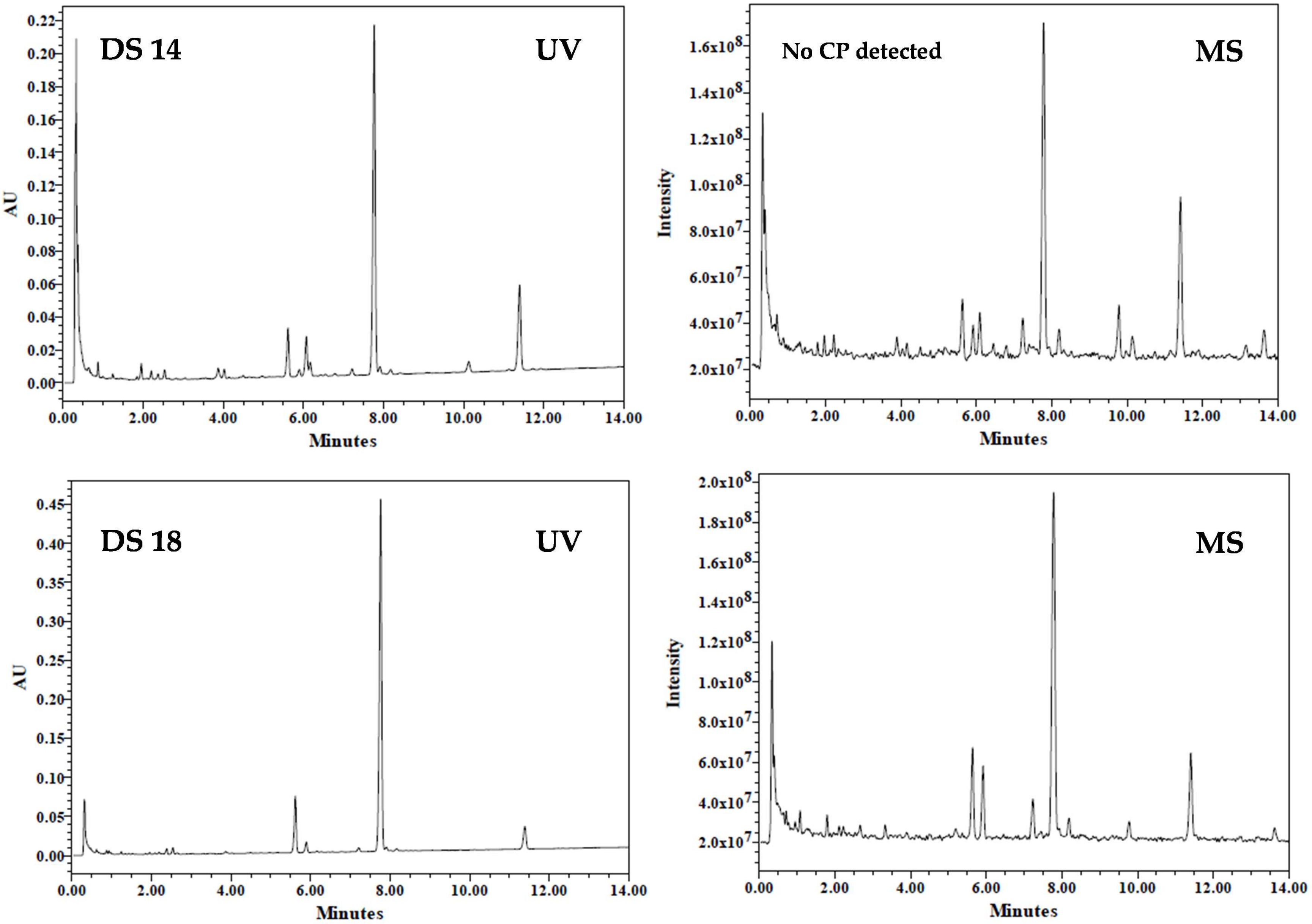
DS 14
UV
AU
Minutes
No CP detected
MS
Intensity
Minutes
DS 18
UV
AU
Minutes
MS
Intensity
Minutes

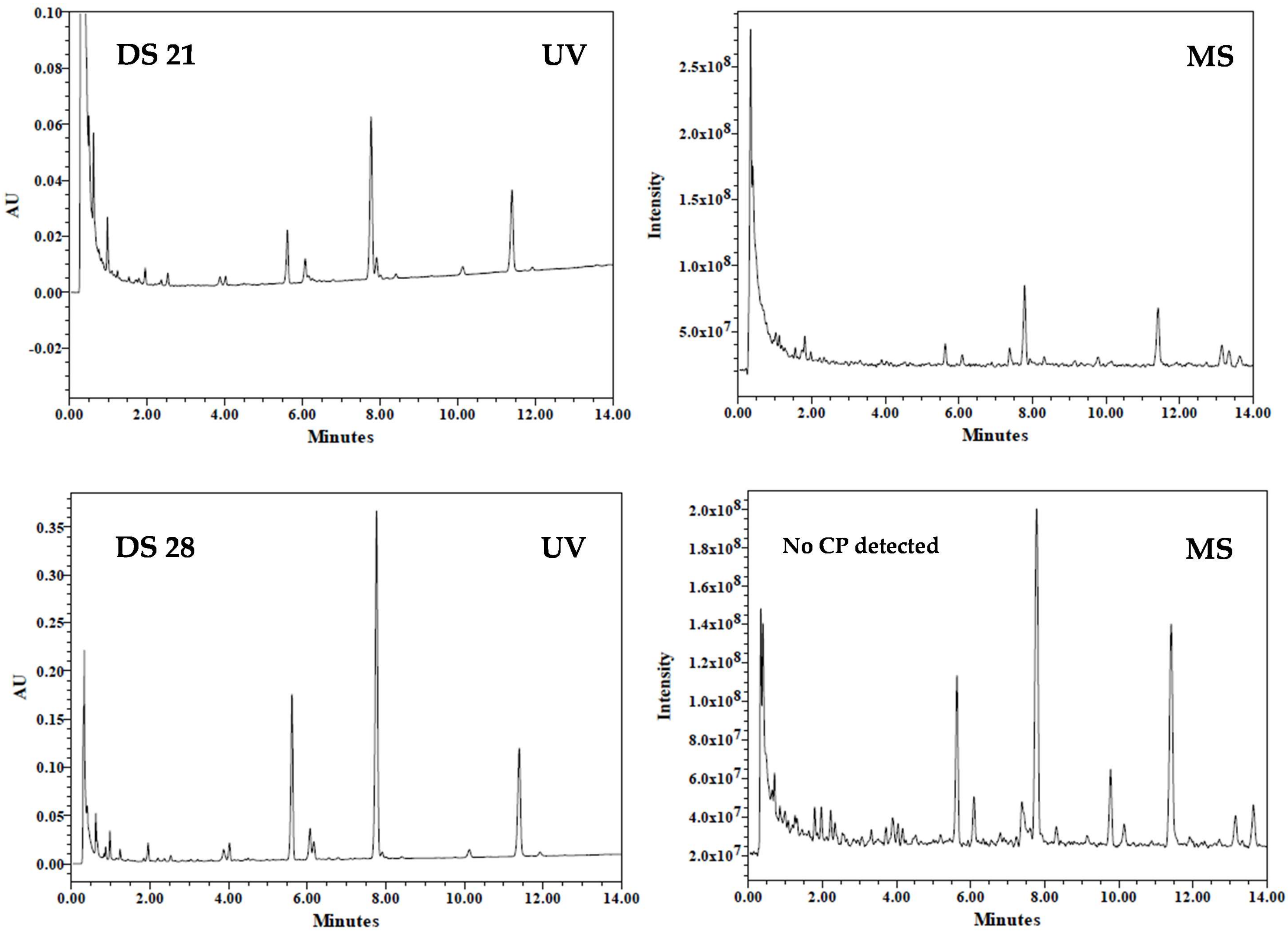
DS 21
UV
AU
Minutes
MS
Intensity
Minutes
DS 28
UV
AU
Minutes
No CP detected
MS
Intensity
Minutes

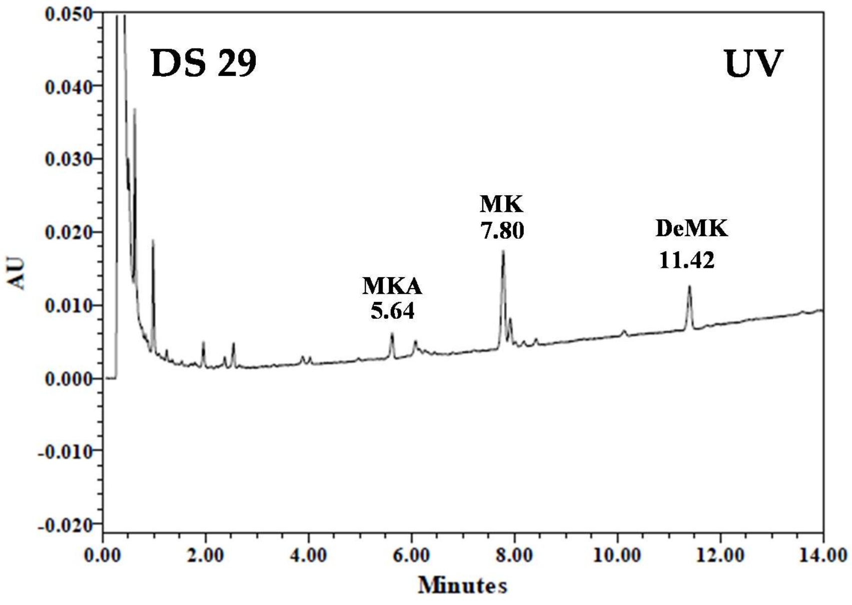
DS 29
UV
MKA
5.64
MK
7.80
DeMK
11.42
AU
Minutes
0.050
0.040
0.030
0.020
0.010
0.000
-0.010
-0.020
0.00
2.00
4.00
6.00
8.00
10.00
12.00
14.00

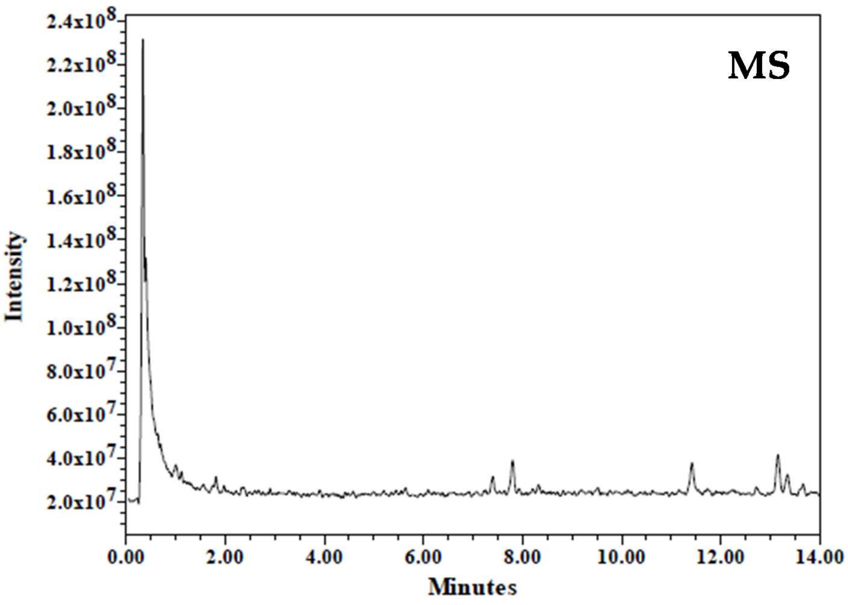
MS
Intensity
Minutes
2.4x10^8
2.2x10^8
2.0x10^8
1.8x10^8
1.6x10^8
1.4x10^8
1.2x10^8
1.0x10^8
8.0x10^7
6.0x10^7
4.0x10^7
2.0x10^7
0.00
2.00
4.00
6.00
8.00
10.00
12.00
14.00
